# Anatomy and osteohistology of the basal hadrosaurid dinosaur *Eotrachodon* from the uppermost Santonian (Cretaceous) of southern Appalachia

**DOI:** 10.7717/peerj.1872

**Published:** 2016-04-14

**Authors:** Albert Prieto-Márquez, Gregory M. Erickson, Jun A. Ebersole

**Affiliations:** 1 School of Earth Sciences, University of Bristol, Bristol, UK; 2 Department of Biological Science, Florida State University, Tallahassee, Florida, USA; 3 McWane Science Center, Birmingham, Alabama, USA

**Keywords:** Anatomy, Evolution, Dinosaur, Hadrosaurid, Cretaceous, Appalachia, Histology

## Abstract

The cranial and postcranial anatomy of the basal hadrosaurid dinosaur *Eotrachodon orientalis*, from the uppermost Santonian of southern Appalachia (southeastern U.S.A.), is described in detail. This animal is the only known pre-Campanian non-lambeosaurine hadrosaurid, and the most complete hadrosauroid known from Appalachia. *E. orientalis* possesses a mosaic of plesiomorphic and derived characters in the context of Hadrosauroidea. Characters shared with basal hadrosauroids include a short and sloping maxillary ectopterygoid shelf, caudally prominent maxillary jugal process, one functional tooth per alveolus on the maxillary occlusal plane, a jugal rostral process with a shallow caudodorsal margin and medioventrally facing articular facet, a vertical dentary coronoid process with a poorly expanded apex, and tooth crowns with accessory ridges. Derived characters shared with other hadrosaurids include a circumnarial depression compartmented into three fossae (as in brachylophosaurins and *Edmontosaurus*), a thin everted premaxillary oral margin (as in *Gryposaurus*, *Prosaurolophus*, and *Saurolophus*), and a maxilla with a deep and rostrocaudally extensive rostrodorsal region with a steeply sloping premaxillary margin (as in *Gryposaurus*). *Eotrachodon orientalis* differs primarily from the other hadrosauroid from the Mooreville Chalk of Alabama, *Lophorhothon atopus*, in having a slender and crestless nasal whose caudodorsal margin is not invaded by the circumnarial depression. *Hadrosaurus foulkii*, the only other known hadrosaurid from Appalachia, is distinct from *E. orientalis* in having dentary teeth lacking accessory ridges and a dorsally curved shaft of the ischium. A histological section of the tibia of the *E. orientalis* holotype (MSC 7949) suggests that this individual was actively growing at the time of death and, thus, had the potential to become a larger animal later in development.

## Introduction

Hadrosaurids are facultatively bipedal ornithopod dinosaurs characterized by densely packed dental batteries (Erickson et al., 2012), mediolaterally expanded rostra (Morris, 1970), and hypertrophied nasal passages that are often associated to elaborated supracranial crests (Evans, 2006; Prieto-Márquez, 2010a). During the Late Cretaceous (Santonian–late Maastrichtian) these animals became a major component of the terrestrial vertebrate faunas of Eurasia, the Americas, and Antarctica (Prieto-Márquez, 2010b).

Recently, a new genus and species of hadrosaurid, *Eotrachodon orientalis* Prieto-Márquez, Erickson & Ebersole (2016) was erected from uppermost Santonian strata of Alabama, southeastern United States. During the Late Cretaceous, this region was located in Appalachia, a continent that was separated from the western Laramidia by the Western Interior Seaway (Gates, Prieto-Márquez & Zanno, 2012). Autapomorphies of *E. orientalis* include: 1) a tripartite circumnarial depression that is divided longitudinally into dorsal and ventral fossae, with the latter subdivided into caudoventral and lightly incised rostroventral fossae; 2) dorsal fossa of circumnarial depression above bony naris extending further caudally than caudal extent of caudoventral fossa; 3) caudodorsal region of circumnarial depression above bony naris excavating lateral nasal surface, deeply rostrally but gradually diminishing caudally; 4) premaxillary lateral process abruptly deflected ventrally, and forming a 165° angle with long axis of circumnarial depression; 5) maxilla with subtriangular joint surface for jugal that is more laterally than dorsally-facing and prominent dorsal jugal tubercle projected caudally; and finally 6) a steeply down-warped sagittal crest of the parietal substantially elevated above the temporal bar (Prieto-Márquez, Erickson & Ebersole, 2016).

*Eotrachodon orientalis* is critical for understanding the early evolution of the clade hadrosaurids because it: 1) is the first known pre-Campanian non-lambeosaurine hadrosaurid; 2) is the most complete Appalachian hadrosauroid known and the only hadrosaurid from this landmass for which the skull has been recovered, (displaying diagnostic elements never before found in the other hadrosauroid taxa from the region); 3) occupies a basal position within the Hadrosauridae, sister taxon to the Saurolophidae (the clade consisting of the Saurolophinae and the Lambeosaurinae; Prieto-Márquez, Erickson & Ebersole, 2016); and 4) documents the acquisition of a derived circumnarial structure (Prieto-Márquez & Wagner, 2014) as early as the late Santonian, prior to the evolution of the fully derived feeding apparatus that characterizes saurolophid hadrosaurids and before the split between the hollow-crested lambeosaurines and the solid-crested and unadorned saurolophines (Prieto-Márquez, Erickson & Ebersole, 2016).

Originally, Prieto-Márquez, Erickson & Ebersole (2016) provided only a cursory description of *Eotrachodon orientalis*. Here, we thoroughly describe the anatomy of this taxon, including the osteology of the neurocranium and the axial and appendicular skeleton. We also provide additional information on the geological setting and occurrence of *E. orientalis*, a detailed comparative osteology with all other Appalachian hadrosauroids, and greater insights into the developmental stage of the holotype specimen.

## Material and Phylogenetic Framework

*Eotrachodon orientalis* is known from a single individual (MSC 7949) that includes a well-preserved, nearly complete, disarticulated skull and mandible, as well as part of the vertebral column and appendicular skeleton. We estimated the body length of MSC 7949 at between 4 and 5.1 m. These values were derived from measurements of the length of the skull and available vertebral centra. To account for missing vertebrae, we multiplied the minimal central length within each cervical, dorsal and sacral series by the minimum and maximum number of vertebrae reported in hadrosaurids. Due to the variability of vertebral counts within various hadrosaurid genera, low and high counts were considered to provide size range estimates. These counts were as follows: 13 (Horner, Weishampel & Forster, 2004) and 18 (Godefroit, Bolotsky & Bolotsky, 2012), respectively, for the cervicals; 16 and 20, respectively, for the dorsals (Horner, Weishampel & Forster, 2004); and 8 (Prieto-Márquez, 2008) and 12 (Horner, Weishampel & Forster, 2004), respectively, for the sacrals. Given that the tail in hadrosaurids accounts for approximately 40% of the body length (e.g., *Brachylophosaurus canadensis* MOR 794; *Corythosaurus casuarius* AMNH 5338; *Gryposaurus notabilis* ROM 764), we added this percentage to our length estimate. Finally, following Taylor & Wedel (2013) we added 11% to the overall length estimate to account for the presence of intervertebral cartilage.

We adhere to the recent phylogenetic hypothesis of hadrosauroid relationships presented by Prieto-Márquez, Erickson & Ebersole (2016; fig. 2) and, at a more inclusive level, to the phylogenetic framework of the Iguanodontia proposed by McDonald (2012). The definition of the Hadrosauridae adopted here is that of Prieto-Márquez (2010a), i.e., the last common ancestor of *Hadrosaurus foulkii* Leidy, 1858, *Saurolophus osborni* Brown, 1912, and *Lambeosaurus lambei* Parks, 1923, and all its descendants. We follow Sereno’s (2005) definitions of the Hadrosauroidea and the Iguanodontia. Accordingly, the Hadrosauroidea is the most inclusive clade containing *Parasaurolophus walkeri* Parks, 1922; but not *Iguanodon bernissartensis* Boulenger in Beneden, 1881. The Iguanodontia is the most inclusive clade containing *Parasaurolophus walkeri* Parks, 1922; but not *Hypsilophodon foxii* Huxley, 1869 and *Thescelosaurus neglectus* Gilmore, 1913.

## Geological Setting

**Figure 1:**
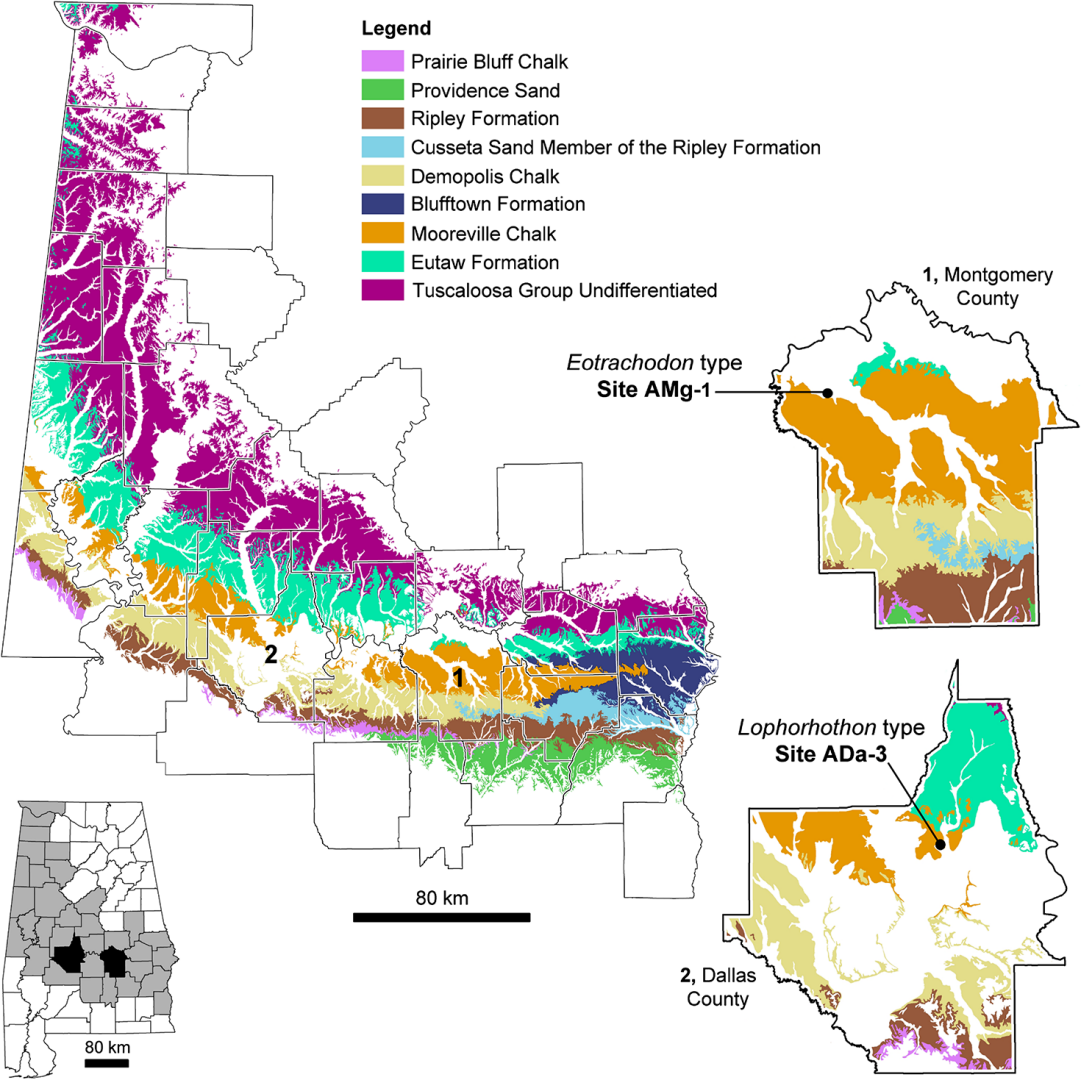
Distribution of hadrosauroid type-specimens from Alabama (southeastern USA) with Upper Cretaceous surface geology and the approximate locations of fossil localities ADa-1 and AMg-1, where the holotypes of *Lophorhothon atopus* and *Eotrachodon orientalis*, respectively, were collected.

**Figure 2:**
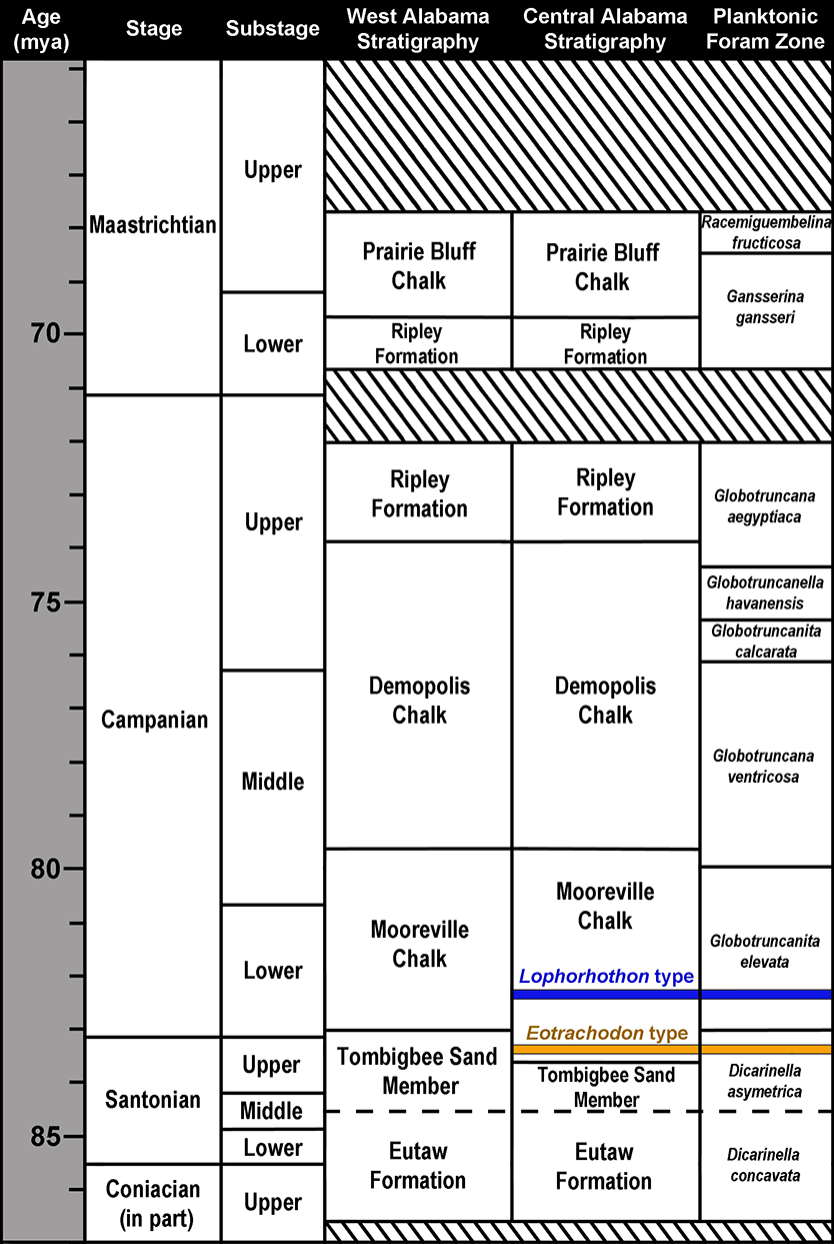
Upper Coniacian to Maastrichtian surface stratigraphy in Alabama and microfossil biostratigraphy. The approximate stratigraphic position of the holotypes of *Lophorhothon atopus* (FMNH P27383) and *Eotrachodon orientalis* (MSC 7949) are indicated. Striped areas represent unconformities. Biostratigraphic zones based on Caron (1985), Perch-Nielsen (1985) and Puckett (2005).

The exposed Upper Cretaceous units in Alabama form a nearly time-continuous series ranging from the Cenomanian to the Cretaceous-Paleogene boundary (Kiernan, 2002; Ebersole & Dean, 2013). These units are exposed in 34 counties in Alabama and create an arc that spans across the center of the state and up towards its northwest corner (Fig. 1). MSC 7949 was discovered at locality AMg-1, a creek site located within the city limits of Montgomery in Montgomery County (more specific locality information for MSC 7949 is on file at McWane Science Center in Birmingham, Alabama). MSC 7949 was excavated in situ from the basal Mooreville Chalk, less than 24 cm above the conformable contact with the underlying Tombigbee Sand Member of the Eutaw Formation (Fig. 2). In Alabama, the contact between these two units is time-transgressive, ranging from the early Campanian in western Alabama to middle-to-late Santonian in the central part of the state. In the vicinity of locality AMg-1, the contact between these two units falls below the last occurrence of the planktonic foraminifer *Dicarinella asymetrica* Sigal, 1952 (Puckett, 1994; Mancini, Puckett & Tew, 1996; Puckett, 2005; Liu, 2007), the last occurrence of which defines the Santonian-Campanian boundary (Caron, 1985). Additional correlations of the planktonic foraminifers, ostracodes, and nanoplankton suggests the lower 32 m of the Mooreville Chalk in central Alabama falls within the upper Santonian (Puckett, 1994; Mancini, Puckett & Tew, 1996), placing the geologic age of MSC 7949 within this interval (Fig. 2), with an estimated age of 83–84 Ma.

The Mooreville Chalk in Alabama is comprised largely of clays made up of compact fossiliferous chalk and chalky marl. However, the lower 1.5 m of this unit, an unnamed member, consists of compact calcarenite and silt, scattered phosphatic pellets, quartz, glauconite, and abundant macrofossils (both vertebrate and invertebrate) and steinkerns (Raymond et al., 1988; Liu, 2007). This suggests the depositional setting of the Mooreville Chalk represents a calm, middle-shelf environment with dysoxic bottom conditions (Wylie & King, 1986). In Alabama, the Mooreville Chalk has long been known for its rich diversity of marine vertebrates; however this unit has also produced terrestrial forms (see Ebersole & King, 2011; Ikejiri et al., 2013). In addition to hadrosauroids, remains of tyrannosauroids, nodosaurids, and a dromaeosaurid have been found (Langston, 1960; Kiernan & Schwimmer, 2004; Ebersole & King, 2011). To date, all dinosaur material from the southeastern USA has been recovered within marine deposits, indicating that these specimens were fluvially transported prior to deposition (Schwimmer, 1997; Ebersole & King, 2011).

## Osteology of *Eotrachodon Orientalis*

*Eotrachodon orientalis* is known from a single immature specimen housed at MSC. It consists of a well-preserved, nearly complete, partially articulated skull and mandible, and an incomplete postcranium. Cranial elements include both premaxillae, maxillae, jugals, partial right nasal, left lacrimal, left prefrontal, frontals, postorbitals, squamosals, left quadrate, parietal, partial braincase (supraoccipital, left opisthotic-exoccipital complex, prootics, basisphenoid, laterosphenoids, and parasphenoid), predentary, dentaries, surangulars, right angular, partial right hyoid, and various maxillary and dentary teeth. The postcraniumn is represented by the axis and several cervical, dorsal, sacral, and caudal vertebrae, partial left pubis, partial left ischium, partial right tibia, and left manual phalanges III-2 and V-2.

**Figure 3:**
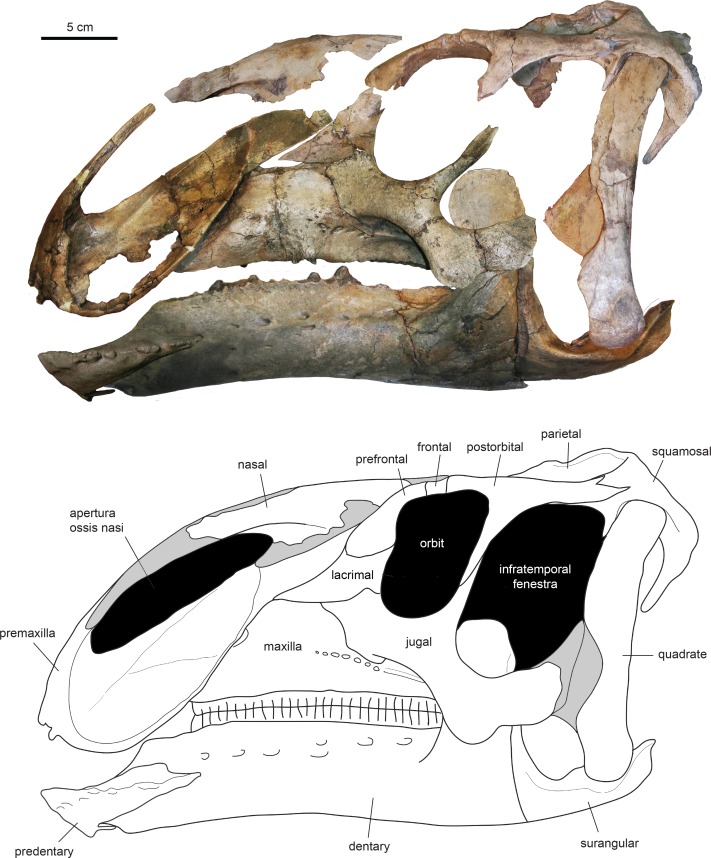
Assembled holotype skull of *Eotrachodon orientalis*, MSC 7949, in left lateral view. The mandible and nasal bone have been reversed to match the orientation of the other elements. Dark grey areas in the line drawing represent missing bones. Mount of the skull and mandible from Prieto-Márquez, Erickson & Ebersole (2016; figs. 1A and 1B), reprinted by permission of the Society of Vertebrate Paleontology, www.vertpaleo.org.

The type specimen of *Eotrachodon orientalis* shows a relatively deep skull (Fig. 3), with a maximum depth along the quadrate/maximum length ratio of 0.54. The pre-orbital region of the skull accounts for 45% of the length of the skull. The dorsal surface of the rostrum is prominently curved rostroventrally. The orbit is elliptical, deeper than wide, with the long axis of the ellipse caudodorsally directed. The infratemporal fenestra is deeper and broader than the orbit, as in the hadrosauroid *Tethyshadros insularis* (Dalla Vecchia, 2009) and kritosaurin hadrosaurids (Prieto-Márquez, 2014). The supratemporal fenestra is subrectangular and its long axis is rostrocaudally oriented.

### Facial skeleton

#### Premaxilla

**Figure 4:**
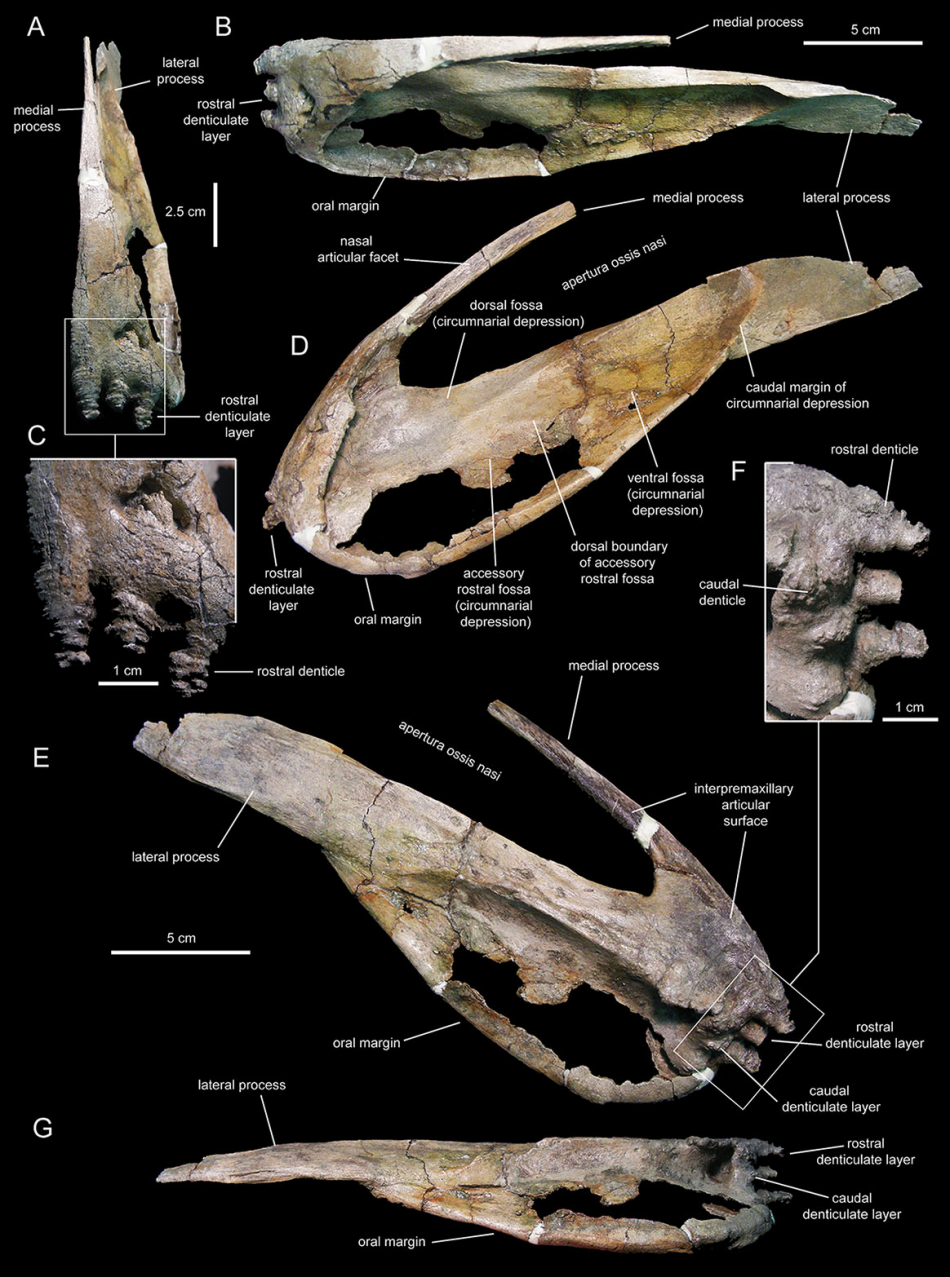
Left premaxilla of *Eotrachodon orientalis* (holotype MSC 7949). (A) Rostral view. (B) Dorsal view. (C) Rostral view of rostral layer of premaxillary denticles. (D) Lateral view. (E) Medioventral view. (F) Medioventral view of premaxillary denticles. (G) Ventral view.

**Figure 5:**
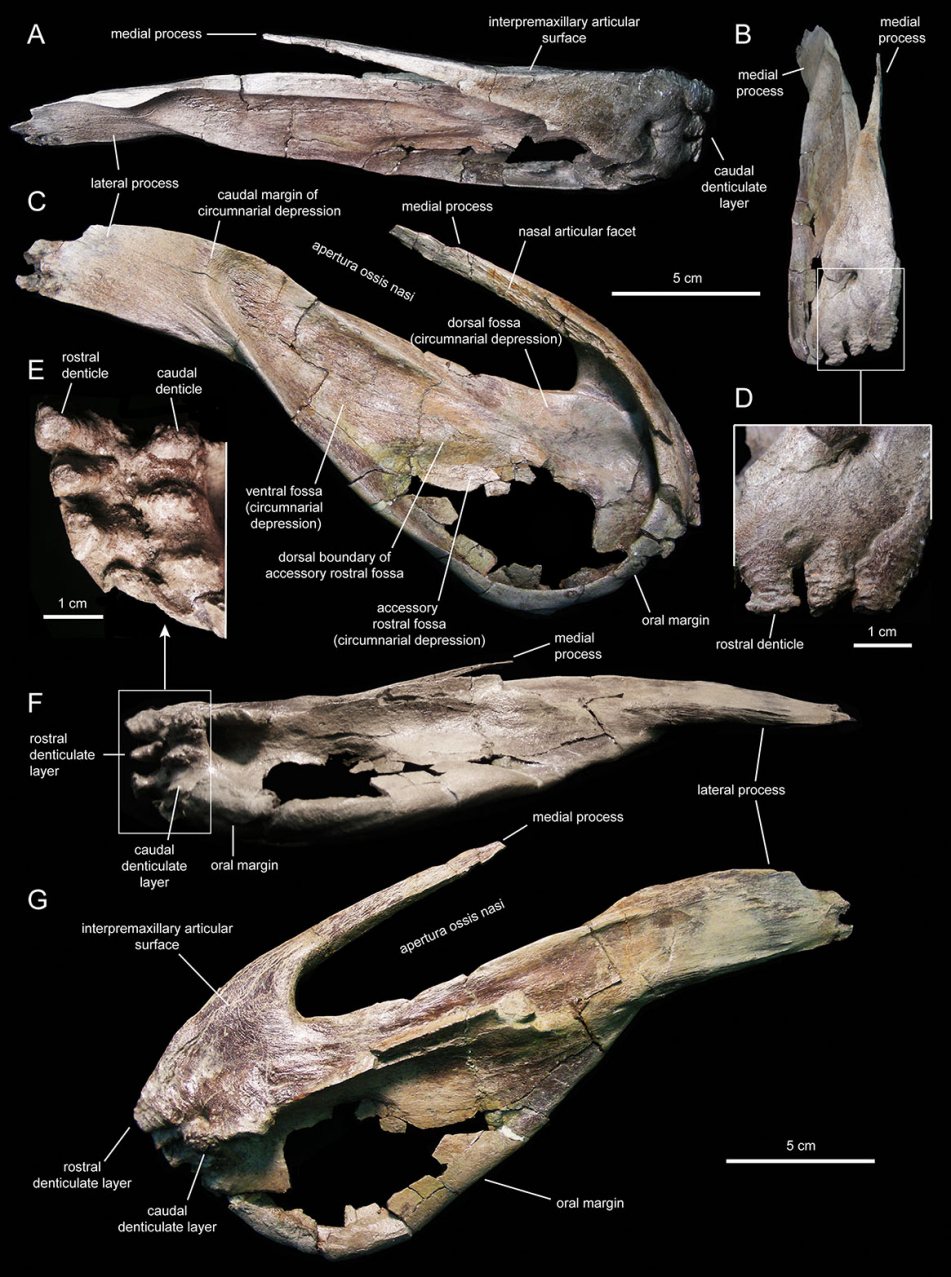
Right premaxilla of *Eotrachodon orientalis* (holotype MSC 7949). (A) Dorsal view. (B) Rostral view. (C) Lateral view. (D) Rostral view of rostral layer of premaxillary denticles. (E) Ventral view of premaxillary denticles. (F) Ventral view. (G) Medioventral view.

The premaxilla (Figs. 4 and 5; Table 1) forms the upper ‘duck-bill’ of hadrosauroid dinosaurs. The oral margin of the premaxilla of *Eotrachodon orientalis* is widely arcuate, as well as thin and reflected (Figs. 4D and 5C) as in saurolophin (Bell, 2011a) and kritosaurin (Prieto-Márquez, 2014) saurolophines. This reflected oral margin gradually becomes thinner distally along the lateral margin of the premaxilla. The premaxillary oral margin is moderately offset ventrally relative to the occlusal plane of the maxilla (Fig. 3), so that the distance between these premaxillary and maxillary areas is half of the mean depth of the dentary ramus. This offset is greater in saurolophid hadrosaurids, in which the distance between the oral margin of the premaxilla and the occlusal plane of the maxillary tooth row is greater than the mean depth of the dentary ramus (Prieto-Márquez, 2010a).

**Table 1:** Selected facial measurements (in mm) of the holotype specimen (MSC 7949) of *Eotrachodon orientalis*.

Element	Measurement
Premaxilla (left), maximum length along lateral process	258
Premaxilla (left), maximum width from medial to lateral process	94
Premaxilla (right), maximum length along lateral process	237
Premaxilla (right), maximum width from medial to lateral process	88
Maxilla (left), maximum length	220
Maxilla (left), height from alveolar margin to highest point of dorsal process (incomplete)	87
Maxilla (right), maximum length	223
Maxilla (right), height from alveolar margin to highest point of dorsal process	93
Nasal (fragment), maximum length	54
Nasal (fragment), maximum width perpendicular to length	103
Lacrimal, maximum height along caudal margin including dorsal flange	73
Prefrontal, maximum length	74
Prefrontal, maximum width of the rostroventral flange	33
Jugal (left), length from apex of rostral process to caudal margin of quadratojugal flange	156
Jugal (left), height along the distal tip of the postorbital ramus perpendicular to the ventral margin	98
Jugal (right), length from apex of rostral process to caudal margin of quadratojugal flange	165
Jugal (right), height along the distal tip of the postorbital ramus perpendicular to the ventral margin	94
Postorbital (left), length from rostral end to distal tip of caudal ramus	100
Postorbital (right), length from rostral end to distal tip of caudal ramus	99
Quadrate, maximum length	193
Squamosal (left), length from caudal margin of postcotyloid process to rostral tip of postorbital process	89
Squamosal (right), length from caudal margin of postcotyloid process to rostral tip of postorbital process	86

Two rows, each containing three large denticles are present: one bordering external to the oral margin and the second set caudally on the ventral surface of the premaxilla (Figs. 4F and 5E). The external row consists of three sub-conical denticles that are rostroventrally directed. The external surfaces of these denticles show transverse grooves (Fig. 4C). The caudal denticles are mediolaterally compressed, ventrally directed, and larger than the rostral denticles.

Most of the lateral surface of the premaxilla is excavated by an extensive elliptic circumnarial depression (Figs. 4D and 5C). As in all saurolophine hadrosaurids (Prieto-Márquez & Wagner, 2014), this excavation entirely surrounds the bony naris and is compartmentalized into subsidiary fossae. Specifically, the circumnarial depression of *Eotrachodon orientalis* consists of three fossae, as in species of *Edmontosaurus* Lambe, 1917 (Campione & Evans, 2011). Two thin ridges converge rostroventrally to divide the circumnarial depression of *E. orientalis* into dorsal, ventral, and accessory rostral fossae (Figs. 4D and 5C). There is no premaxillary foramen within the circumnarial depression of *E. orientalis*, although the area adjacent to the rostral margin of the apertura ossis nasi is deeply excavated. In saurolophines, a large foramen is present near that area of the premaxilla, rostroventral to the rostral margin of the apertura ossis nasi (e.g., *Brachylophosaurus canadensis* Sternberg, 1953 [CMN 8893], or *Edmontosaurus annectens* (Marsh, 1892, SM R4036). *E. orientalis* displays a large foramen on the rostral surface of the premaxilla dorsal to the external denticles (Fig. 4C), as in the basal hadrosauroid *Bactrosaurus johnsoni* Gilmore, 1933 (Prieto-Márquez, 2011a) and some saurolophine hadrosaurids like *B. canadensis* (see Prieto-Márquez, 2005) and *Maiasaura peeblesorum* Horner & Makela, 1979 (Horner, 1983).

The medial process of the premaxilla is a thin rod-like structure that is rostrodorsally continuous with the rostromedial margin of the circumnarial depression (Figs. 4 and 5). The tapering distal end is missing in both premaxillae. The medial process contributes to form the dorsal margin of the apertura ossis nasi. The lateral surface of the process is occupied by a flat articular facet for reception of the rostral process of the nasal that would extend rostrally to the level of the rostrodorsal corner of the apertura ossis nasi.

The lateral process of the premaxilla is a wedge-shaped blade that extends caudodorsally, adjacent to the caudoventral end of the circumnarial depression and the caudal end of the lateral margin of the main body of the premaxilla (Figs. 4 and 5). Proximally, the lateral process is abruptly deflected ventrally, forming a 165° angle with the long axis of circumnarial depression.

Most of the medial side of the main body of the premaxilla, including the medial process, is occupied by an extensive and flat interpremaxillary articular surface (Figs. 4E and 5G). This surface is separated from the depressed ventral side of the premaxilla by a sharp and prominent ridge. The premaxilla gradually becomes narrower caudally until reaching the lateral process at its narrowest point, which corresponds to the post-oral constriction of the skull.

#### Maxilla

**Figure 6:**
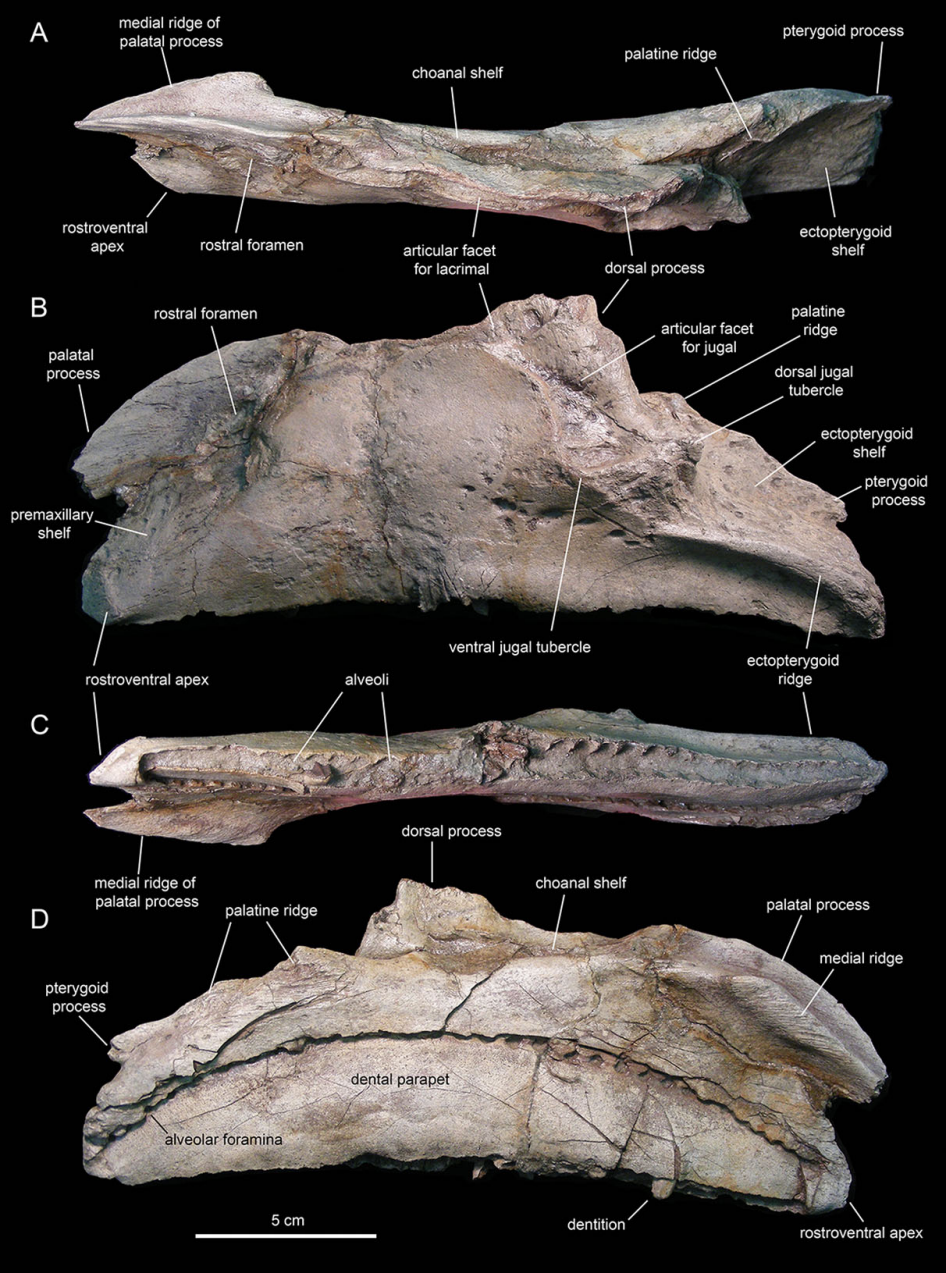
Left maxilla of *Eotrachodon orientalis* (holotype MSC 7949). (A) Dorsal view. (B) Lateral view. (C) Ventral view. (D) Medial view.

**Figure 7:**
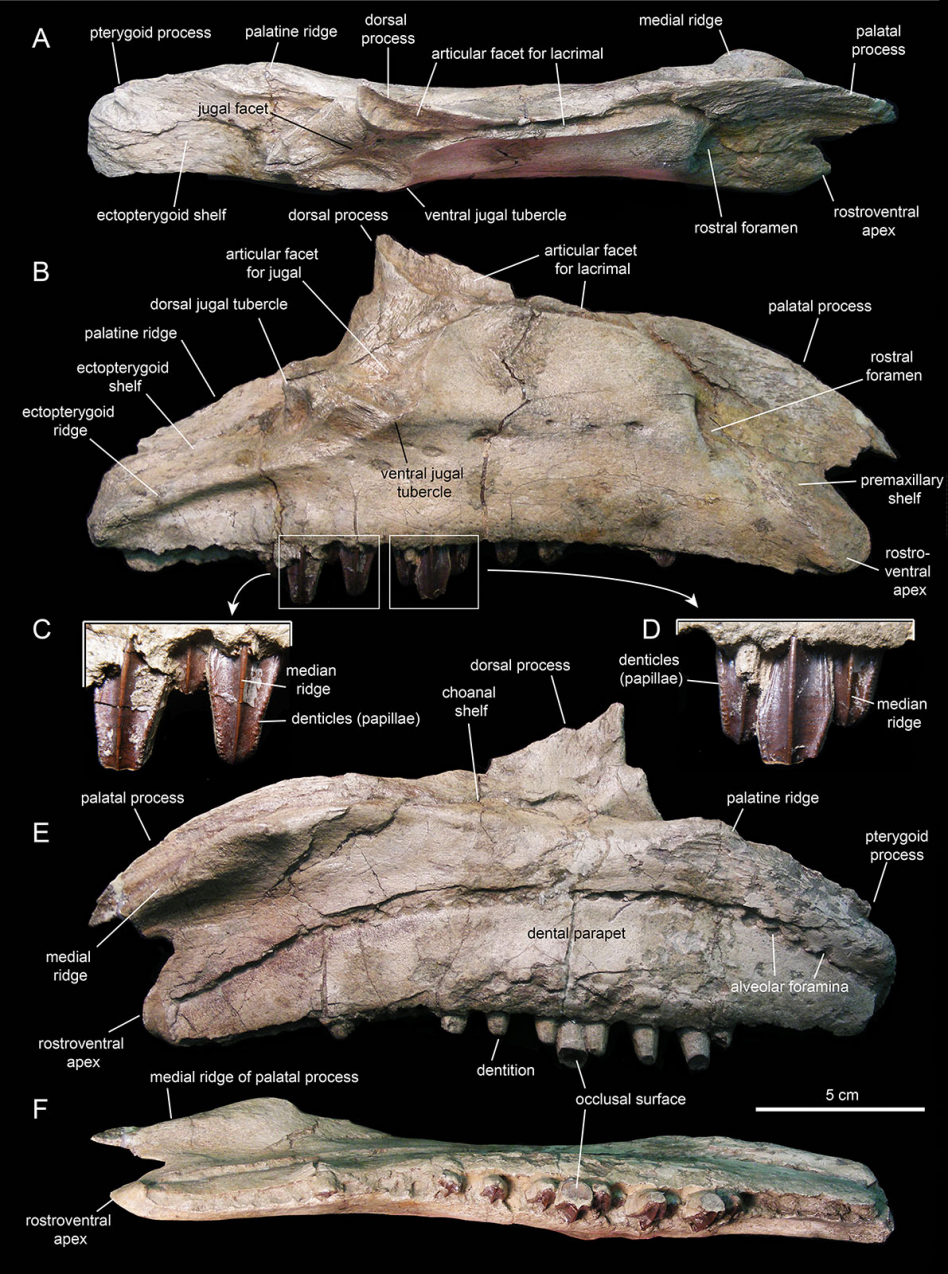
Right maxilla of *Eotrachodon orientalis* (holotype MSC 7949). (A) Dorsal view. (B) Lateral view. (C and D) Labial views of maxillary tooth crowns. (E) Medial view. (F) Ventral view. The lateral view of the maxilla is from Prieto-Márquez, Erickson & Ebersole (2016; fig. 1C), reprinted by permission of the Society of Vertebrate Paleontology, www.vertpaleo.org.

The maxilla (Figs. 6 and 7; Table 1) is an elongate and mediolaterally compressed element. The rostral half of the maxilla is about twice as deep as the caudal third. The palatal process is a deep flange that extends mediodorsally and curves rostroventrally, forming the rostrodorsal margin of the maxilla (Figs. 6B and 7B). Its medial surface bears a prominent ridge that extends throughout the length of the process and rises dorsomedially (Figs. 6C, 6D, 7E and 7F). This ridge is most prominent at its mid-length. The area comprised in between the medial surface of the palatal process and the dorsal surface of this ridge support the rostral extent of the vomer. Laterally, the base of the palatal process is continuous ventrally with the premaxillary shelf, a smooth and narrow surface that steeply slopes rostroventrally and lateroventrally and supports the caudal region of the main body of the premaxilla (Fig. 7B). Rostroventrally, the premaxillary shelf ends in a broad and blunt apex (the rostroventral process of authors; e.g., Prieto-Márquez et al., 2013). Lateroventral to the palatal process and the premaxillary shelf lays the large rostral foramen (Fig. 7B). This foramen opens rostrolaterally and is found at about mid-depth of the rostral region of the maxilla. This position is below that typically seen in hadrosaurids, where the foramen is located closer to the rostrodorsal margin of the maxilla (Prieto-Márquez, 2010a).

The maxilla of *Eotrachodon orientalis* displays a steep premaxillary shelf. This shelf is angled at 45° with respect to the rostral region of the tooth row. The laterodorsal margin of the maxilla is sub-trapezoidal in lateral view and extensively exposed under the lacrimal (Figs. 6B and 7B). This sub-trapezoidal surface is dorsally bordered by a narrow groove where it articulated with the ventral margin of the lacrimal. The groove is rostrally continuous with the premaxillary shelf. Caudally, it extends onto the dorsal region of the dorsal process of the maxilla, where it becomes an elongate and recessed articular facet for the lacrimal (Fig. 7B). Both the steep premaxillary shelf and extensive laterodorsal margin are attributes shared with the kritosaurins *Gryposaurus* spp. (see Lambe, 1914; Gates & Sampson, 2007) and *Kritosaurus horneri* (see Prieto-Márquez, 2014).

The dorsal process is located caudal to the mid-length of the maxilla (Figs. 6B and 7B). This process exhibits a caudally-skewed lateral profile, being twice as broad as it is tall. The dorsal half of the articular surface for the jugal extends onto the lateral surface of the dorsal process, below the articular facet for the lacrimal (Fig. 6B). The articular facet for the jugal is sub-triangular and is oriented laterally, as typically occurs in hadrosaurids (Prieto-Márquez & Wagner, 2009). Yet, this articular surface shares a prominent and caudally projected dorsal tubercle that ends in a sharp apex (Fig. 7B) with basal hadrosauroids (e.g., *Levnesovia transoxiana* Sues & Averianov, 2009).

At the caudal end of the maxilla, the ectopterygoid shelf only accounts for 25% of the total length of the bone, as in *Eolambia caroljonesa* Kirkland, 1998 (see also McDonald et al., 2012). The shelf is caudoventrally tilted to form a 20° angle with the caudal segment of the tooth row, as in *Levnesovia transoxiana* (see Sues & Averianov, 2009). As in other hadrosaurids, this shelf is also steeply inclined lateroventrally and bordered laterally by a thick ridge that gradually becomes broader caudally (Fig. 6B). Mediodorsally, the ectopterygoid shelf is continuous with the palatine ridge (Figs. 6A and 6B). This ridge raises mediodorsally and displays an irregular scalloped dorsal margin for reception of the ventral margin of the palatine. A partially preserved finger-shaped pterygoid process projects caudally from the caudal extent of the palatine ridge (Fig. 6D).

There are eight and seven foramina piercing the lateral surfaces of the left and right maxilla, respectively. On the left maxilla, the foramina form a rostrodorsally-oriented row ventral to the jugal facet, as in hadrosaurids (Prieto-Márquez, 2010a; Fig. 6B). However, on the right maxilla, the foramina form a long row that extends throughout most of the lateral surface of the bone (Fig. 7B), as in basal hadrosauroids like *Bactrosaurus johnsoni* (see Prieto-Márquez, 2011a; Fig. 7B) and *Equijubus normani* You et al., 2003, and basal iguanodontians like *Mantellisaurus atherfieldensis* Hooley, 1925 (e.g. NHM R11521).

The medial surface of the maxilla is relatively flat (Figs. 6D and 7E). The arcuate row of alveolar foramina is positioned dorsal to the mid-depth of the maxilla, bounding dorsally the bony parapet that medially encloses the maxillary dental battery. The latter bears 32 tooth positions, a count that is within the minimum range recorded in the Hadrosauridae (Prieto-Márquez, 2010a). There is only one functional tooth exposed on the occlusal plane (Fig. 7F), as in basal iguanodontians (Norman, 2004).

#### Nasal

**Figure 8:**
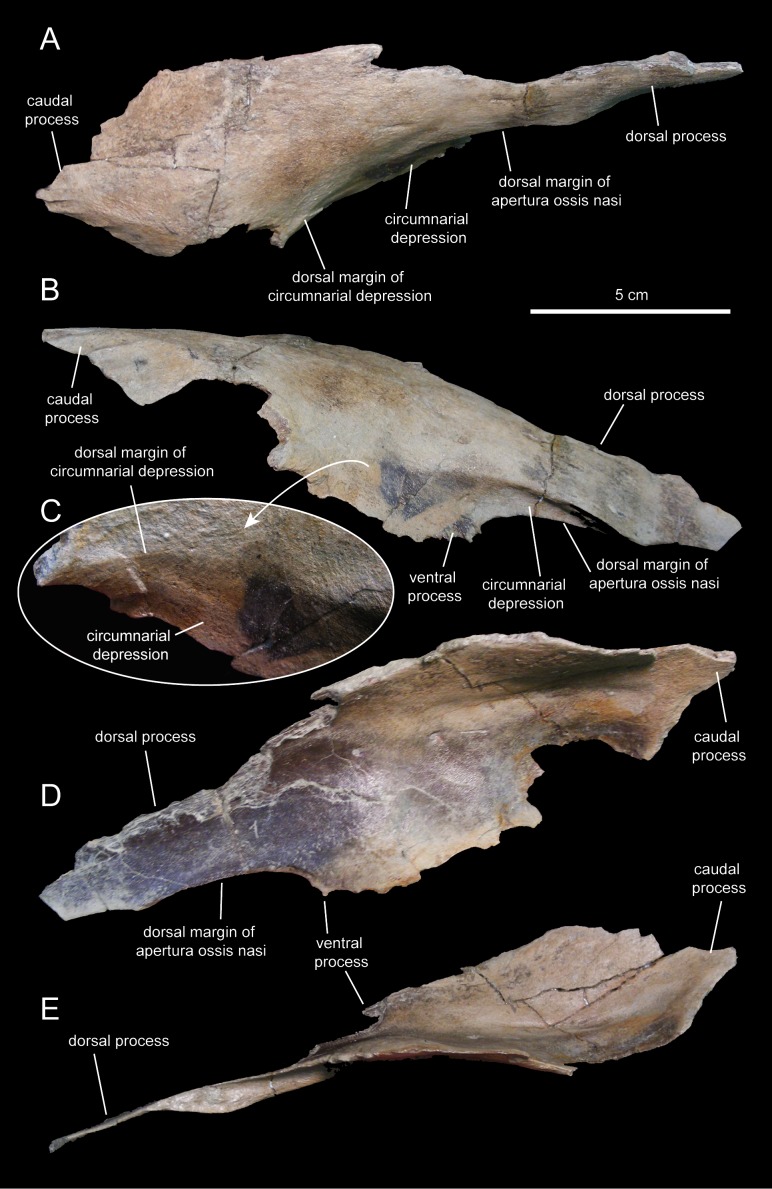
Partial right nasal of *Eotrachodon orientalis* (holotype MSC 7949). (A) Dorsal view. (B) Lateral view. (C) Lateral view of the caudal region of the circumnarial depression. (D) Medial view. (E) Ventral view. The lateral view of the nasal is from Prieto-Márquez, Erickson & Ebersole (2016; fig. 1F), reprinted by permission of the Society of Vertebrate Paleontology, www.vertpaleo.org.

The nasal (Figs. 3 and 8; Table 1) consists of a thin plate that curves lateroventrally to form the caudodorsal region of the rostrum and the caudodorsal margin of the apertura ossis nasi. The only available nasal is missing most of its medial margin, part of the caudolateral wall of its main body, most of the ventral process, and the distal segment of the dorsal process (Fig. 8). Unlike *Lophorhothon atopus* (Langston, 1960), the dorsal surface of the main body of the nasal of *Eotrachodon orientalis* gently curves caudodorsally showing no elevation that would indicate the presence of a cranial crest. A wedge-shaped caudal process exists along the caudolateral margin of the nasal (Figs. 8A and 8B) that would probably insert in between the rostrolateral and medial margins of the frontal and prefrontal, respectively.

The ventral border of the dorsal process of the nasal forms the caudodorsal margin of the apertura ossis nasi. Dorsally and adjacent to this margin, the nasal is excavated by the circumnarial depression. In this area, the circumnarial depression is deeply incised ventral to the proximal region of the dorsal nasal process. Caudally, however, the depression becomes gradually shallower above the proximal region of the ventral process of the nasal (Figs. 8B and 8C).

What remains of the dorsal process of the nasal is a mediolaterally compressed wedge-shaped structure that projects rostroventrally (Figs. 8B and 8D). This process overlapped the entire lateral surface of the medial process of the premaxilla, while both elements formed the dorsal margin of the apertura ossis nasi. As evidenced by the length of the articular facet for the nasal on the medial process of the premaxilla, the dorsal process of the nasal extended to above the rostral margin of the apertura ossis nasi.

#### Prefrontal

**Figure 9:**
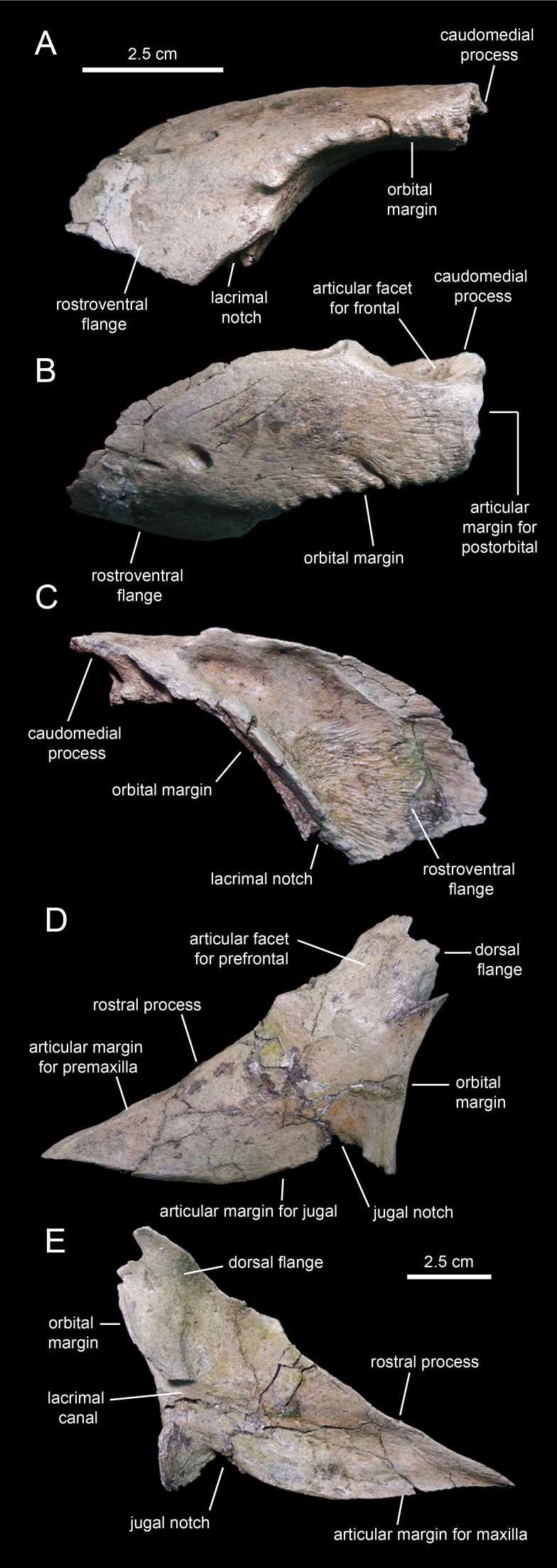
Facial elements of *Eotrachodon orientalis* (holotype MSC 7949). (A–C) Left prefrontal in lateral, dorsal, and medial view, respectively. (D and E) Left lacrimal in lateral and medial view, respectively.

The prefrontal (Fig. 3; Table 1) forms the rostrodorsal margin of the orbit. The prefrontal of *Eotrachodon orientalis* is nearly completely preserved, missing the distal regions of the rostroventral flange and the caudomedial process (Figs. 9A–9C). The bone is crescent-shaped in lateral view. Caudally, the prefrontal is dorsoventrally compressed; however, as it curves ventrally, the prefrontal becomes mediolaterally compressed while expanding dorsoventrally to form a thin rostroventral flange (Figs. 9A and 9C). This flange overlaps the lateral surface of the dorsal flange of the lacrimal. A deep triangular notch, for reception of the lacrimal (Fig. 9A), occurs at the caudoventral corner of the flange.

The orbital margin of the prefrontal is carved with a series of short deep indentations that follow the rostrodorsal corner of the orbit and end abruptly near the proximal region of the rostroventral flange. The caudomedial region of the prefrontal is recessed and projected into a caudomedial process (Fig. 9B) that inserts into the rostrolateral margin of the frontal.

A foramen is present on the laterodorsal surface of the prefrontal deepening caudolaterally (Fig. 9B). In contrast to the dorsoventrally convex lateral surface of the prefrontal, most of the medial side is deeply excavated, particularly throughout its caudodorsal extent (Fig. 9C). The ventral surface of the prefrontal is smooth and forms the roof of the rostral region of the orbit. The prefrontal is excluded from the circumnarial depression.

#### Lacrimal

As in non-lambeosaurine hadrosauroids, the lacrimal (Figs. 9D and 9E; Table 1) of *Eotrachodon orientalis* is a triangular rostrally elongate plate. The wedge-shaped rostral process forms about two-thirds of the lacrimal. Its medial surface is concave, particularly along the lacrimal canal that extends longitudinally at mid-depth of the rostral process (Fig. 9E). Both the rostrodorsal and ventral margins of the process show an extremely narrow ledge for articulation with the lateral process of the premaxilla, and the dorsal process of the maxilla and rostral process of the jugal, respectively. The ventral margin of the rostral process of the lacrimal displays a convex lateral profile rostral to the jugal notch (Fig. 9D), a condition also present in the saurolophines *Gryposaurus* spp. (Gates & Sampson, 2007), *Kritosaurus horneri* Hunt & Lucas, 1993 (Prieto-Márquez, 2014), *Saurolophus* spp. (Brown, 1912; Bell, 2011a; Bell, 2011b), and *Prosaurolophus maximus* Brown, 1916 (McGarrity, Campione & Evans, 2013). The jugal notch shows a triangular lateral profile and ventrally receives the caudodorsal region of the rostral process of the jugal (Fig. 9D). The notch is bounded caudally by the tetrahedral caudoventral corner of the lacrimal (Fig. 9E). The caudal margin of the lacrimal contributes to the rostroventral margin of the orbit. This margin is gently concave and represents the widest region of the lacrimal. It is pierced by the opening of the lacrimal canal (Fig. 9E).

The caudodorsal region of the lacrimal consists of a medially deflected flange that extends dorsally to underlie the rostroventral flange of the prefrontal (Fig. 9D). The caudodorsal margin of this flange of the lacrimal is coarsely scalloped.

#### Jugal

**Figure 10:**
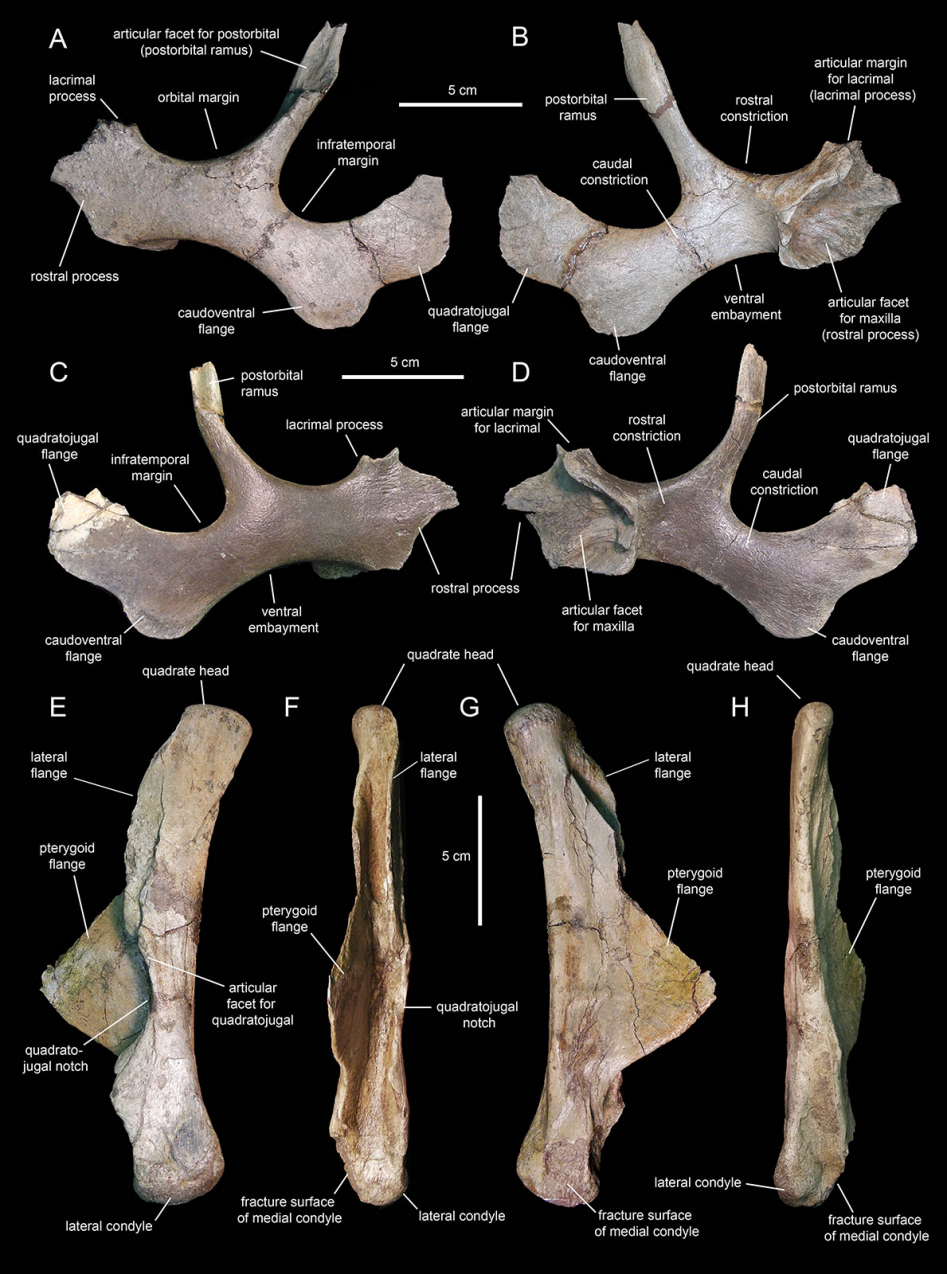
Facial elements of *Eotrachodon orientalis* (holotype MSC 7949). (A and B) Left jugal in lateral and medial view, respectively. (C and D) Right jugal in lateral and medial view, respectively. (E–H) Quadrate in lateral, rostral, medial, and caudal view, respectively. The lateral view of the right jugal is from Prieto-Márquez, Erickson & Ebersole (2016; fig. 1E), reprinted by permission of the Society of Vertebrate Paleontology, www.vertpaleo.org.

The jugals (Figs. 10A–10D; Table 1) are nearly completely preserved, except for the distal ends of the rostral processes, postorbital rami, and quadratojugal flanges. Rostrally, the jugal is dorsoventrally expanded to form a triangular rostral process for articulation with the maxilla and lacrimal. The elongate apex of this process lays within its dorsal half, as in non-brachylophosaurin saurolophines (Gates et al., 2011; Fig. 10A). The caudodorsal margin of the rostral process forms a notched and relatively shallow lacrimal process (Fig. 10C) that minorly contributes to the rostroventral margin of the orbit. The caudoventral margin of the rostral process is also shallow and forms a broad flange that is medially recessed from the lateral surface and ventral margin of the rostral process (Figs. 10C and 10D). This flange represents the ventral extent of the medial articular surface of the process. The medial articular facet of the rostral process is roofed by a narrow shelf. This shelf extends medially from the orbital margin (Figs. 10B and 10D), a condition shared with basal hadrosauroids like *Bactrosaurus johnsoni* (see Prieto-Márquez, 2011b). However, unlike in basal hadrosauroids (e.g., *Tanius sinensis* Wiman, 1929), with the exception of *Lophorhothon atopus* Langston, 1960, the shelf of *Eotrachodon orientalis* is rostrodorsally rather than rostrally oriented (Figs. 10B and 10D). Caudally, the dorsal and ventral margins of the rostral process are continuous with the orbital margin and an extensive embayment that forms most of the ventral border of the jugal, respectively.

The postorbital ramus rises dorsally and slightly caudally from about mid-length of the dorsal margin of the jugal (Fig. 10A). Dorsally, this ramus is rostrally excavated for reception of the jugal ramus of the postorbital. The postorbital ramus separates the ventral, and part of the caudal, margins of the orbit from the ventral, and part of the rostral, margins of the infratemporal fenestra. The caudal constriction that underlies the infratemporal fenestra is 85% as deep as the rostral constriction that lies beneath the orbit. The orbital and infratemporal margins are subequally wide.

The caudoventral flange is greatly expanded, being 1.6 times deeper than the caudal constriction at its narrowest point. The quadratojugal flange forms the caudodorsal region of the jugal and displays an auricular lateral profile (Fig. 10C). There is a relatively wide and prominent concavity between the caudoventral and quadratojugal flanges.

#### Quadrate

The quadrate (Figs. 10E–10H; Table 1) is a columnar element linking the skull to the mandible at the caudal end of the skull. This bone is slightly curved caudally; specifically, the rostral margin of the dorsal segment of the quadrate forms an angle of 167° with the long axis of the quadratojugal notch (Fig. 10E). The proximal articular surface of the quadrate head is convex and mediolaterally compressed. Its caudal margin lacks a squamosal buttress.

The medial and lateral surfaces of the quadrate shaft extend rostrally to form two flanges (Fig. 10F). The delicate wing-like pterygoid flange extends rostromedially and is missing most of its rostrodorsal margin (Fig. 10G). Proximally, its medial surface is deeply depressed for reception of the caudodorsal flange and the caudoventral process of the pterygoid. The lateral flange of the quadrate is limited to the dorsal segment of the bone and only extends a short distance beyond the rostrocaudal width of the shaft (Fig. 10E).

The quadratojugal notch shows a wide arcuate lateral profile (Fig. 10E). Its dorsal margin forms a 26° angle with the caudal margin of the quadrate. The mid-point of the notch lays ventral to the mid-length of the quadrate. Both the dorsal and ventral margins of the notch are subequally long and display narrow facets for articulation with the caudal margin of the quadratojugal.

The distal end of the quadrate is slightly expanded and consists of two condyles (Fig. 10F). The substantially smaller medial condyle is eroded away. As in *Telmatosaurus transsylvanicus* Nopcsa, 1900 (e.g., NHM R3386), *Lophorothon atopus* (e.g., FMNH P27383), and hadrosaurids (Prieto-Márquez, 2010a), the ventral articular surface of the quadrate that contacts the surangular is subtriangular in ventral view due to the rostrocaudal expansion of the lateral condyle.

#### Squamosal

**Figure 11:**
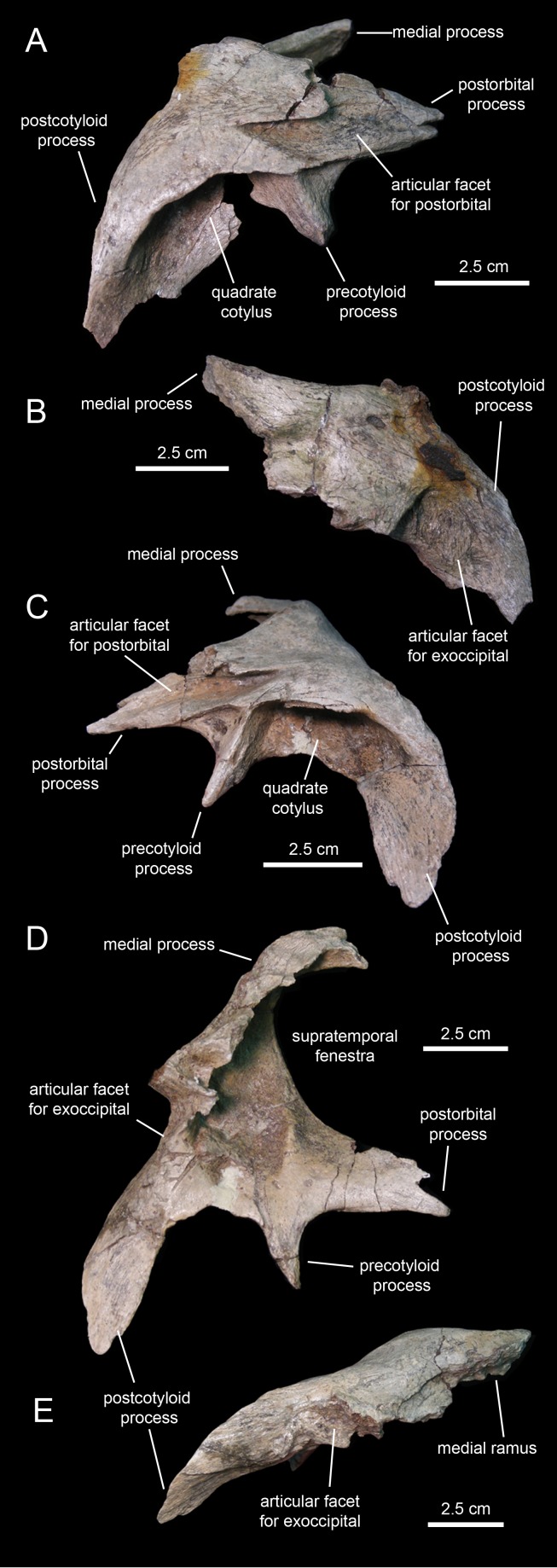
Squamosals of *Eotrachodon orientalis* (holotype MSC 7949). (A and B) Right squamosal in lateral and caudal view, respectively. (C–E) Left squamosal in lateral, ventromedial, and caudal view, respectively.

The squamosal (Fig. 11; Table 1) forms the caudodorsal corner of the skull. The main body of this element consists of a thick, curved lamina that is deeply concave ventrally. Three processes radiate medially, rostrally, and ventrolaterally from the main body of the squamosal (Fig. 11D). The medial margin of the postorbital process and the rostral margin of the medial process caudally bound the supratemporal fenestra.

The postorbital process is triangular, dorsoventrally compressed, and projects rostrally and slightly ventrally (Fig. 11C). Most of its dorsal surface is occupied by an extensive recessed facet that is overlapped by the caudal ramus of the postorbital, as the postorbital process inserts under the latter. The caudal margin of the articular facet for the postorbital process is Z-shaped for reception of the bifid distal margin of the postorbital caudal ramus (Fig. 11A).

A sub-conical precotyloid process extends lateroventrally from beneath the lateral margin of the postorbital process (Fig. 11C). The precotyloid process is relatively short, being only 60% as long as the quadrate cotylus is wide. A small triangular depression exists in between the rostral margin of the proximal region of the precotyloid process and the lateral edge of the proximal region of the postorbital process. The caudal margin of the precotyloid process rostrally bounds the quadrate cotylus (Fig. 11C). The cotylus consists of a dorsoventrally-compressed but deep oval depression on the lateral surface of the main body of the squamosal that receives the head of the quadrate. The caudal wall of the cotylus is continuous caudally with the postcotyloid process (Figs. 11D and 11E). This process consists of a hook-like lamina that extends lateroventrally from the caudolateral corner of the squamosal. Its recessed caudal surface is rugose and contacts extensively the proximal region of the paroccipital process of the fused opisthotic-exoccipital.

The medial process of the squamosal extends medially and slightly dorsally (Fig. 11B). Distally, this process curves rostrally to form the caudomedial corner of the supratemporal fenestra (Fig. 11D). The two squamosals probably met sagittally, excluding the parietal from the occiput.

#### Postorbital

**Figure 12:**
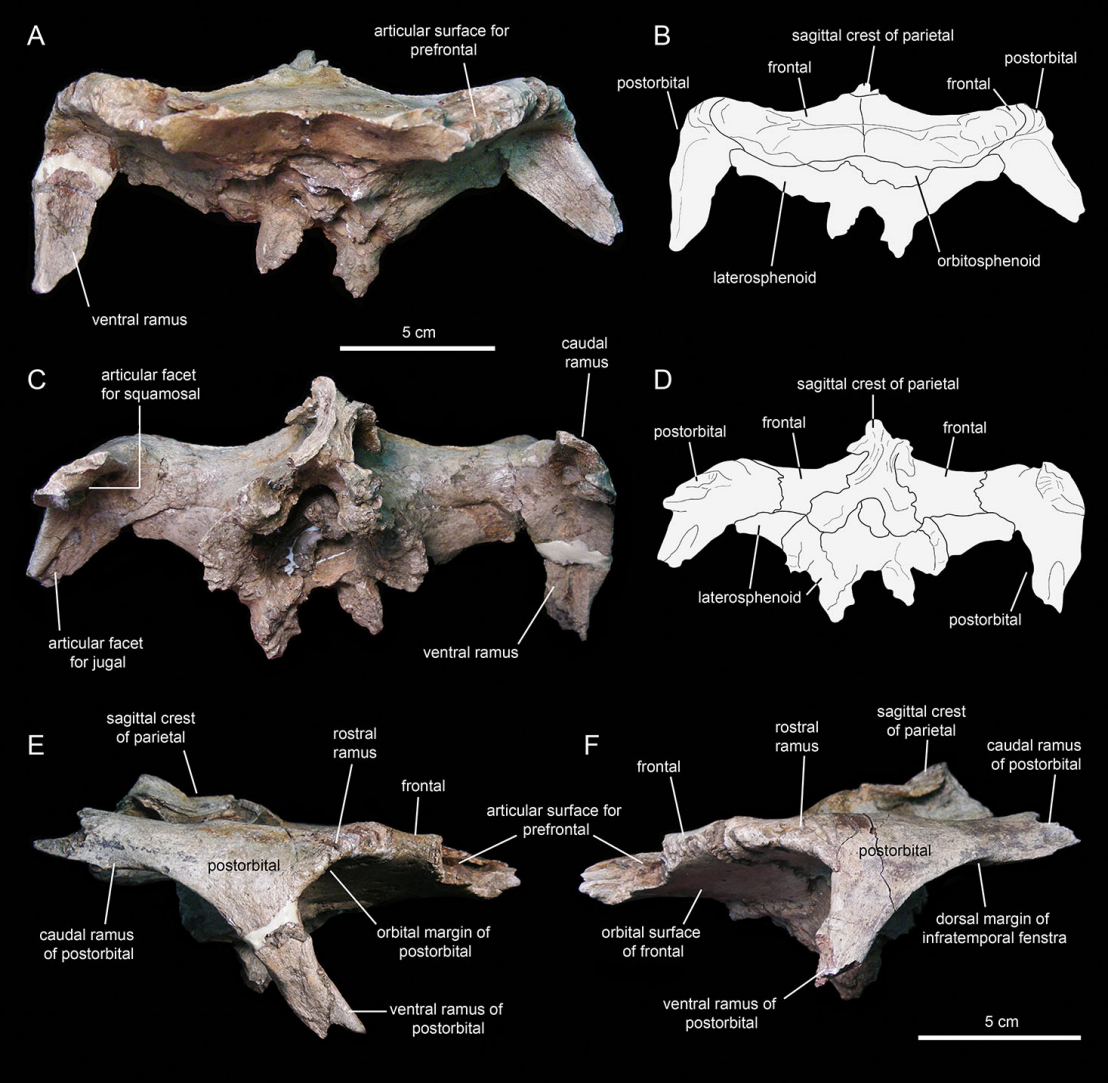
Partial skull roof and braincase of *Eotrachodon orientalis* (holotype MSC 7949). (A) Rostral view. (B) Interpretive line drawing of rostral view. (C) Caudal view. (D) Interpretive line drawing of caudal view. (E) Right lateral view. (F) Left lateral view.

**Figure 13:**
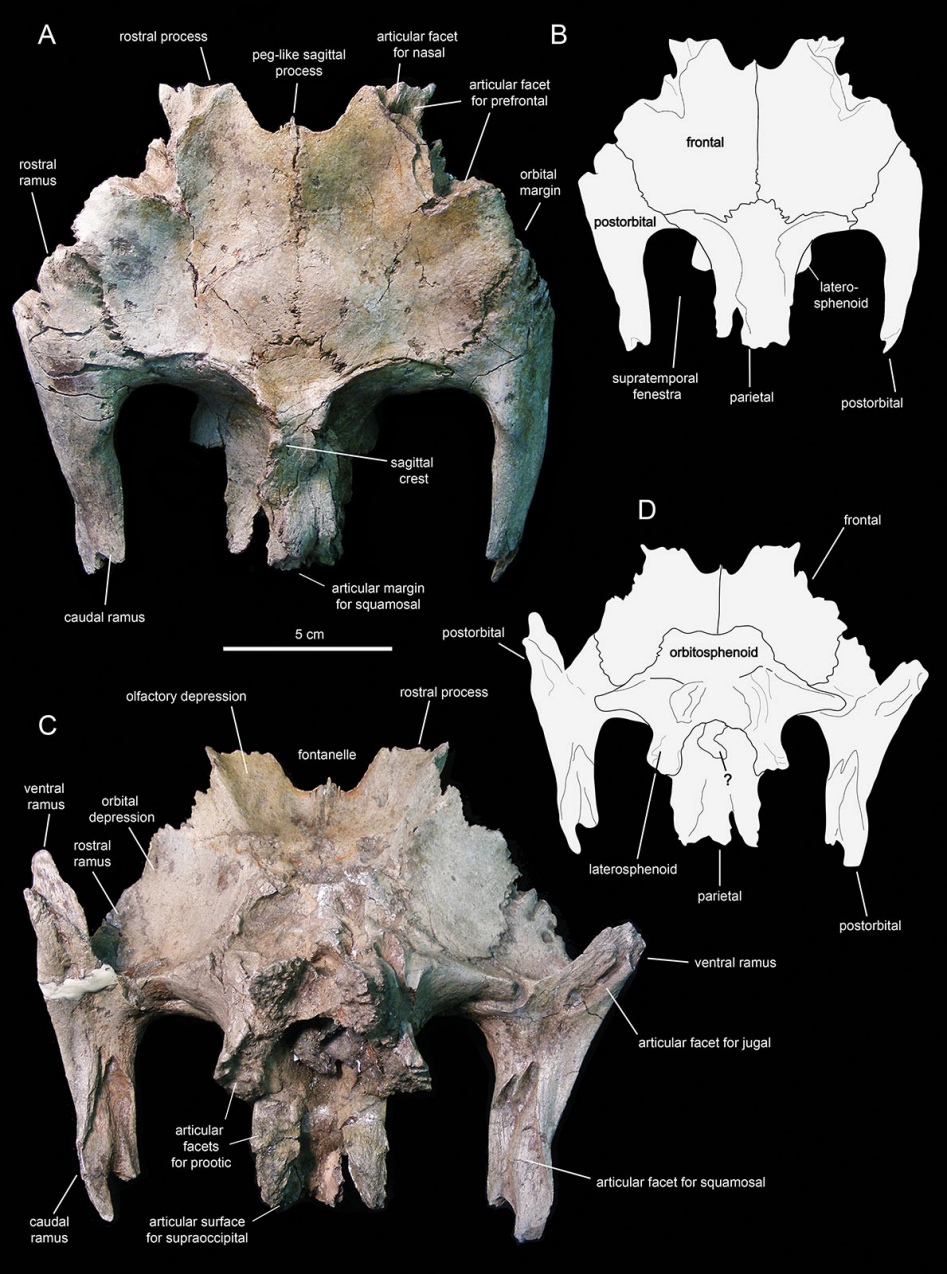
Partial skull roof and braincase of *Eotrachodon orientalis* (holotype MSC 7949). (A) Dorsal view. (B) Interpretive line drawing of dorsal view. (C) Ventral view. (D) Interpretive line drawing of ventral view. The dorsal view is from Prieto-Márquez, Erickson & Ebersole (2016; fig. 1D), reprinted by permission of the Society of Vertebrate Paleontology, www.vertpaleo.org.

The postorbital (Figs. 12E, 12F, 13A and 13C; Table 1) is a triradiate element that forms the caudodorsal margin of the orbit and the rostrodorsal margin of the infratemporal fenestra. Three rami extend rostrally, rostroventrally, and caudally from the central region of the postorbital. Near the proximal region of the squamosal ramus there is a gentle protuberance on the dorsal surface of the central body of the postorbital (Fig. 12E).

The rostral ramus of the postorbital is triangular in dorsal view and shorter and wider than the other two rami (Fig. 13A). Like the prefrontal, the lateral margin of the rostral ramus is ornamented with a series of deep crenulations. Medially, this ramus interdigitates with the frontal throughout most of its length. At its caudal-most extent, however, it articulates with the parietal by means of a small wedge-shaped medial process. The intervening frontal at the orbital margin prevents the rostral ramus of the postorbital from meeting the prefrontal rostrally (Figs. 13A and 13B).

The ventral ramus projects and wedges rostroventrally, forming about half of the caudal and rostral margins of the orbit and the infratemporal fenestra, respectively (Figs. 12E and 12F). The caudal surface of the ventral ramus shows a V-shaped excavation for reception of the postorbital ramus of the jugal (Fig. 13C). In contrast, the rostral surface of the ramus is smooth and concave. The ventromedial side of the central body of the postorbital, dorsal to the ventral ramus, displays a rounded depression that receives the lateral process of the laterosphenoid (Fig. 13C).

The caudal ramus of the postorbital is dorsoventrally compressed. It extends caudally and slightly dorsally to overlap the postorbital process and as much as one-fourth of the quadrate cotylus of the squamosal. Specifically, the postorbital process of the squamosal inserts on a deep V-shaped excavation that occupies most of the ventral surface of the caudal ramus of the postorbital (Fig. 13C). The distal end of this ramus is bifid.

### Skull table and neurocranium

#### Frontal

The frontal (Figs. 12 and 13; Table 2) occupies an extensive area of the skull roof. The ectocranial surface of the frontal is 30% longer than wide. It is slightly convex medially, particularly caudomedially near the sagittal plane, around the contact with the parietal. In contrast, the ectocranial surface is gently concave laterally and rostrolaterally near the articulation with the postorbital and prefrontal, respectively. The frontal contributes substantially to the orbit, forming nearly one third of the width of the dorsal orbital margin. Specifically, a subrectangular process extends rostrolaterally from the orbital depression to intervene in between the prefrontal and the postorbital (Figs. 13A and 13C).

**Table 2:** Selected neurocranial measurements (in mm) of the holotype specimen (MSC 7949) of *Eotrachodon orientalis*.

Element	Measurement
Frontal (left), maximum length of ectocranial surface	81
Frontal (left), maximum width of ectocranial surface	63
Frontal (right), maximum length of ectocranial surface	81
Frontal (right), maximum width of ectocranial surface	63
Parietal, maximum length	67
Parietal, maximum width across rostrolateral processes	84
Basisphenoid-parasphenoid length	75
Length from rostral end of cultriform process of parasphenoid to distal end of left basipterygoid process of basisphenoid	84
Basisphenoid, maximum depth of contribution to spheno-occipital tubera	36
Basisphenoid, maximum width of contribution to spheno-occipital tubera	50
Basisphenoid, maximum width across basipterygoid processes	62
Prootic (left), maximum length from rostral margin to distal end of caudal process	65
Supraoccipital, maximum length	40
Supraoccipital, maximum width	66
Fused opithotic-exoccipital (left), maximum length from medial margin to distal tip of paroccipital process	120

The rostral margin of each frontal displays a broad rostrolaterally-oriented finger-like process (Figs. 13A and 13C). The rostral margin of each process is perpendicular to the sagittal edge of the frontal. The rostrolateral corner of this process is recessed and deeply excavated, bearing part of the articular region for the prefrontal and probably the nasal more medially. This articular region for the prefrontal extends further caudally along the rostrolateral margin of the frontal, forming a deep V-shaped concavity adjacent to the contribution of the bone to the orbital margin (Fig. 13A). The frontal becomes gradually shallower rostromedially near the sagittal plane of the skull, medial to the rostral process, where the bone becomes a thin lamina (Fig. 13A). This thinning of the rostromedial margin of the sagittal plane and the lack of articular facets in this area suggests that a fontanelle was present. A thin short peg-like process projects rostrally in-line with the interfrontal suture (Fig. 13A).

The caudal margin of the frontal sutures extensively with the parietal and contributes to the formation of the rostral border of the supratemporal fenestra (Fig. 13A). Ventrally, the frontal exhibits wide and smoothly textured olfactory and orbital depressions that are located rostrally and laterally, respectively (Fig. 13C). A low ridge that is continuous with the rostrolateral corner of the rostral process constitutes the boundary between the olfactory and orbital depressions. The ventral surface of the rostral process is part of the olfactory depression. Caudally and caudolaterally, both depressions are contacted by the rostrodorsal and rostrolateral margins of the orbitosphenoid (Figs. 13C and 13D).

#### Parietal

The parietal (Fig. 13A; Table 2) is roughly hourglass-shaped in dorsal view, and is about 1.9 times as long as it is wide at its narrowest point. Rostrally, the parietal is greatly expanded owing to two large processes that curve and extend laterally. The distance between the distal end of these processes is 2.5 times the width of the parietal at mid-length. They contribute to the rostral margin and rostrodorsal wall of the supratemporal fenestra. A sharp ridge forms the rostrodorsal margin of the proximal region of each lateral process. The ridges are continuous medially with the sagittal crest of the parietal (Fig. 13A). The lateral processes articulate extensively rostrally with the caudal margin of the frontal, ventrally with the lateral process of the laterosphenoid, and laterally with the medial surface of the postorbital. Centered on the sagittal plane of the skull, the parietal displays a fan-shaped median process that forms a crenulated suture with the caudomedial margins of both frontals (Fig. 13A). The dorsal surface of this process is gently depressed.

The main body of the parietal gradually becomes slightly narrower caudally. Its lateral surfaces are moderately concave longitudinally. Dorsally, the parietal shows a tall longitudinal crest that is steeply down-warped rostrally (Fig. 12F). The original morphology of the crest, however, appears to be distorted because of post-depositional dorsoventral crushing. Parietal crests are typically down-warped in lambeosaurine hadrosaurids (Horner, Weishampel & Forster, 2004). Unlike lambeosaurines, the elevation and down-warping of the parietal crest in *Eotrachodon orientalis* is not accompanied by deepening of the squamosal and elevation of the supratemporal bars (Figs. 12F and 13A). Thus, the parietal crest of *E. orientalis* was likely substantially elevated above the supratemporal bars (Figs. 12E and 12F).

The ventral side of the parietal is deeply concave; the lateral margins of this concavity articulate, from rostral to more caudal, with the laterosphenoid, the prootic and the supraoccipital (Fig. 13C). The caudal end of the parietal shows three finger-like projections; at least the median of these projections meets the squamosals dorsally.

#### Supraoccipital

**Figure 14:**
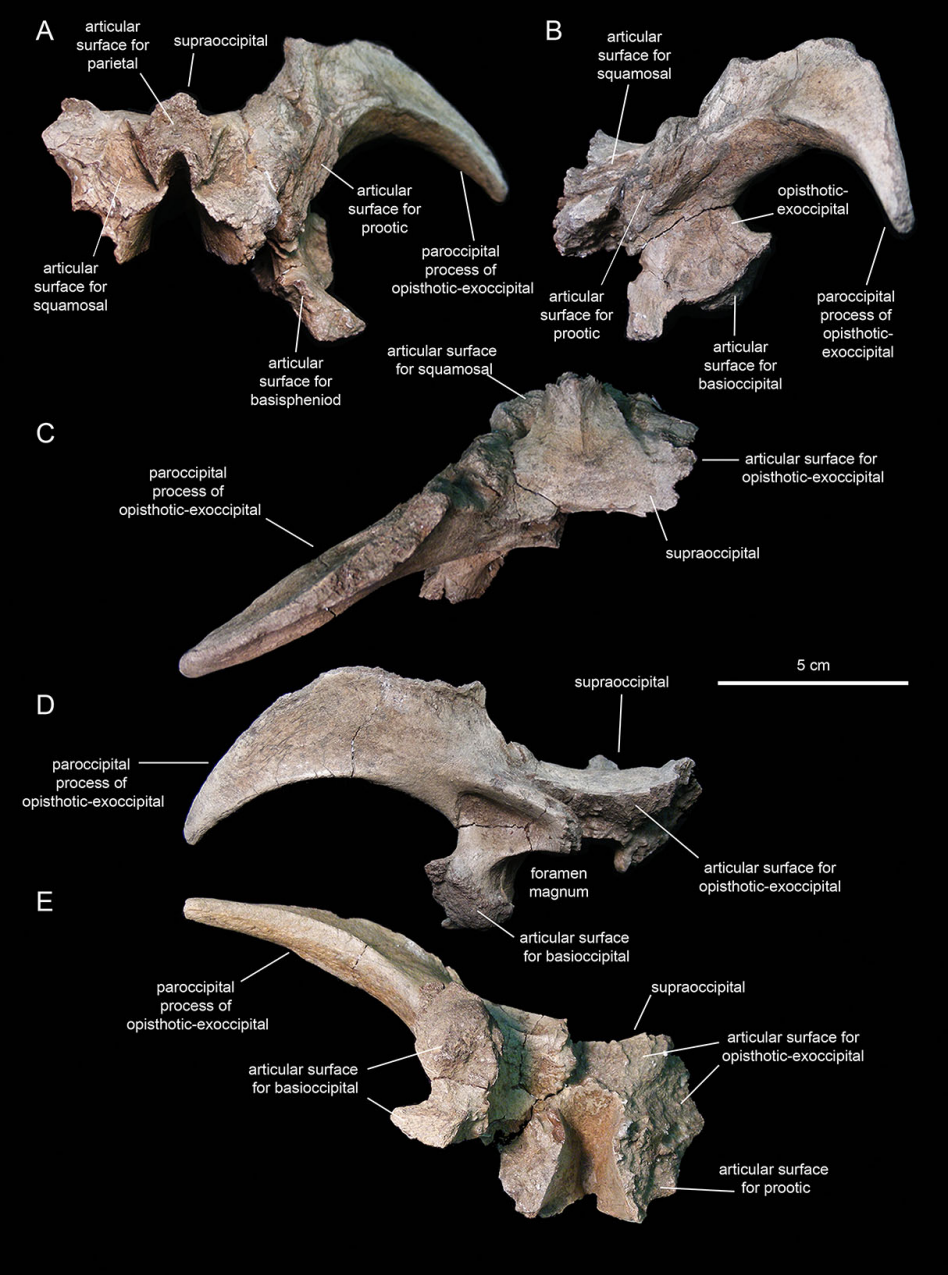
Partial occipital region of the skull of *Eotrachodon orientalis* (holotype MSC 7949). (A) Rostral view. (B) Left lateral view. (C) Dorsal view. (D) Caudal view. (E) Ventral view.

The supraoccipital (Fig. 14; Table 2) is a dorsoventrally compressed and relatively small element that occupies a median position in the occipital region of the neurocranium. This bone rests over the two fused opisthotic-exoccipitals, inset in between the proximal regions of the paroccipital processes of those elements (Fig. 14D). The dorsal surface of the supraoccipital is smooth, gently concave mediolaterally, and mediolaterally expanded caudally. The rostral median region of the supraoccipital is gently elevated rostrodorsally and bisected by a thin ridge that extends caudally to end at about mid-length of the bone (Fig. 14C). That rostral elevation, or ascending process, meets the parietal rostrally (Fig. 14B). Lateral from this process, the supraocciptal displays a rugose rostroventrally-sloping surface for articulation with the squamosal. The ventral side of the supraoccipital is deeply excavated longitudinally (Fig. 14E). Caudally, on the ventral side of the supraoccipital, the subtriangular articular surface for the fused opisthotic-exoccipital faces ventrally and slightly caudally (Fig. 14D).

#### Fused opitshotic-exoccipial

These elements (Fig. 14; Table 2) form most of the occiput and part of the caudodorsal wall of the braincase. The rostral region of this bony complex is mediolaterally compressed and meets the prootic dorsolaterally, the supraoccipital dorsomedially, the basisphenoid rostroventrally, and the basioccipital caudoventrally (Figs. 14A, 14B and 14D). Its lateral surface is dorsoventrally concave and continuous dorsally with the proximolateral surface of the paroccipital process. Ventrally, this region of the lateral wall of the braincase is mediolaterally expanded to form the ventral articular facet for the underlying basioccipital (Fig. 14D).

The paroccipital process is a large horn-like structure that diverges caudolaterally from the caudodorsal corner of the occiput (Fig. 14D). Specifically, the thicker proximal region of this process extends caudodorsally to gradually curve and wedge lateroventrally along its distal segment. The dorsal margin of the paroccipital process forms a ridge that becomes progressively more prominent proximally.

#### Prootic

**Figure 15:**
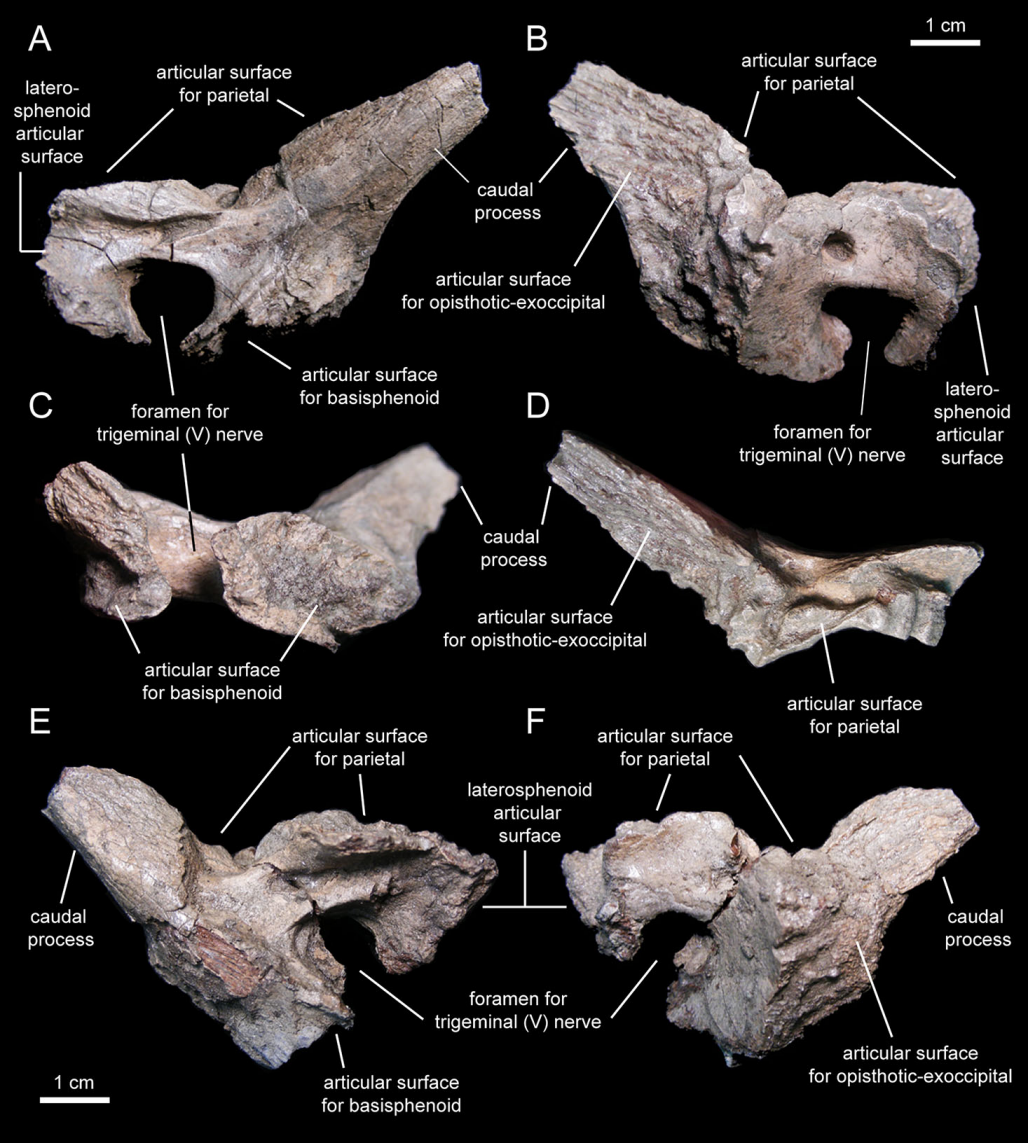
Prootics of *Eotrachodon orientalis* (holotype MSC 7949). (A–D) Left prootic in lateral, medial, ventral, and dorsal view, respectively. (E and F) Right prootic in lateral and medial view, respectively.

The prootic (Fig. 15; Table 2) is a rostrocaudally elongate and relatively small component of the dorsolateral wall of the braincase. The rostral half encloses nearly the entire foramen for the trigeminal nerve, except for the central portion of its ventral margin (Fig. 15A). The dorsal margin of the prootic bears a series of crenulations for articulation with the parietal (Fig. 15D). The ventral margin is slightly expanded mediolaterally, particularly caudal to the trigeminal foramen (Fig. 15C), and meets the basisphenoid. Rostrally, the prootic joins the laterosphenoid.

The caudal half of the prootic consists of a wedge-shaped process that projects caudodorsally and is laterally deflected relative to the rostral half of the bone (Figs. 15A and 15D). The medial surface of this process shows a series of fine longitudinal ridges for articulation with the opisthotic-exoccipital complex (Fig. 15B). A relatively broad but shallow and faint ridge extends caudodorsally on the lateral surface of the prootic (Fig. 15A). This ridge originates caudal to the trigeminal foramen and extends further caudodorsally along the entire lateral surface of the caudal process.

#### Fused parasphenoid-basisphenoid

**Figure 16:**
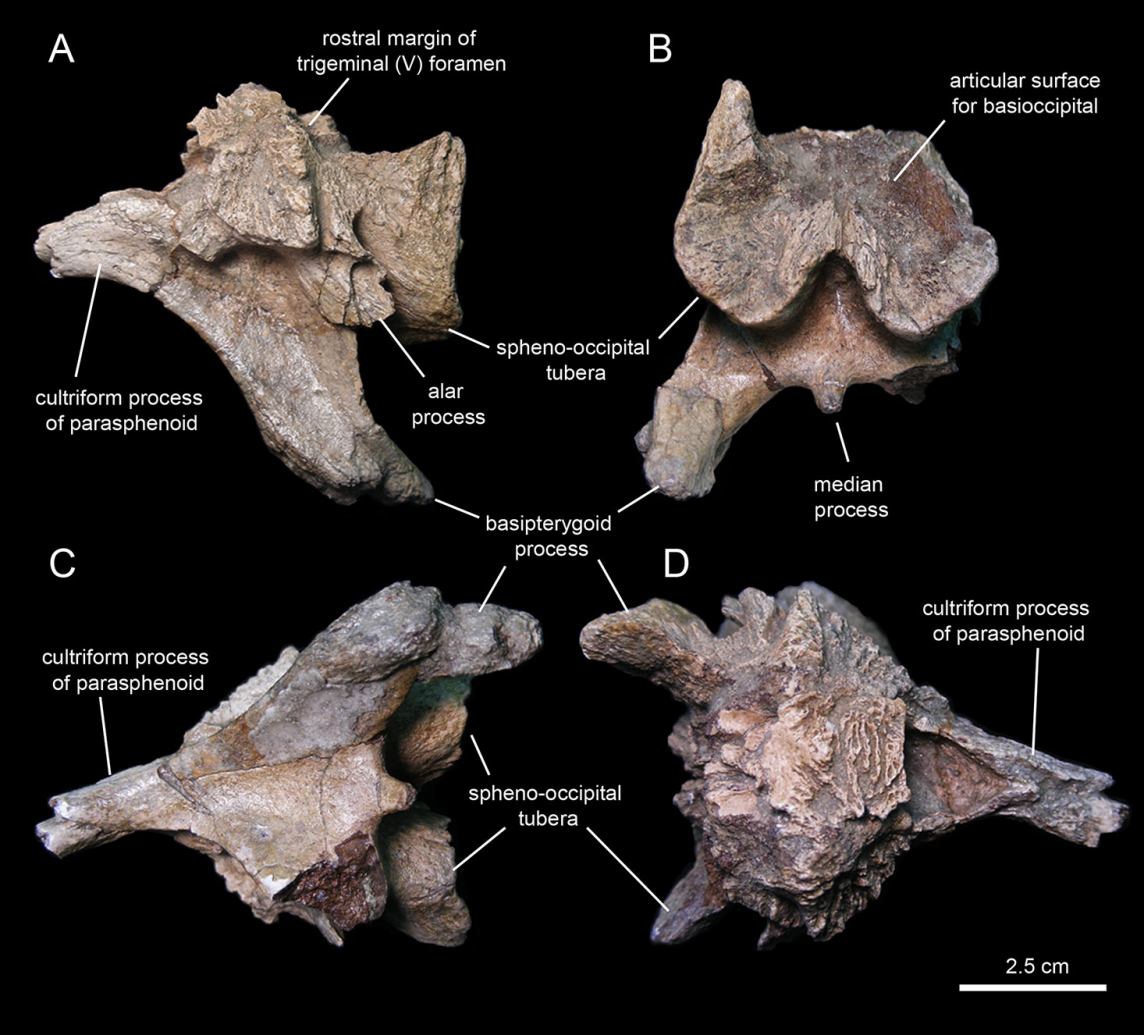
Partial basisphenoid of *Eotrachodon orientalis* (holotype MSC 7949). (A) Left lateral view. (B) Caudal view. (C) Ventral and slightly rostral view. (D) Dorsal view.

This bony complex (Fig. 16A; Table 2) occupies a median position in the rostroventral region of the braincase (Fig. 16). The main body of the basisphenoid is mediolaterally expanded and fused rostrally to the cultriform process of the parasphenoid. Its preserved proximal segment extends rostrally, and is slightly compressed mediolaterally and gently curved ventrally. While the ventral surface of the cultriform process is smooth, the dorsal surface shows a proximal deep triangular excavation, followed by a broad longitudinal sulcus (Fig. 16D).

Caudally, the ventral surface of the cultriform process becomes gradually broader as each of its lateral margins diverges and becomes continuous with the ventral edge of each basipterygoid process (Fig. 16C). The basipterygoid process is a large horn-like structure that projects caudoventrally and laterally to meet the pterygoid bone of the palate. The right pterygoid process of MSC 7949 is only preserved proximally and shows a triangular cross-section; the left process is complete, but its distal end is fractured and medially displaced (Fig. 16B). The extensive ventral surface existing between the bases of the basipterygoid processes is concave and smooth. Its caudal margin shows a short peg-like median process, but unlike most hadrosaurids (Prieto-Márquez, 2010a) lacks a prominent ridge (Fig. 16C).

The caudal region of the basisphenoid consists of the rostral portions of the paired spheno-occipital tubera (Fig. 16A). The tubera are separated ventrally by a deep indentation (Fig. 16B). The caudal articular surfaces of the spheno-occipital tubera are nearly vertically oriented and concave for reception of the basioccipital. The lateral margins of these surfaces are greatly expanded caudolaterally. A sub-elliptical lamina, the alar process, extends caudoventrally from the lateral surface of the proximal region of each spheno-occipital tuber (Fig. 16A). The alar process is relatively small, as in most hadrosauroids except brachylophosaurins (Prieto-Márquez, 2005). The neck-like basisphenoid constriction that occurs in between the basipterygoid processes and the spheno-occipital tubera is moderately developed. Specifically, the minimum width of this constricted area is 50% less than the maximum width of the basisphenoid across the spheno-occipital tubera.

#### Laterosphenoid

The laterosphenoid (Figs. 12C and 13C; Table 2) is a triradiate element that contributes to the laterodorsal wall of the braincase. The lateral surface of the laterosphenoid is gently inflexed rostrocaudally and mediolaterally compressed. Three processes extend from the central region of the laterosphenoid laterally, medially, and ventrally. The rostral border of the laterosphenoid meets the caudal margin of the orbitosphenoid (Figs. 12A and 13C). The caudal margin of the laterosphenoid is slightly expanded mediolaterally and articulates with the rostral border of the prootic. The laterosphenoid meets its counterpart by means of a short but rostrocaudally broad process that extends medially perpendicular to the lateral wall of the braincase (Figs. 12A and 13C).

The large lateral process is perpendicular to the dorsal region of the lateral surface of the body of the laterosphenoid (Figs. 12A, 12C and 13C). The process becomes both substantially shallower and thinner distally. Its ventral side is smooth and broad proximally; distally, however, it gradually becomes mediolaterally compressed to form a ridge that is more prominent near the distal end (Fig. 13C). This ridge separates the orbital cavity from the temporal region of the skull. Along its proximal half, the lateral process underlies the caudolateral region of the frontal; further distally, it underlies the central body of the postorbital. The distal end of the lateral process of the laterosphenoid forms a diarthrosis with a bowl-shaped cavity on the ventral surface of the central region of the postorbital (Fig. 13C).

Below the proximal region of the lateral process, the laterosphenoid extends ventrally forming a thick finger-shaped process (Figs. 12A and 12C). This process wedges ventrally and shows a triangular lateral profile. The ventral process of the laterosphenoid joins the basisphenoid ventrally. Its caudal margin and the caudal border of the central body of the laterosphenoid articulate with the prootic.

#### Orbitosphenoid

The orbitosphenoid (Figs. 12A and 13C; Table 2) forms a substantial part of the rostrodorsal region of the braincase. This is a lateroventrally-facing and dorsoventrally compressed element. It joins the frontal rostrolaterally, the laterosphenoid caudally, the basisphenoid ventrally, and its counterpart medially. The orbitosphenoid would also articulate with the presphenoid rostrally; however, the latter is not preserved in MSC 7949. The external surfaces of both orbitosphenoids are heavily eroded (Fig. 13C), particularly that of the right element, missing neurocranial foramina typically found in these bones such as those transmitting the optic and trochlear nerves (Prieto-Márquez, 2010c).

### Mandible

#### Predentary

**Figure 17:**
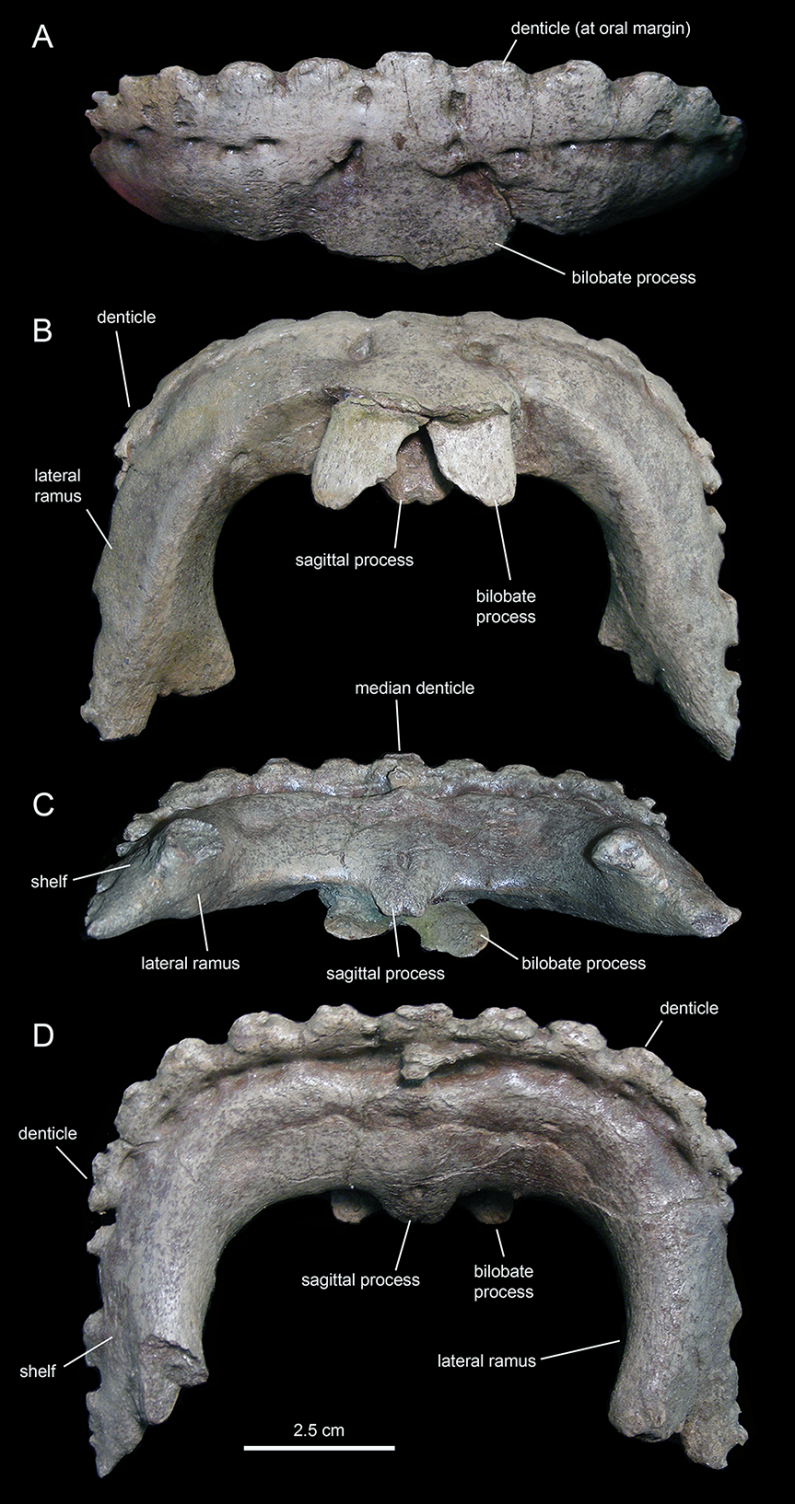
Predentary of *Eotrachodon orientalis* (holotype MSC 7949). (A) Rostral view. (B) Ventral view. (C) Caudal view. (D) Dorsal view.

The predentary (Figs. 3 and 17; Table 3) forms the ventral portion of the iconic hadrosauroid ‘duck-bill.’ This bone is horseshoe-shaped, consisting of a transverse rostral bar and two caudally-projecting lateral rami (Fig. 17). The predentary of *Eotrachodon orientalis* is 1.5 times as wide as it is long. The rostrolateral corner of the bone is somewhat squared in shape. The rostral surface is steeply oriented, forming a 70° angle with the dorsal margin of the lateral rami. The oral margin of the predentary is denticulated. There is a single median denticle, as well as one row of denticles to the left and another row to the right of the median denticle. Each of the rows contain up to eight denticles (Fig. 17D). As in most basal hadrosauroids (Prieto-Márquez, 2010a), the predentary denticles extend beyond the rostral margin onto the dorsal surfaces of the lateral rami. The denticles are rostrocaudally compressed along the rostral oral margin and dorsoventrally compressed along the rami, wider than tall, and with blunt abraded edges. They are larger and subrectangular along the rostral oral margin. Laterally and caudally, the denticles become shallower, smaller, and more irregularly shaped. The dorsal edge of most of the denticles, particularly of those arranged along the rostral margin of the predentary, is ornamented with a pair of small protuberances. On the lingual surface of the predentary, the row of denticles is bounded by a transverse sulcus bearing numerous foramina arranged mediolaterally parallel to the denticles.

**Table 3:** Selected mandibular measurements (in mm) of the holotype specimen (MSC 7949) of *Eotrachodon orientalis*.

Element	Measurement
Predentary, length along the lateral rami	81
Predentary, maximum width	109
Predentary, maximum depth of the rostral surface	43
Dentary (left), length from symphysis to caudal end of coronoid process	280
Dentary (left), depth at mid-length of the dentary ramus	53
Dentary (right), length from symphysis to caudal end of coronoid process	270
Dentary (right), depth at mid-length of the dentary ramus	58
Dentary (right), height from the ventral margin to the apex of the coronoid process	138
Surangular (left), length from the rostral margin of the ascending process to the caudal margin of the retroarticular process	97
Surangular (right), length from the rostral margin of the ascending process to the caudal margin of the retroarticular process	110
Surangular (right), height of ascending process from its dorsal end to the ventral margin of the surangular	96
Angular (partial), maximum length	121
Hyoid (rostral fragment), maximum length	89

A sagittal process projects caudoventrally from the median region of the lingual surface of the predentary (Fig. 17C). Unlike saurolophid hadrosaurids (Prieto-Márquez, 2010a), the lingual surface of the sagittal process of *Eotrachodon orientalis* lacks a median longitudinal ridge (Fig. 17D). Instead, the lingual surface displays a shallow median prominence rostrally and a median foramen near the caudal end of the sagittal process. A bifid tongue-shaped process projects caudoventrally from the median region of the labial surface of the predentary (Fig. 17B). The indentation that separates the two lobes of the process is shallower than the proximal undivided region. The space existing between the two lobes of the process is half as wide as each lobe.

The lateral rami of the predentary extend caudally and are slightly compressed mediolaterally (Figs. 17C and 17D). Distally, the lateral margin of each ramus gradually broadens laterally to form a narrow lateroventrally sloping shelf for articulation with the dentary. Medial to the shelf, the caudomedial corner of the distal end of the lateral ramus is caudally and slightly medially directed. The distal end of the shelf for the dentary extends further caudally than the caudomedial corner of the lateral ramus.

#### Dentary

**Figure 18:**
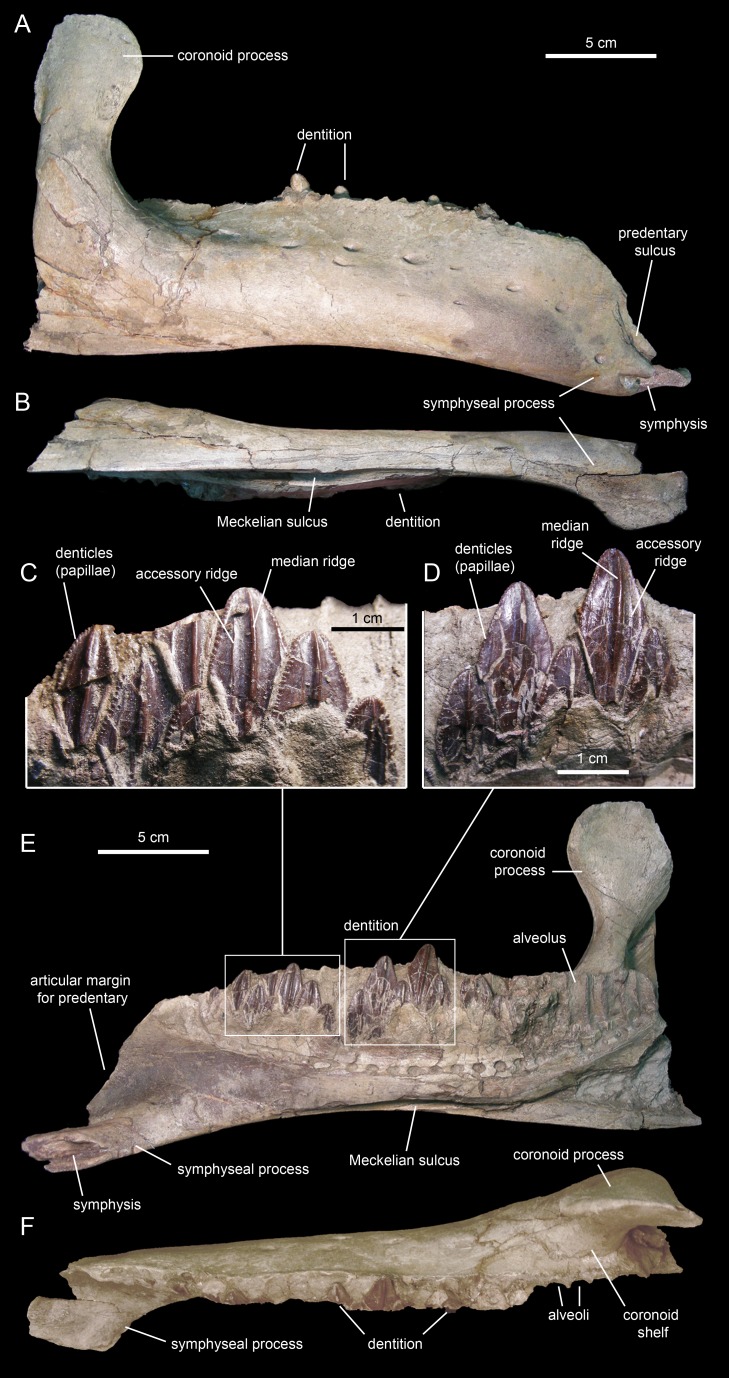
Right dentary of *Eotrachodon orientalis* (holotype MSC 7949). (A) Lateral view. (B) Ventral view. (E) Medial view. (F) Dorsal view. (C and D) Dentary tooth crowns in lingual view.

**Figure 19:**
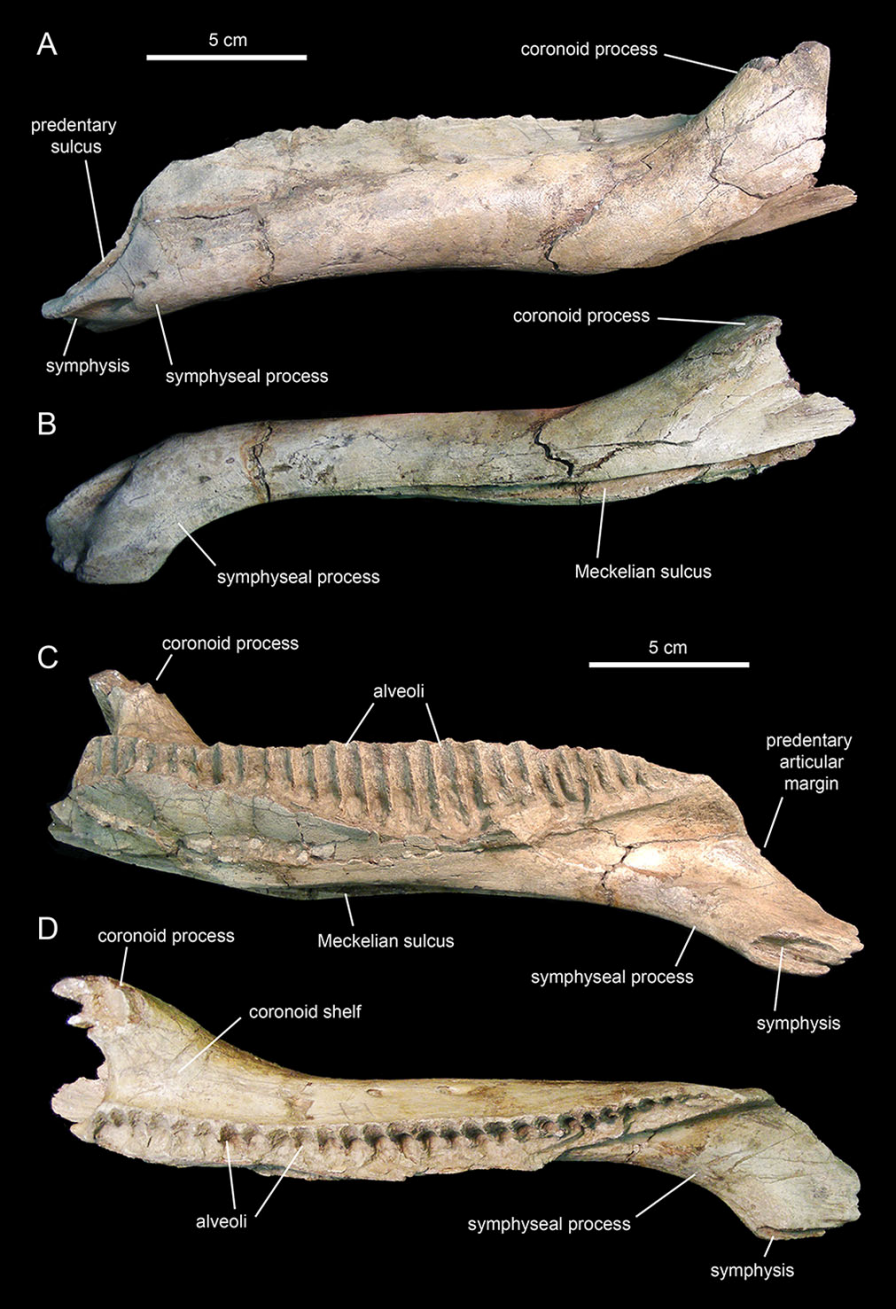
Left dentary of *Eotrachodon orientalis* (holotype MSC 7949). (A) Lateral view. (B) Ventral view. (C) Medial view. (D) Dorsal view.

The dentary (Figs. 18 and 19; Table 3), the tooth-bearing major element of the mandible, is relatively shallow in *Eotrachodon orientalis*; it is about four times as long as it is deep at mid-length. The ventral margin of the dentary lacks a prominent convex lateral profile around the area below the coronoid process, unlike in some hadrosaurids such as *Brachylophosaurus canadensis* (e.g., TMP 90.104.1) or *Prosaurolophus maximus* (e.g., ROM 787). The lateral surface of the dentary is pierced by no less than ten foramina, arranged longitudinally from the area rostral to the base of the coronoid process to the vicinity of the symphyseal process (Fig. 18A).

The proximal edentulous margin is proportionately short, a condition typically present in basal iguanodontians (Norman, 2004). Specifically, in *E. orientalis* this margin is 8% as long as the distance between the rostral-most tooth position and the caudal margin of the coronoid process (Fig. 18E). The dorsal margin of the rostral edentulous articular margin for the predentary is strongly concave in lateral and medial views (Figs. 18A and 18E). The symphyseal process is ventrally deflected, forming a 15° angle with the long axis of the dental battery, as measured in lateral view (Fig. 18A). This deflection originates slightly rostral to the mid-length of the dentary ramus (the ratio between the distance from the caudal margin of the coronoid process to the inflection point of the ventral margin of the dentary and the distance from the caudal margin of the coronoid process to the rostralmost alveolus is 0.68).

The long axis of the occlusal plane is only slightly obliquely oriented relative to the lateral side of the dentary ramus (Fig. 18F), unlike the strong oblique orientation seen in basal hadrosauroids like *Protohadros byrdi* Head, 1998 (e.g., SMU 74582) and *Gilmoreosaurus mongoliensis* Gilmore, 1933 (Prieto-Márquez & Norell, 2010) in which the axis is directed rostrolaterally and forms an angle of at least 15° with the lateral side of the dentary. The dorsal alveolar margin is nearly straight, only slightly bowed lingually in the left dentary (Figs. 18F and 19D). The caudal end of the dental battery lies flush with the caudal margin of the coronoid process (Fig. 18E). In the left dentary, the lateroventral wall of the opening of the Meckelian fossa has post-depositionally collapsed dorsally (Figs. 19A and 19B), causing an apparent extension of the dental battery caudal to the caudal margin of the coronoid process (Fig. 19C).

The coronoid process is vertically oriented and its apex is moderately expanded, taller than wide (Figs. 18A and 18E). The medial surface of the coronoid process, at the opening of the Meckelian fossa, contains fine oblique striations. The coronoid process is laterally offset relative to the tooth row, its base being separated from the dental battery by a narrow concave shelf (Fig. 18F).

There are 26 tooth positions in the dentary. However, given that MSC 7949 is a subadult and that the number of alveoli increases gradually during hadrosauroid ontogeny (Prieto-Márquez, 2010a), a greater tooth count was likely present in mature individuals. Although no wear facets are seen in the dentary teeth, the mediolaterally narrow alveolar space suggests that there were one or, at most two, functional teeth exposed on the occlusal plane. This contrasts with the derived count of three functional teeth present in hadrosaurids (Prieto-Márquez, 2010a). Again, such a low count of functional teeth in the dentary of MSC 7949 may have been greater in adult specimens of *Eotrachodon orientalis*.

#### Surangular

**Figure 20:**
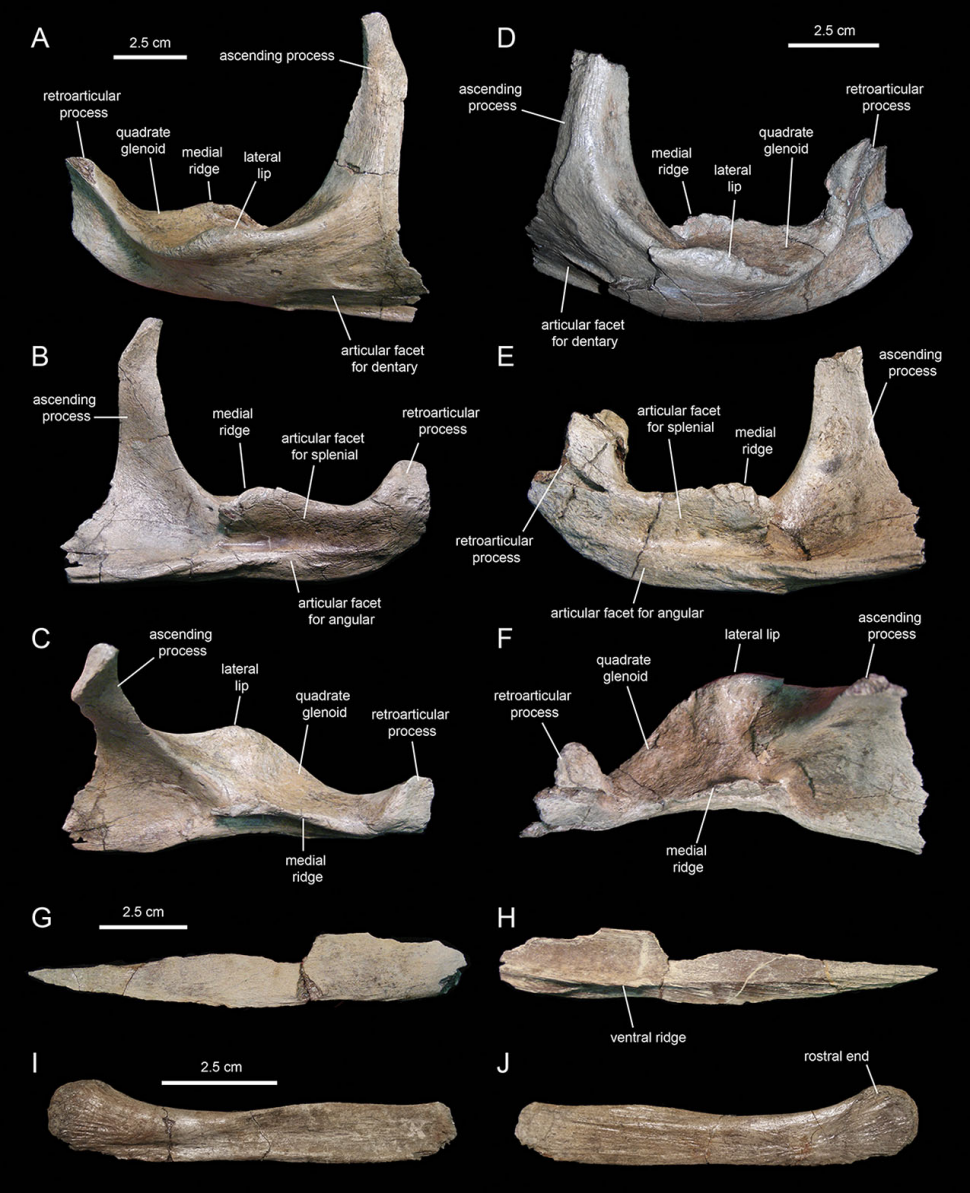
Postdentary mandibular elements of *Eotrachodon orientalis* (holotype MSC 7949). (A–C) Right surangular in lateral, medial, and dorsal view, respectively. (D–F) Left surangular in lateral, medial, and dorsal view, respectively. (G and H) Right angular in medial and lateral view, respectively. (I and J) Right hyoid in lateral and medial view, respectively.

The surangular (Figs. 20A–20F; Table 3) is the major post-dentary bone in the caudal region of the mandible. The rostral third of the surangular consists of a deeply excavated dorsal surface and a long ascending process that projects dorsally from the rostromedial margin of the surangular. This process is strap-like, mediolaterally compressed, and gradually narrows distally to a thin sliver (Figs. 20A and 20B). The rostrolateral margin of the process is overlapped by the caudolateral region of the dentary. The smooth caudolateral surface of the ascending process is caudoventrally continuous with the dorsal surface of the lateral lip and the quadrate glenoid. The rostroventral and ventral surfaces of the rostral region of the surangular, below the ascending process, contain elliptical recessed areas for reception of the overlapping caudoventral region of the dentary. No foramina are present.

Caudal to the ascending process, a large ridge raises from the medial margin of the dorsal surface of the surangular (Figs. 20B and 20C). The medial side of this ridge constitutes an extensive articular surface for the splenial. Adjacent and below this surface, there is a long D-shaped facet for articulation with the angular that forms part of the ventral margin of the surangular (Fig. 20B).

Lateral to the medial ridge lays the glenoid surface that receives the ventral end of the quadrate and, immediately rostrally, the lateral lip of the surangular (Figs. 20A and 20D). As in other hadrosaurids, the convex side of the lateral lip and the lateroventral surface of the main body of the surangular face more ventrally than laterally. Caudally, the distal third of the surangular becomes slightly compressed mediolaterally and ends in the retroarticular process (Figs. 20B and 20C). This process extends rostrodorsally and is laterally deflected. Its lateral surface is deeply depressed by a longitudinal concavity that extends further rostrally under the quadrate glenoid (Fig. 20A). In contrast, the medial surface of the retroarticular process is convex for reception of the articular, a bone that caps the caudomedial end of the mandible (Fig. 20B).

#### Angular

The angular (Figs. 20G and 20H; Table 3) is a mediolaterally compressed rod-like element in the post-dentary region of the mandible. The only known angular of *Eotrachodon orientalis* is missing its dorsal margin and the distal end of its caudal segment. The entire lateral surface of this element attaches to the ventral region of the medial side of the surangular, ventral to the splenial. The angular becomes slightly deeper along the caudal third. The bone gradually wedges rostrally and tapers to a point. A longitudinal shallow ridge extends on the lateral surface of the angular throughout its length (Fig. 20H). Along the rostral two thirds of the angular, the narrow surface ventral to this ridge is textured with a series of short oblique striations.

### Accessory cranial elements

#### Hyoid

MSC 7949 preserves the rostral half of the right first ceratobranchial (Figs. 20I and 20J; cf. Ostrom, 1961). This bone underlies the post-dentary caudal region of the mandible, probably medial to the ventral margin of the angular, following to the position seen in the few available hadrosaurid specimens with articulated hyoid elements (e.g., *Brachylophosaurus canadensis*, MOR 794). The hyoid fragment is rod-like. Rostrally, it becomes slightly shallower before gently curving dorsally and dorsoventrally expanded to form a semicircular rostral end. The lateral side is slightly concave longitudinally, with exception of the dorsoventrally convex rostral end; these two areas are separated by a shallow and long oblique ridge. The medial surface is flat, except for the gently depressed rostral end.

#### Dentition

**Figure 21:**
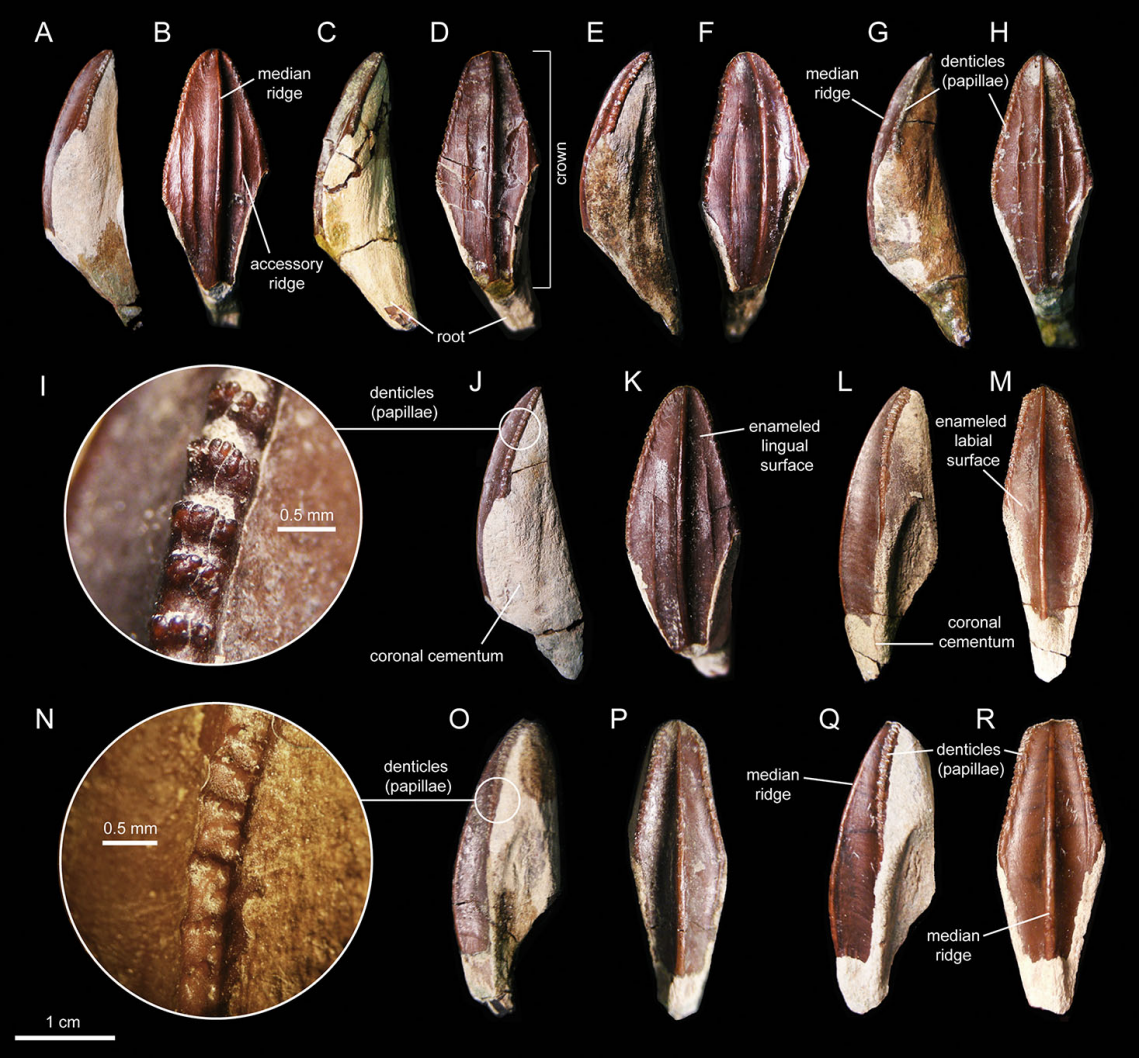
Dentary and maxillary teeth of *Eotrachodon orientalis* (holotype MSC 7949). (A–K) Dentary teeth. (I) Detail of marginal denticles of a dentary tooth. (L–R) Maxillary teeth. (N) Detail of marginal denticles of a maxillary tooth.

The enameled lingual sides of the dentary tooth crowns are diamond-shaped (Figs. 21A–21K). Those teeth located within the rostral third of the dental battery show a height/width ratio of 2.2, whereas those found at mid-length of the battery are slightly taller with a ratio up to 2.4. There is a median primary ridge that is surrounded mesially and distally by one or two thinner accessory ridges. In some instances, the primary ridge does not occupy a median position in the crown, but it is distally offset. Some teeth exhibit a split median ridge. Marginal denticles consist of small papillae that are similarly sized in both margins of the tooth. Each papilla consists of a ledge containing four to six knob-like protrusions that are oriented apically (Fig. 21I). The mesial margin overlaps the distal margin of the adjacent crown.

Maxillary tooth crowns are taller than those of the dentary, with height/width ratios that range from 2.5–3.1. The apices of maxillary teeth are blunter than those of dentary teeth (Figs. 21L–21R). The apical parts of some crowns show a gentle labial curvature. There is only a single prominent median ridge on the enameled sides of these teeth. Like in the dentary, the maxillary denticles consist of mammilated ledges consisting of four to five apically oriented, knob-like protrusions (Fig. 21N). Functional teeth exposed at the occlusal plane display gently concave wear facets.

### Axial skeleton

#### Cervical vertebrae

**Figure 22:**
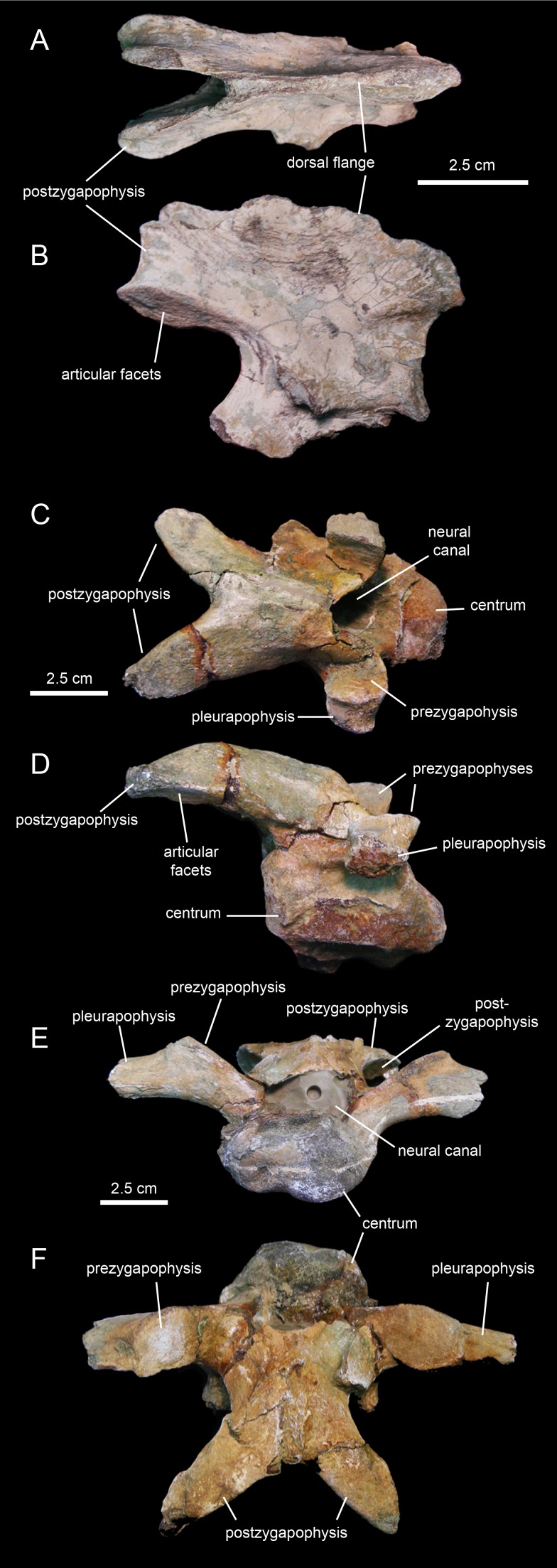
Cervical vertebrae of *Eotrachodon orientalis* (holotype MSC 7949). (A and B) Axis in dorsal and right lateral view, respectively. (C and D) Cervical vertebra in dorsal and right lateral view, respectively. (E and F) Another more posterior cervical vertebra in cranial and dorsal view, respectively.

The axis of MSC 7949 (Figs. 22A and 22B; Table 4) is incompletely preserved, consisting solely of the dorsal flange of the neural spine, the postzygapophyses, and the left and part of the right pedicel of the neural arch. The dorsal flange of the axis is approximately twice as long as it is deep. Except for the hook-shaped cranial end, the dorsal margin of the flange is heavily eroded. Its caudal region is continuous with the two short postzygapophyses. These processes diverge caudolaterally from the sagittal plane of the axis. They are mediolaterally compressed and expand ventrally to bear the articular facets for the prezygapophyses of the cervical 3. The latter are elliptical facets that are ventrally and slightly laterally oriented.

**Table 4:** Selected axial measurements (in mm) of the holotype specimen (MSC 7949) of *Eotrachodon orientalis*.

Element	Measurement
Axis, maximum length from preserved cranial end of dorsal flange of neural spine to the caudal end of the postzygapophyses	87
Axis, maximum width across the postzygapophyses	35
Cervical vertebra in Figs. 22C and 22D, maximum length of the centrum	59
Cervical vertebra in Figs. 22C and 22D, maximum width across pleurapophyses	73
Cervical vertebra in Figs. 22C and 22D, length from the dorsal margin of the neural canal to the caudal end of the right postzygapophysis	74
Cervical vertebra in Figs. 22E and 22F, maximum length of the centrum	78
Cervical vertebra in Figs. 22E and 22F, maximum width across pleurapophyses	169
Cervical vertebra in Figs. 22E and 22F, length from the dorsal margin of the neural canal to the caudal end of the right postzygapophysis	89
Dorsal vertebra in Figs. 23A and 23B, length of the centrum	82
Dorsal vertebra in Figs. 23A and 23B, maximum height of the centrum	42
Dorsal vertebra in Figs. 23A and 23B, maximum width across pleurapophyses	64
Dorsal vertebra in Figs. 23A and 23B, preserved length of the neural spine	161
Dorsal vertebra in Figs. 23C and 23D, length of the centrum	73
Dorsal vertebra in Figs. 23C and 23D, maximum height of the centrum	48
Dorsal vertebra in Figs. 23C and 23D, maximum width across pleurapophyses	107
Dorsal vertebra in Figs. 23C and 23D, preserved length of the neural spine	142
Dorsal vertebra in Figs. 23E and 23F, length of the centrum	48
Dorsal vertebra in Figs. 23E and 23F, maximum height of the centrum	51
Dorsal vertebra in Figs. 23E and 23F, max. width across pleurapophyses (estimated)	100
Dorsal vertebra in Figs. 23E and 23F, preserved length of the neural spine	139
Fused sacrals in Figs. 24A and 24B, combined length of the three preserved centra	204
Fused sacrals in Figs. 24A and 24B, height of preserved neural spine	148
Caudal vertebra in Figs. 24G and 24H, maximum transversal width of the centrum	66
Caudal vertebra in Figs. 24G and 24H, maximum height of the centrum	75

The few preserved post-axial available cervical vertebrae (Figs. 22C–22F) show centra that are wider than deep, strongly opisthocoelous, craniocaudally elongate, and dorsoventrally compressed. These centra are elliptical to heart-shaped in caudal view, with concave caudodorsal margins. The neural arches are craniocaudally elongate and enclose large neural canals. The postzygapophyses are arched caudoventrally to meet the prezygapohyses of successive vertebrae (Figs. 22C and 22D). In dorsal view, the two postzygapophyses of each cervical vertebra form a V-shaped structure. The large elliptical articular facets lay on the lateroventral sides of the distal ends of the postzygapophyses. The prezygapohyses are oval and dorsomedially oriented, lying on the dorsal surface of the proximal region of the pleurapophysis. The latter extend laterally and slightly dorsally from the dorsolateral surface of the centra. The neural spines are shallow ridges that rise from the sagittal edges of the neural arches.

#### Dorsal vertebrae

**Figure 23:**
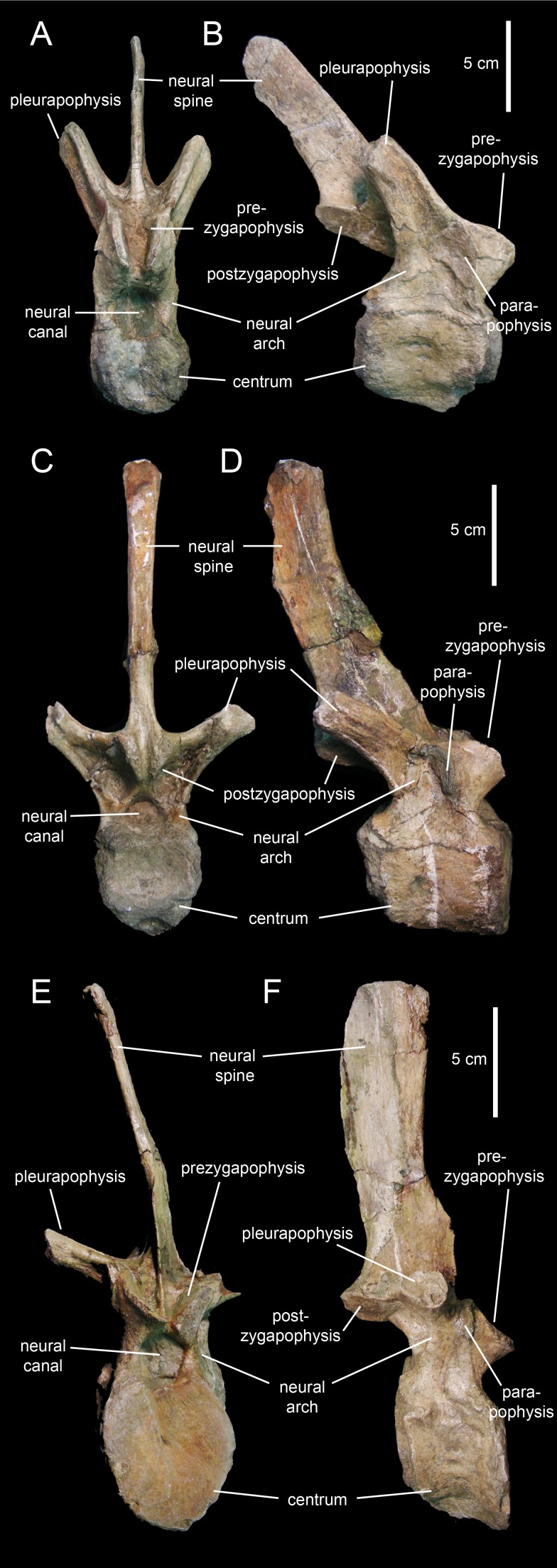
Dorsal vertebrae of *Eotrachodon orientalis* (holotype MSC 7949). (A and B) Cranial dorsal vertebra in cranial and right lateral view, respectively. (C and D) Dorsal vertebra in caudal and right lateral view, respectively. (E and F) Posterior dorsal vertebra in cranial and right lateral view, respectively.

The centra of the proximal dorsal vertebrae are heart-shaped in caudal view and less opisthocoelous than those of the cervicals (Figs. 23A–23D; Table 4). In ventral view, these centra are hourglass-shaped. A median longitudinal ridge is present on their ventral sides. The neural arches show thicker walls than in the cervical vertebrae and enclose smaller neural canals (Figs. 23A and 23C). The pleurapophyses extend from the base of the neural arch. These thick processes are wing-shaped in dorsal view and project caudodorsally and laterally (Figs. 23B and 23D). Their lateral and caudal surfaces are longitudinally concave, more so proximally than distally. The articular facets of the prezygapophyses are elliptical and face mediodorsally above the cranial margin of the neural arches. The neural spines are blade-like and caudodorsally projected. The postzygapophyses are found under the proximal extent of the neural spines and bear elliptical facets oriented lateroventrally.

Progressively more distal dorsal vertebrae show shorter centra (Figs. 23E and 23F). The neural canals become narrower and the pedicels of the neural arches are thicker. The pleurapophyses gradually shift to a more horizontal orientation. The neural spines become broader and more vertically oriented. Apically, the neural spines become gradually thicker, particularly in the middle dorsal vertebrae, the apices of which are club-like. The neural spine height/maximum centrum depth ratio varies from 3.25 in the proximal and middle dorsals to only 2.5 in the more distal dorsal vertebrae. The articular facets of the prezygapohyses and postzygapophyses are less elongate than in the proximal dorsal vertebrae, and face dorsomedially and ventrolaterally, respectively.

#### Sacrum

**Figure 24:**
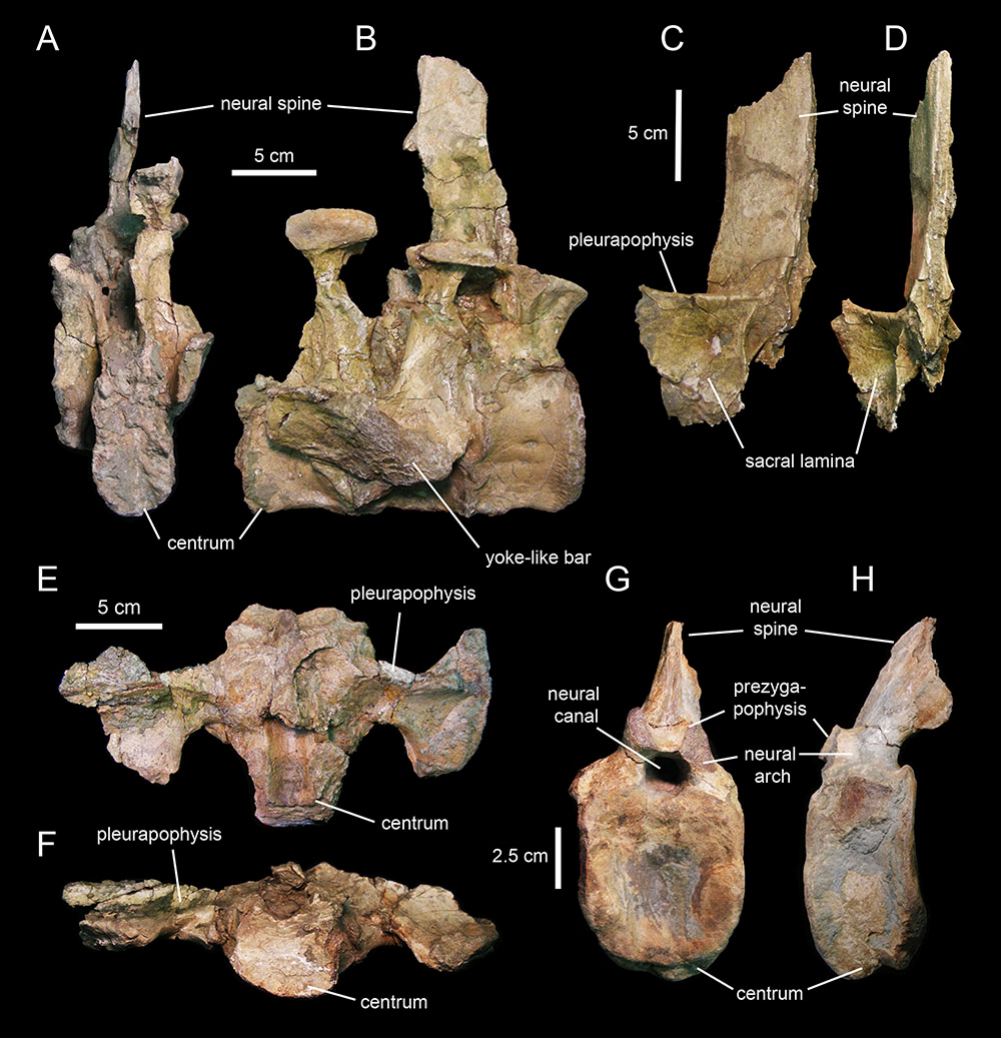
Sacral and caudal vertebrae of *Eotrachodon orientalis* (holotype MSC 7949). (A and B) Partial sacrum in caudal and right lateral view, respectively. (C and D) Partial sacral neural arch in left caudolateral and caudal view, respectively. (E and F) Partial sacral centrum in dorsal and cranial view, respectively. (G and H) Partial proximal caudal vertebra in cranial and left lateral view, respectively.

The sacrum (Figs. 24A–24F; Table 4) is known from a few fragments that include three co-ossified sacral vertebrae. The other sacral vertebrae appear to be separated from those three: the elements at the extremities of the row show the rough articular surface of the centra suggesting that they where not fused to the adjacent vetebrae. Sacral centra become wider and shorter caudally in the sacrum. The pleurapophyses are short, robust, and project laterally near the base of the neural spines (Figs. 24E and 24F). Triangular laminae extend ventrally from the base of the pleurapophyses (Figs. 24A–24D). Such laminae would also fuse with a robust yoke-like bar that extends longitudinally at mid-depth of the centra (Figs. 24B and 24E). This bar connects at least some of the co-ossified sacral vertebrae to the medial surface of the ilium. The neural spines are broad subrectangular laminae that rise nearly vertically from the dorsal region of the neural arches.

#### Caudal vertebrae

MSC 7949 preserves a minimum of 36 caudal vertebrae (Figs. 24G and 24H; Table 4). The proximal caudals show craniocaudally compressed and mediolaterally wide centra (Figs. 24G and 24H). These centra show oval to subrectangular cranial and caudal surfaces, and concave lateral sides. More distal caudal centra, however, become gradually more elongate and have hexagonal cranial and caudal facets, as well as dorsoventrally convex lateral surfaces. The articular facets for the haemal arches are nearly flat to slightly convex facets at the lateroventral corners of the centra. Their precise shape is however uncertain given that these facets have experienced a certain degree of abrasion. No ventral keel is present in these vertebrae. The neural arches of all caudals lack a visible suture with the centra, showing thicker pedicels and enclosing narrower neural canals than those of the pre-caudal vertebrae (Fig. 24G). Only the proximal-most extent of the pleurapophyses is preserved in the available proximal caudals; distal-most caudal vertebrae lack pleurapophyses. When present, the processes attach to the dorsolateral border of the centra, near the lateral margins of the neural arches. The neural spines are thick and oval in cross section. They project caudodorsally in the more proximal caudals; distally throughout the tail, however, they gradually curve rostrodorsally and become more caudally than dorsally oriented. The prezygapophyses bear small oval facets that face medially and slightly dorsally. These facets become slightly smaller in the more distal caudals. The postzygapophyses occur on the ventral surface of the base of the neural spines; their articular facets face laterally and slightly ventrally. In the more distal caudals, the position of the postzygapophyses shifts further dorsally relative to the base of the neural spines.

### Appendicular skeleton

#### Pubis

**Figure 25:**
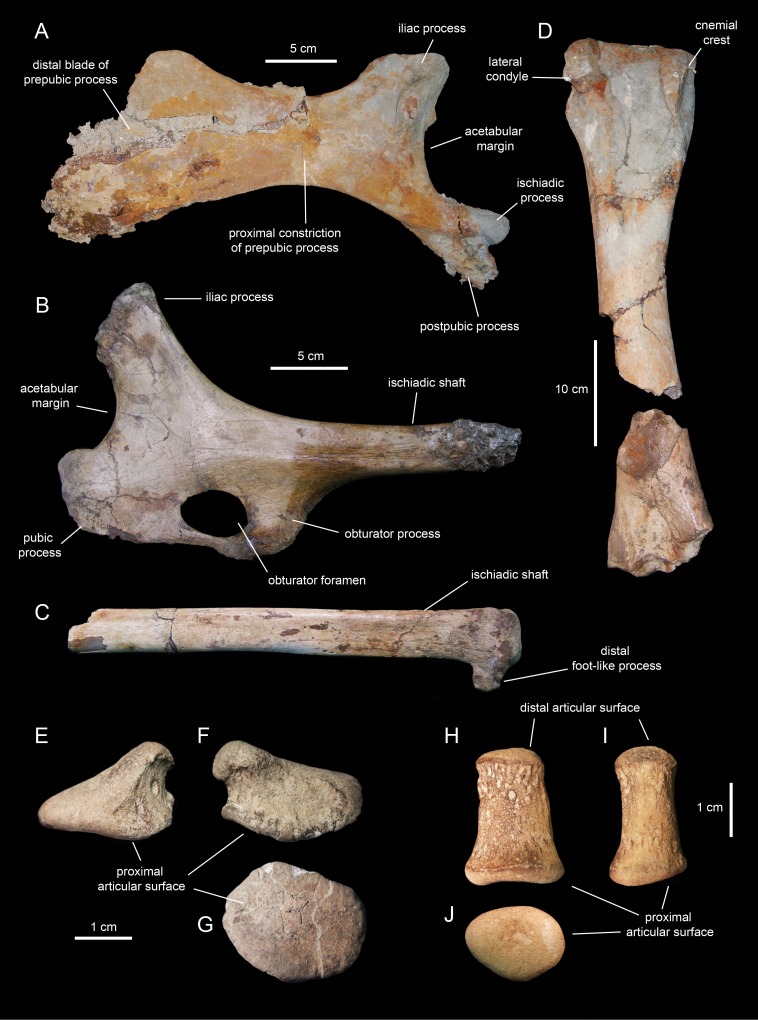
Appendicular elements of *Eotrachodon orientalis* (holotype MSC 7949). (A) Partial left pubis in lateral view. (B) Partial left ischium in lateral view. (C) Distal shaft of the left ischium shown in B, in lateral view. (D) Partial right tibia in lateral view. (E–G) Left manual phalanx III-2 in dorsal, palmar, and proximal views, respectively. (H–J) Left manual phalanx V-2 in dorsal, lateral, and distal views, respectively.

The pubis of MSC 7949 (Fig. 25A; Table 5) is nearly completely preserved, lacking only the craniodorsal region of the distal blade of the pubic process and the shaft of the postpubic process. *Eotrachodon orientalis* has a relatively short pubis, its total length slightly more than double the maximum width of the acetabular margin. The bone consists of two main areas: a proximal region containing the iliac and ischiadic processes for articulation with the other pelvic bones and a distal region consisting of the prepubic process (Fig. 25A). The prepubic process consists of a paddle-shaped distal blade and a proximal neck-like dorsoventrally constricted area. The distal blade is more ventrally than dorsally expanded, as in all saurolophine hadrosaurids and *Lophorhothon atopus* (AUMP 2295), and is deeper than the acetabular margin. The proximal constriction is longer than, and about half as deep as the distal blade. The maximum depth of the distal prepubic blade is slightly deeper than the maximum breadth of the acetabular margin. The maximum concavity of the dorsal margin of the prepubic constriction lies approximately above the maximum concavity of the ventral margin.

**Table 5:** Selected appendicular measurements (in mm) of the holotype specimen (MSC 7949) of *Eotrachodon orientalis*.

Element	Measurement
Pubis, length from the caudal margin of the ischiadic process to the distal end of the incomplete prepubic blade	333
Pubis, length from the caudal margin of the iliac process to the distal end of the incomplete prepubic blade	287
Pubis, minimum depth of the proximal constriction	61
Pubis, maximum depth (perpendicular to the longitudinal axis of the pubis) of the incomplete distal blade of the prepubic process	131
Ischium, maximum length from the rostral margin of the pubic process to the caudal border of the obturator process	152
Ischium, height from the caudodorsal margin of the iliac process to the ventral margin of the pubic process	161
Ischium, depth of the proximal segment of the ischiadic shaft	30
Tibia (incomplete shaft and distal and proximal ends), maximum length	522
Tibia, estimated total length	530
Tibia, craniocaudal width of the incomplete proximal end	127
Tibia, minimum mediolateral diameter of the shaft	66
Manual phalanx III-2, maximum proximodistal width	19
Manual phalanx III-2, maximum diameter of the distal facet	28
Manual phalanx V-2, maximum length	25
Manual phalanx V-2, maximum diameter of the distal facet	18

The dorsal margin of the proximal constriction of the prepubic process is caudally continuous with the iliac process of the pubis (Fig. 25A). This tetrahedral process rises dorsally and slightly caudally from the proximal region of the pubis. Its concave caudal surface forms most of the acetabular margin of the pubis. The ischiadic process extends caudoventrally to form the rostroventral border of the acetabular margin. This process is one and a half times as long as it is wide and its proximal extent displays a prominent lateroventral protuberance. The postpubic process is found adjacent and medial to the ischiadic process and projects caudoventrally, being more ventrally oriented than the ischiadic process. The divergent orientations of both processes form an angle of 24°.

#### Ischium

The ischium of MSC 7949 (Figs. 25B and 25C; Table 5) preserves the proximal plate and the proximal-most and distal segments of the shaft. The iliac process extends craniodorsally from the dorsal region of the proximal plate and it is relatively short, being only one and a half times as long as its articular end is wide. The concave dorsal and ventral margins of the iliac process are continuous with the ischiadic shaft and the sharp acetabular margin, respectively. As in saurolophines, the iliac process is straight (Fig. 25B), lacking the caudally recurved articular margin present in basal hadrosauroids and lambeosaurines (Brett-Surman & Wagner, 2007). The pubic process is subrectangular and bounds ventrally the acetabular margin of the ischium. This process extends a short distance cranially, being twice as deep as it is long.

Most of the lateral surface of the iliac process and the dorsal half of that of the pubic process, as well as the lateral surface surrounding the obturator foramen, are slightly depressed (Fig. 25B). The obturator process extends ventrally from the area where the proximal plate and the ischiadic shaft meet. The process is mediolaterally compressed and broader proximally than distally. Cranially, it is continuous with the caudal extent of the pubic process, contributing ventrally and caudally to the full closure of the large obturator foramen (Fig. 25B). The medial surface of the proximal plate of the ischium is flat. A sharp ridge extends ventrally from the caudomedial margin of the obturator process to the medial surface of the proximal region of the ischiadic shaft. The distal end of the shaft shows a small portion of a foot-like process (Fig. 25C), a structure present in basal hadrosauroids and lambeosaurine hadrosaurids (Prieto-Márquez, 2010a). This process is rostroventrally oriented relative to the horizontal shaft.

#### Tibia

The tibia of MSC 7949 (Fig. 25D; Table 5) is partially preserved, missing portions of the proximal end, part of the shaft, and the distal end. This element consists of a sub-cylindrical shaft that expands proximally and distally. Specifically, the proximal region gradually becomes mediolaterally compressed and craniocaudally expanded. Erosion of the proximal end caused the loss of the internal condyle, as well as the truncation of the lateral condyle and the cnemial crest that forms the cranial margin of this tibial region. At its minimum diameter, which corresponds to approximately mid-length of the tibia, the shaft is half as wide as the proximal end of the bone. The distal region of the tibia is craniocaudally compressed and mediolaterally expanded. Its cranial surface is slightly depressed.

#### Manual phalanx III-2

This phalanx (Figs. 25E–25G; Table 5) is a wedge-shaped proximodistally compressed bone. The phalanx wedges towards the lateral side of digit III. The proximal surface of phalange III-2 is oval and gently concave. The distal surface is smooth and palmodorsally convex. There is a distinctive, slightly recessed band surrounding most of the perimeter of phalange, except for the more compressed lateral margin. This recessed surface is more extensive on the palmar side of the phalanx.

#### Manual phalanx V-2

This phalanx (Figs. 25H–25J; Table 5) is subconical and palmodorsally compressed. The dorsal and palmar surfaces are proximodistally concave, particularly the dorsal surface. The proximal and distal articular surfaces of the phalanx are oval. The proximal facet is gently concave and one and a half times wider than the distal surface. The medial side of the phalanx is slightly deeper than the lateral side.

## Comparisons with Other Appalachian Hadrosauroids

Aside from *Eotrachodon orientalis*, Appalachia has hitherto provided only a few partial postcranial and fragmentary cranial remains belonging to Hadrosauridae, with only one recognized species, *Hadrosaurus foulkii* Leidy, 1858 (Campanian of New Jersey; Prieto-Márquez, Weishampel & Horner, 2006). Other hadrosaurid species from the Appalachian fossil record are nomina dubia (Prieto-Márquez, Weishampel & Horner, 2006). These non-diagnostic taxa are known from fragmentary material and include *Hadrosaurus tripos* Cope, 1869 (see also Baird & Horner, 1979), *H. minor* Marsh, 1870, *H. cavatus* Cope, 1871, *Ornithotarsus immanis* Cope, 1869, *Hypsibema crassicauda* Cope, 1869, and *Parrosaurus missouriensis* Gilmore & Stewart, 1945.

Two other taxa, *Claosaurus agilis* (upper Coniacian of Kansas, a taxon based on a specimen that is missing almost the entire skull; Carpenter, Dilkes & Weishampel, 1995) and *Lophorhothon atopus* Langston, 1960 (lower Campanian of Alabama, partial skulls and postcrania), are hadrosauroids closely related to, but outside, the Hadrosauridae as defined in some recent phylogenetic hypotheses (Prieto-Márquez & Norell, 2010; Gates & Scheetz, 2014; Prieto-Márquez, 2014; Prieto-Márquez, Erickson & Ebersole, 2016).

### Hadrosaurus foulkii

**Figure 26:**
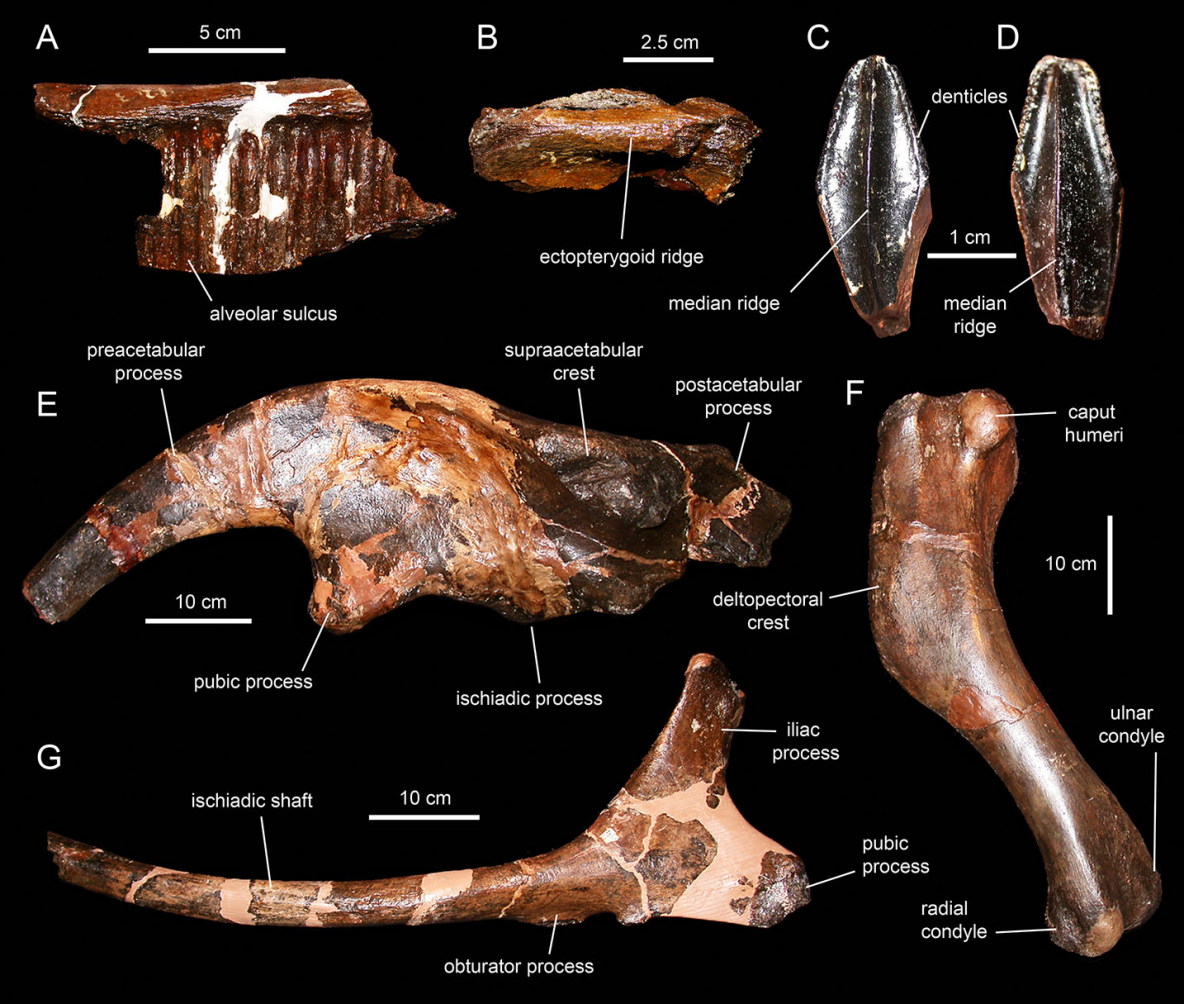
Selected cranial and appendicular elements of *Hadrosaurus foulkii*. (A) Fragment of maxilla in medial view (ANSP 9203), showing the alveolar sulci of the dental battery. (B) Fragment of the caudal region of a maxilla in lateral view (ANSP 9204). (C) Dentary tooth crown in lingual view (ANSP 9201). (D) Maxillary tooth crown in labial view (ANSP 9201). (E) Left ilium in lateral view (ANSP 10005). (F) Left humerus in caudolateral view (ANSP 10005). (G) Right ischium in lateral view (ANSP 10005).

*Hadrosaurus foulkii* is known from a partial skeleton and a few cranial fragments collected from Campanian strata of the Woodbury Formation of Haddonfield, New Jersey, USA (Leidy, 1858; Prieto-Márquez, Weishampel & Horner, 2006; Fig. 26). *H. foulkii* is diagnosed on the basis a unique anatomical combination consisting of a short humeral deltopectoral crest (slightly over 40% of the length of the humerus) that displays a wide and arcuate laterodistal corner (Fig. 26F) and an ilium with supraacetabular crest being half the length of central iliac plate, with ventral apex of the crest located above the caudal tuberosity of the ischiadic process (Fig. 26E; Prieto-Márquez, 2011b). The humerus and ilium are not preserved in *Eotrachodon orientalis* negating the possibility for direct comparison between these taxa with regard to these features.

The skull of *Hadrosaurus foulkii* is known solely from two fragments of the maxilla, various fragments of dental battery, isolated maxillary and dentary teeth; a postcranium preserving three proximal dorsal and 12 caudal vertebrae, a coracoid, humerus, radius, ulna, ilium, ischium, femur, tibia, fibula, and partial pes (Prieto-Márquez, Weishampel & Horner, 2006). Among the few overlapping elements that exist between *H. foulkii* and *Eotrachodon orientalis*, only the teeth and ischia are taxonomically informative and are sufficiently complete for comparison. *H. foulkii* is distinct from *E. orientalis* in having dentary teeth lacking accessory ridges (Fig. 26C) and a dorsally curved shaft of the ischium (Fig. 26G).

### Claosaurus agilis

**Figure 27:**
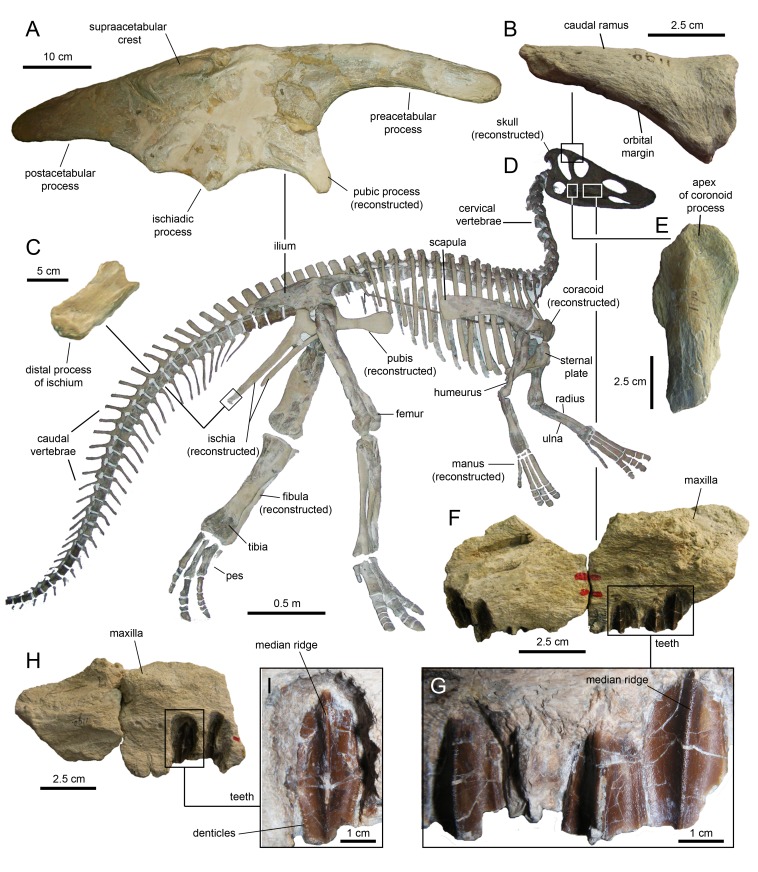
Partial skeleton of *Claosaurus agilis* (holotype YPM 1190). (A) Right ilium in lateral view. (B) Partial postorbital in lateral view. (C) Distal process of the right ischium in lateral view. (D) Mounted partial skeleton of YPM 1190. (E) Coronoid process of the right dentary in lateral view. (F) Fragment of maxilla in lateral view. (G) Detail of the maxillary tooth crowns in (F). (H) Fragment of maxilla in lateral view. (I) Detail of a maxillary tooth crown in (H).

*Claosaurus agilis* is known from a single individual (YPM 1190; Fig. 27), collected from late Coniacian strata of the Smoky Hill Chalk Member of the Niobrara Chalk from an outcrop near the Smoky Hill River, Logan County, Kansas, USA (Marsh, 1872; Carpenter, Dilkes & Weishampel, 1995; Everhart & Ewell, 2006). *Claosaurus agilis* was diagnosed by a unique combination of characters consisting of deltopectoral crest length less than 48% of the humeral length and with wide arcuate laterodistal corner (Fig. 27D), and ilium with supraacetabular crest as wide as 75% of the length of the central plate, with ventral apex of the crest positioned cranial to the caudal tuberosity of the ischiadic process (Fig. 27A; Prieto-Márquez, 2011b).

The skull of *Claosaurus agilis* consists of just three dentigerous fragments from the lateral wall of the left and right maxillae (Figs. 27F–27I), part of the central body and caudal ramus of the postorbital (Fig. 27B), and the apex of the coronoid process of a right dentary (Fig. 27E). The postorbital of *C. agilis* shares with that of *Eotrachodon orientalis* a gentle protuberance on the dorsal surface near the proximal region of the caudal ramus. The coronoid processes of both taxa are only moderately expanded, as is typical of non-hadrosaurid hadrosauroids. As in *E. orientalis*, the partially preserved and exposed maxillary tooth crowns of *C. agilis* display mamillated margins and a single median ridge (Figs. 27G and 27I).

The postcranium of *Claosaurus agilis* includes various partial cervical, dorsal, sacral, and caudal vertebrae, sternal plates, scapula, humeri, partial radii and ulnae, ilium, distal fragment of ischium, femora, partial tibiae, and partial pedes (Fig. 27D). Of these elements, only the ischium contains sufficient information for comparison to MSC 7949. In YPM 1190, all that remains of this element is a right distal end (Fig. 27C). As preserved, the fragment is straight, lacking the foot-like process present in MSC 7949. Nevertheless, the heavily abraded bone texture of the ischiadic fragment of YPM 1190 suggests that the fragment may be incompletely preserved; thus, the lack of a distal process in the ischium of *C. agilis* is regarded as tentative at this juncture.

### Lophorhothon atopus

**Figure 28:**
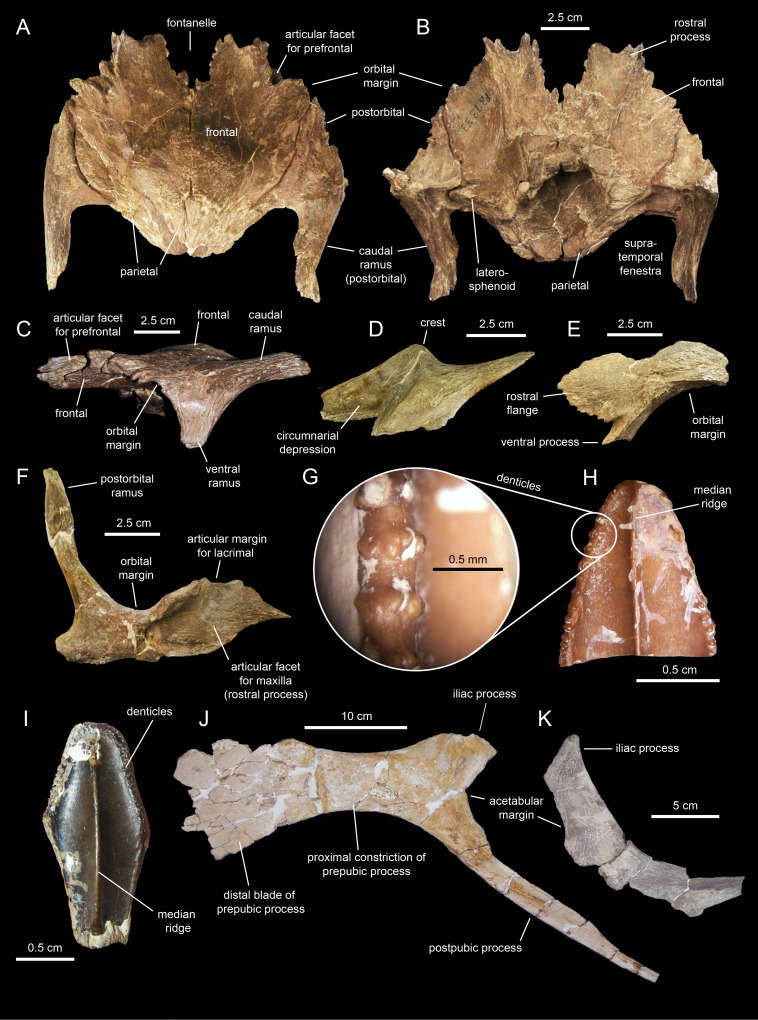
Skeletal elements of *Lophorhothon atopus*. (A, B and C) Partial skull roof and braincase (holotype FMNH 27383) in dorsal, ventral, and left lateral views. (D) Partial left nasal of FMNH 27383 in lateral view. (E) Left prefrontal of FMNH 27383 in lateral view. (F) Partial left jugal of FMNH 27383 in medial view. (G) Detail of marginal denticles of the dentary tooth in (H). (H) Apical half of a dentary tooth crown AUMP 2295 in lingual view. (I) Maxillary tooth crown of FMNH 27383 in labial view. (J) Left pubis of AUMP 2295 in lateral view. (K) Iliac process of the left ilium of FMNH 27383 in lateral view.

*Lophorothon atopus* is known from lower Campanian strata of the Mooreville Chalk, Dallas County, Alabama, USA (Langston, 1960; Fig. 28). The holotype specimen, FMNH P27383, was collected in 1946 by personnel from the Field Museum of Natural History (Chicago) at a locality designated as ADa-3 in Dallas County, Alabama (site 9 of Zangerl, 1948: pl. 3). This locality, which is owned by the Alabama Museum of Natural History in Tuscaloosa, is comprised of a series of expansive erosional gullies that expose large sections of Mooreville Chalk. Puckett (2005: fig. 5) sampled and measured a section at this locality (samples 93-1-27-1 and 93-1-27-2) and determined that the Mooreville Chalk exposures fall within the upper half of the *Acuminobrachycythere acuminata* Hazel & Paulson, 1964 Ostracode Zone and entirely within the *Globotruncanita elevata* Brotzen, 1934 Planktonic Foraminifera Zone, just above the last occurrence of the planktonic foraminifer *Dicarinella asymetrica* Sigal, 1952. Analysis of the nannoflora from site ADa-3 by personnel at the Geological Survey of Alabama showed the presence of 70–80 species including important biostratic taxa such as *Aspidolithus parcus* Noël, 1969, *Calculites obscurus* Deflandre, 1959, *Lucianorhabdus cayeuxii* Deflandre, 1959, and *Marthasterites furcatus* Deflandre, 1959, and the absence of *Bukryaster hayi* Bukry, 1969. This places the exposed Mooreville Chalk at site ADa-3 within Nannofossil Zone CC-18a and the lower half of the *Aspidolithus parcus* Perch-Nielsen, 1985 Zone. The overlap of these zones indicates an age within the early, but not earliest, Campanian (C. C. Smith, 1993, personal communication; Fig. 2).

The skull of *Lophorhothon atopus* is represented by the braincase, frontals, parietal, squamosals, postorbitals, as well as fragments of premaxilla, maxilla, nasal, prefrontal, jugal, and quadrate, and a few isolated dentary and maxillary teeth. Represented postcranial elements include the sternal plate, scapula, radius, ulna, partial ilium, partial ischia, partial pubis, tibia, fibula, and partial pes. Langston (1960) diagnosed *L. atopus* on the basis of an elevated skull with short snout, wide orbits and wide temporal fenestrae, nasal pyramidal crest similar to that of *Prosaurolophus* but located well rostral to the orbits (Fig. 28D), large fronto-nasal fontanelle (Figs. 28A and 28B), and teeth with heavily crenulated enamel surfaces and denticulate coronal margins (Figs. 28G–28I).

Various cranial and appendicular characters allow distinction of *Eotrachodon orientalis* from *Lophorhothon atopus*. The most notable of these is the absence of cranial crest in *E. orientalis*. In *L. atopus*, the medial margin of the main body of the nasal raises dorsally at the sagittal plane of the rostrum, forming a pyramidal crest rostral to the orbits (Fig. 28D). The lateral surface of the nasal rostral and ventral to this crest shows that it is excavated by the caudodorsal extent of the circumnarial depression (Fig. 28D). In contrast, the main body of the nasal of MSC 7949 is slightly convex following the gentle curvature of the rostrum (Fig. 8).

The rostroventral region of the prefrontal of *Lophorhothon atopus* bifurcates into a narrow ventral process contributing to the orbital margin and a broad flange with scalloped rostroventral edge that extends mostly rostrally (Fig. 28E). In *Eotrachodon orientalis* (Fig. 9A) and other hadrosaurids the rostroventral region of the prefrontal consists of a single broad (Saurolophini) or narrow (Lambeosaurinae) flange. In the squamosal, *L. atopus* possesses a substantially narrower quadrate cotylus than *E. orientalis*. This is reflected in the slightly greater precotyloid process length/quadrate cotylus width ratio of FMNH 27383 (0.8) than that of MSC 7949 (0.6).

The narrow shelf that roofs the medial articular surfaces of the jugal rostral processes of *Eotrachodon orientalis* (Figs. 10B and 10D) and *Lorphorhothon atopus* is obliquely oriented (Fig. 28F). In basal hadrosauroids this shelf is horizontal (e.g., *Bactrosaurus johnsoni*, AMNH 6396), whereas in saurolophid hadrosaurids there is no shelf, but instead a prominent vertical rim bounding caudally the medial articular surface of the rostral process (e.g., *Brachylophosaurus canadensis*, MOR 1071-7-31-99-281-O). Notwithstanding the fact that the ventral margin of the rostral process is incompletely preserved in both FMNH 27383 and AUMP 2295, the rostral process of the jugal of *E. orientalis* is substantially shorter (Figs. 10B and 10D) than that of *L. atopus* (Fig. 28F). This difference is also reflected in the relatively wider articular margin for the lacrimal present in *L. atopus* (Fig. 28F).

The skull roofs of *Eotrachodon orientalis* (Fig. 12A) and *Lophorhothon atopus* (Fig. 28A) show rostromedial margins of the frontals bordering caudally a fontanelle. However, the frontal margin in *L. atopus* is crenulated, surrounding a longer than wide caudal region of the fontanelle (Fig. 28A), whereas that of *E. orientalis* is straight and surrounds a wider than long caudal region of the fontanelle (Fig. 12A). No sagittal peg-like process is present in *L. atopus*. The caudal region of the frontals, near the articulation with the parietal, is only gently elevated in *E. orientalis*. In contrast, that of *L. atopus* is further elevated forming a shallow dome.

As in *Eotrachodon orientalis*, the dentary teeth of *Lorphorhoton atopus* show at least one accessory ridge at each side of the median primary carina, whereas the maxillary teeth lack accessory ridges. The margins of maxillary and dentary teeth show small papillae in both taxa; however, each individual papilla in *L. atopus* is composed of fewer (four or five) and proportionately larger knob-like protrusions (Fig. 28G) than in *E. orientalis* (Fig. 21I).

In the postcranium, the only overlapping informative and sufficiently complete elements between *Lophorhothon atopus* and *Eotrachodon orientalis* are the pubis and the ischium. The iliac process of the ischium of *L. atopus* is substantially longer (Fig. 28K) than in *E. orientalis* (Fig. 25B). Specifically, the ratio between the proximodistal length and the craniocaudal width of the distal margin of the iliac process (see Prieto-Márquez, 2008, fig. I.43, Table I.16) is 1.48 in *E. orientalis* and 1.86 in *L. atopus*. Such difference places the iliac processes of these two taxa in two different character states, according to Prieto-Márquez (2008) and Prieto-Márquez (2010a). *E. orientalis* and *L. atopus* also show differences in pubic morphology: *L. atopus* possesses a relatively shallower and longer proximal constriction of the prepubic process, with substantially more widely arcuate dorsal and ventral margins (Fig. 28J).

## Histology and Developmental Stage of the Holotype of *Eotrachodon Orientalis*

**Figure 29:**
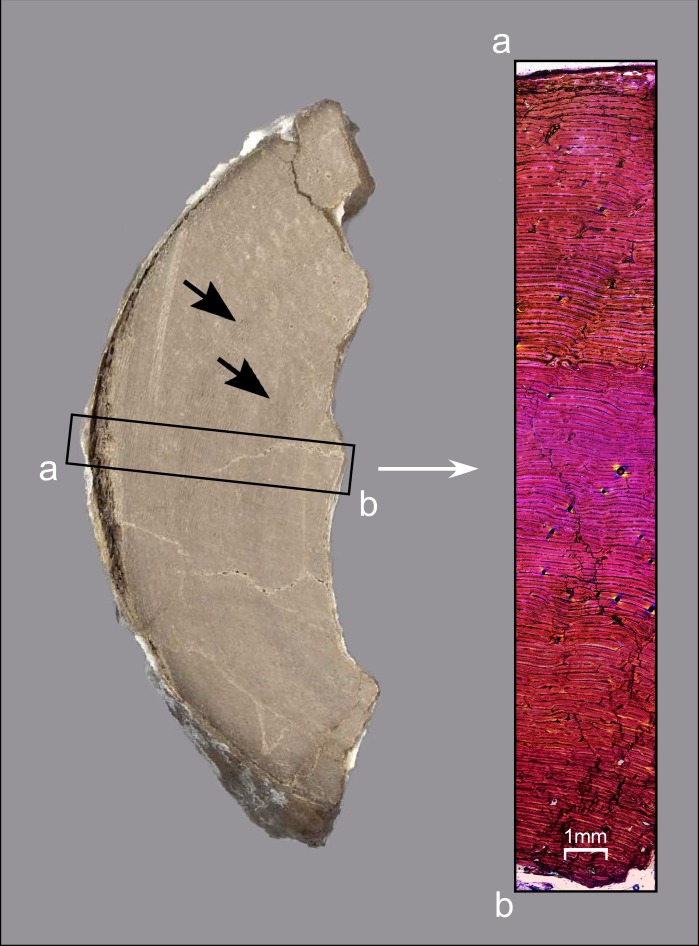
Thin-sectioned tibia of *Eotrachodon orientalis* (holotype MSC 7949). The cut-block section on the left shows two faint circumferential bands (arrows). A representative histological section compiled using nine separate images viewed with polarizing light microscopy is shown on the right. Note the apparent absence of Haversian remodeling and growth markers tradionally used in the aging of dinosaurs.

An approximately mid-diaphyseal, transversely-oriented histological hemi-section was made ~29 cm from the proximal end of the right tibia (Fig. 29). The petrographically-mounted polished section was viewed with incidental light (direct illumination) dissection microscopy (see Fig. S1 in Prieto-Márquez, Erickson & Ebersole, 2016) and using polarized light microscopy (transmitted light) (Fig. 29). These examinations show that the cortex is composed exclusively of highly vascularized plexiform bone—among the fastest forming bone types (Castanet et al., 2000; de Margerie, Cubo & Castanet, 2002; Prieto-Márquez, Erickson & Ebersole, 2016). Large primary osteons forming at the periosteal surface of the element at the time of death contributed to the rough surface texture on the bone (Francillon-Vieillot et al., 1990; Prieto-Márquez, Erickson & Ebersole, 2016). No signs of Haversian remodeling (Francillon-Vieillot et al., 1990) are evident (Prieto-Márquez, Erickson & Ebersole, 2016). These findings suggest that MSC 7949 was a young animal that was very actively growing at the time of death, and had the potential to become a larger individual in adulthood (Prieto-Márquez, Erickson & Ebersole, 2016). This is consistent with the unfused nature of part of the neurocranium (e.g., disarticulated prootics) and open neurocentral sutures in its cervical and cranial dorsal vertebrae. Furthermore, the Appalachian hadrosauroid record includes individuals that may be attributable to *Eotrachodon orientalis* with long bones at least 40% longer than those of MSC 7949. For example, a tibia (MMNS VP-6016) collected from the lower Campanian Coffee Sand at Donivan Creek, western Marietta Prentiss County, Mississippi, has an estimated length of over one meter (George Phillips, 2015, personal communication).

The actual age of MSC 7949 at the time of death is indeterminable. The tibia lacks traditional, presumably annual growth markers used to age dinosaurs (lines of arrested growth, annuli, polished lines, textural and vascular cycles; Reid, 1990; Curry, 1999; Sander, 2000; Chinsamy-Turan, 2005; Woodward & Lehman, 2009; Chinsamy et al., 2012; Erickson, 2014). Nevertheless, during our reanalysis for the present osteological description, two faint circumferential bands were observed on the cut-blocks from the histologically-sectioned tibia (Fig. 29). We are hesitant to treat them as annual growth marks since they are not evident in the microstructural architecture when viewed using polarized microscopy (Fig. 29) and, although reminiscent of dark-stained polished lines seen in Arctic ceratopsians (Erickson & Druckenmiller, 2011), they do not show topographical relief when viewed with incident light–the means by which polished lines are identified (Sander, 2000; Erickson, 2014). That said, the collective histological indices could be interpreted in two ways with regard to the age of the animal. One possibility is that MSC 7949 was less than a year old at the time of death. This assumes that the peculiar circumferential bands do not reflect annual growth marks. We feel that this interpretation should be taken with caution. First, the animal, conservatively estimated at between 4–5.1 m in total length (see Material and Phylogenetic Framework section above), is quite large for an animal less than a year in age. Second, rapidly forming plexiform bone in extant vertebrates often does not cease sufficiently to mark growth pulses (Dammers, 2002). Third, studies of extant vertebrates show that annual growth markers can be present in some taxa but absent in close relatives (Castanet, 1978; Zug & Rand, 1987; Klevezal, 1996), and can even be variably present within populations (Klevezal, 1996). Dinosaurs apparently lacking long bone growth markers have similarly been reported (Reid, 1997; Chinsamy, 1994; Chinsamy, 1995; Chinsamy et al., 2012) and hadrosaurids showing variable intra-populational expression of growth markers have also been documented (Chinsamy et al., 2012). Fourth, growth line expression, numbers and types can vary within and between elements (Horner, de Ricqlès & Padan, 2000; Dammers, 2002; Woodward, Horner & Farlow, 2014) and so may be present in parts of the tibia or other skeletal elements of MSC 7949 that were not recovered or available for destructive sampling (note: we suspect that the loss of growth markers, if present did not occur because the center of medullary cavity lies just six millimeters from where the histological section was taken). Fifth, growth markers in extant vertebrates and dinosaurs are often more prominently expressed late in ontogeny as lines of arrested growth (Francillon-Vieillot et al., 1990; Erickson & Druckenmiller, 2011; Erickson, 2014), so early forming, less distinct growth markers may not have been discernable in the MSC 7949 tibia. Sixth, environmental influences on the degree of expression of annual growth markers have been documented in extant vertebrates (Dammers, 2002), being less prevalent in animals raised in captivity under constant environmental conditions (Buffrénil, 1980), and in those from the wild experiencing only moderate annual fluctuations in climate (Zug & Rand, 1987). Notably, some extant mammals from northern temperate zones show more prevalent annual growth marker expression in their skeletons (Klevezal, 1996), which has also been shown to be the case in Arctic ornithischian dinosaurs (Erickson & Druckenmiller, 2011; Chinsamy et al., 2012). *Eotrachodon orientalis* lived at 29°30′–30°30′N paleolatitidue (Sandy Ebersole, 2015, personal communication), much lower in paleolatitude than most hadrosaurids whose histology has been documented (e.g., Horner, de Ricqlès & Padan, 2000; Chinsamy et al., 2012; Freedman Fowler & Horner, 2015; Woodward et al., 2015). Thus, it is possible that this dinosaur experienced less dramatic environmental fluctuations than those hadrosaurids from higher latitudes that have been studied and this impacted the degree of growth marker expression in its skeleton. The alternative interpretation is that MSC 7949 was several years old at the time of death. If the aforementioned dark circumferential bands are some form of annual marking, akin to the dark-stained, polished lines reported in Arctic *Pachyrhinosaurus* by Erickson & Druckenmiller (2011), abeit lacking topographical relief, it would suggest the animal met its demise late in its second year of life. The interpretation that this animal is at least several years old is more plausible given its relatively large size and the aforementioned myriad of influences on growth marker expression in dinosaurs.

## Conclusions

The basal hadrosaurid *Eotrachodon orientalis*, from the uppermost Santonian section of the Mooreville Chalk of Alabama, is the most complete and best-preserved member of this dinosaurian clade yet known from the eastern half of North America. This species shows a combination of characters shared with both basal hadrosauroids and hadrosaurids, with the most notable being a circumnarial structure with subsidiary fossae reminiscent of that of saurolophines. Characters supporting distinction of *E. orientalis* from the basal hadrosauroid *Lophorhothon atopus* (also from the Mooreville Chalk of Alabama) include the presence in the former of a slender and crestless nasal whose caudodorsal margin is not invaded by the circumnarial depression, shorter rostral process of the jugal, lack of bifurcation in the rostroventral region of the prefrontal, and shorter iliac process of the ischium. A histological analysis of the tibia of the holotype of *E. orientalis* suggests the animal was still actively growing at the time of death.

## Institutional abbreviations

ANSP: Academy of Natural Sciences of Philadelphia, Pennsylvania, USA
AUMP: Auburn University Museum of Paleontology, Auburn, Alabama, USA
CMN: Canadian Museum of Nature, Ottawa, Ontario, Canada
FMNH: Field Museum of Natural History, Chicago, Illinois, USA
MMNS: Mississippi Museum of Natural Science, Jackson, Mississippi, USA
MOR: Museum of the Rockies, Bozeman, Montana, USA
MSC: McWane Science Center, Birmingham, Alabama, USA
NHM: Natural History Museum, London, UK
ROM: Royal Ontario Museum, Toronto, Ontario, Canada
SM: Senckenberg Museum, Frankfurt am Main, Germany
SMU: Shuler Museum of Paleontology, Southern Methodist University, Dallas, Texas, USA
TMP: Royal Tyrrell Museum of Paleontology, Drumheller, Alberta, Canada
YPM: Yale Peabody Museum of Paleontology, New Haven, Connecticut, USA

## Additional Information and Declarations

### Data Deposition

The following information was supplied regarding data availability: The research in this article did not generate any raw data.

## References

[ref-1] Baird D, Horner JR (1979). Cretaceous dinosaurs of North Carolina. Brimleyana.

[ref-2] Bell PR (2011a). Redescription of the skull of *Saurolophus osborni* Brown 1912 (Ornithischia: Hadrosauridae). Cretaceous Research.

[ref-3] Bell PR (2011b). Cranial osteology and ontogeny of *Saurolophus angustirostris* from the Late Cretaceous of Mongolia with comments on *Saurolophus osborni* from Canada. Acta Palaeontologica Polonica.

[ref-4] Beneden P (1881). Sur l’arc pelvien chez les dinosauriens de Bernissart. Bulletin de l’Académie Royale de Belgique, Sciences.

[ref-5] Brett-Surman MK, Wagner JR, Carpenter K (2007). Discussion of character analysis of the appendicular anatomy in Campanian and Maastrichtian North American hadrosaurids—variation and ontogeny. Horns and Beaks. Ceratopsian and Ornithopod Dinosaurs.

[ref-6] Brotzen F (1934). Foraminiferen aus dem Senon Palästinas. Zeitschrift des Deutschen Vereins zur Erforschung Palästinas.

[ref-7] Brown B (1912). A crested dinosaur from the Edmonton Cretaceous. Bulletin of the American Museum of Natural History.

[ref-8] Brown B (1916). A new crested trachodont dinosaur *Prosaurolophus maximus*. Bulletin of the American Museum of Natural History.

[ref-9] Buffrénil V de (1980). Mise en évidence de l’incidence des conditions de milieu sur la croissance de *Crocodylus siamensis* (Schneider, 1801) et valeur des marques de croissance squelettiques pour l’évaluation de l’âge individuel. Archives de zoologie expérimentale et générale.

[ref-10] Bukry D (1969). Upper Cretaceous coccoliths from Texas and Europe. The University of Kansas Paleontological Contributions: Protista.

[ref-11] Campione NE, Evans DC (2011). Cranial growth and variation in *Edmontosaurus* (Dinosauria: Hadrosauridae): implications for latest Cretaceous megaherbivore diversity in North America. PLoS ONE.

[ref-12] Caron M, Bolli HM, Saunders JB, Perch-Nielsen K (1985). Cretaceous planktic foraminifera. Plankton Stratigraphy.

[ref-13] Carpenter K, Dilkes D, Weishampel DB (1995). The dinosaurs of the Niobrara Chalk Formation (Upper Cretaceous, Kansas). Journal of Vertebrate Paleontology.

[ref-14] Castanet J (1978). Les marques de croissance osseuse comme indicateur de láge chez les lezards. Acta Zoologica.

[ref-15] Castanet J, Curry Rogers K, Cubo J, Boisard JJ (2000). Periosteal bone growth rates in extant ratites (ostrich and emu). Implications for assessing growth in dinosaurs. Comptes Rendus de l’Académie des Sciences.

[ref-16] Chinsamy A, Rosenberg GD, Wolberg DL (1994). Dinosaur bone histology: implications and inferences. Dino Fest.

[ref-17] Chinsamy A (1995). Ontogenetic changes in the bone histology of the Late Jurassic ornithopod *Dryosaurus lettowvorbecki*. Journal of Vertebrate Paleontology.

[ref-18] Chinsamy–Turan A (2005). The Microstructure of Dinosaur Bone.

[ref-19] Chinsamy A, Thomas DB, Tumarkin-Deratzian AR, Fiorillo AR (2012). Hadrosaurs were perennial polar residents. The Anatomical Record.

[ref-20] Cope ED (1869). Remarks on *Eschrichtius polyporus*, *Hypsibema crassicauda*, *Hadrosaurus tripos*, and *Polydectes biturgidus*. Proceedings of the Academy of Natural Sciences of Philadelphia.

[ref-21] Cope ED (1871). Supplement to the “Synopsis of the extinct batrachia and reptilia of North America”. Proceedings of the Academy of Natural Sciences of Philadelphia.

[ref-22] Curry KA (1999). Ontogenetic histology of *Apatosaurus* (Dinosauria: Sauropoda): new insights on growth rates and longevity. Journal of Vertebrate Paleontology.

[ref-23] Dalla Vecchia FM (2009). *Tethyshadros insularis*, a new hadrosauroid dinosaur (Ornithischia) from the Upper Cretaceous of Italy. Journal of Vertebrate Paleontology.

[ref-25] Dammers K, Ruscillo D (2002). Using osteology for aging and sexing. Recent Advances in Ageing and Sexing Animal Bones.

[ref-24] de Margerie E, Cubo J, Castanet J (2002). Bone typology and growth rate: testing and quantifying “Amprino’s rule” in the mallard (*Anas platyrhynchos*). Comptes Rendus Biologies.

[ref-26] Deflandre G (1959). Sur les nannofossiles calcaires et leur systématique. Revue de Micropaléontologie.

[ref-27] Ebersole JA, Dean LS (2013). The history of Late Cretaceous ertebrate research in Alabama. Bulletin of the Alabama Museum of Natural History.

[ref-28] Ebersole SM, King JL (2011). A review of non-avian dinosaurs from the Late Cretaceous of Alabama, Mississippi, Georgia, and Tennessee. Bulletin of the Alabama Museum of Natural History.

[ref-29] Erickson GM (2014). On dinosaur growth. Annual Review of Earth and Planetary Sciences.

[ref-30] Erickson GM, Druckenmiller PS (2011). Longevity and growth rate estimates for a polar dinosaur: a *Pachyrhinosaurus* (Dinosauria: Neoceratopsia) specimen from the North Slope of Alaska showing a complete developmental record. Historical Biology.

[ref-31] Erickson GM, Krick BA, Hamilton M, Bourne GR, Norell MA, Lilleodden E, Sawyer WG (2012). Complex dental structure and wear biomechanics in hadrosaurid dinosaurs. Science.

[ref-32] Evans DC (2006). Nasal cavity homologies and cranial crest function in lambeosaurine dinosaurs. Palaeobiology.

[ref-33] Everhart MJ, Ewell K (2006). Shark-bitten dinosaur (Hadrosauridae) caudal vertebrae from the Niobrara Chalk (Upper Coniacian) of western Kansas. Transactions of the Kansas Academy of Science.

[ref-35] Francillon-Vieillot H, de Buffrenil V, Castanet J, Géraudie J, Meunier F-J, Sire J-Y, Zylberberg L, de Ricqlès A, Carter JG (1990). Microstructure and mineralization of vertebrate skeletal tissues. Skeletal Biomineralization: Patterns, Processes and Evolutionary Trends.

[ref-34] Freedman Fowler EA, Horner JR (2015). A new brachylophosaurin hadrosaur (Dinosauria: Ornithischia) with an intermediate nasal crest from the Campanian Judith River Formation of Northcentral Montana. PLoS ONE.

[ref-39] Gates TA, Horner JR, Hanna RR, Nelson CR (2011). New unadorned hadrosaurine hadrosaurid (Dinosauria, Ornithopoda) from the Campanian of North America. Journal of Vertebrate Paleontology.

[ref-38] Gates TA, Prieto-Márquez A, Zanno LE (2012). Mountain building triggered Late Cretaceous North American megaherbivore dinosaur radiation. PLoS ONE.

[ref-36] Gates TA, Sampson SD (2007). A new species of *Gryposaurus* (Dinosauria: Hadrosauridae) from the late Campanian Kaiparowits Formation, southern Utah, USA. Zoological Journal of the Linnean Society.

[ref-37] Gates TA, Scheetz R (2014). A new saurolophine hadrosaurid (Dinosauria: Ornithopoda) from the Campanian of Utah, North America. Journal of Systematic Palaeontology.

[ref-40] Gilmore CW (1913). A new dinosaur form the Lance Formation of Wyoming. Smithsonian Miscellaneous Collections.

[ref-41] Gilmore CW (1933). On the dinosaurian fauna of the Iren Dabasu Formation. Bulletin of the American Museum of Natural History.

[ref-42] Gilmore CW (1945). *Parrosaurus*, n.name, replacing *Neosaurus* Gilmore, 1945. Journal of Paleontology.

[ref-43] Gilmore CW, Stewart DR (1945). A new sauropod dinosaur from the Upper Cretaceous of Missouri. Journal of Paleontology.

[ref-44] Godefroit P, Bolotsky YL, Bolotsky IY (2012). Osteology and relationships of *Olorotitan arharensis*, a hollow-crested hadrosaurid dinosaur from the latest Cretaceous of Far Eastern Russia. Acta Palaeontologica Polonica.

[ref-46] Hazel JE, Paulson OL (1964). Some new ostracode species from the Austinian and Tayloran (Coniacian and Campanian) rocks of the east Texas embayment. Journal of Paleontology.

[ref-45] Head JJ (1998). A new species of basal hadrosaurid (Dinosauria: Ornithischia) from the Cenomanian of Texas. Journal of Vertebrate Paleontology.

[ref-47] Hooley R (1925). On the skeleton of *Iguanodon atherfieldensis* sp. nov., from the Wealden shales of Atherfield. Quarterly Journal of the Geological Society of London.

[ref-48] Horner JR (1983). Cranial osteology and morphology of the type specimen of *Maiasaura peeblesorum* (Ornithischia: Hadrosauridae), with discussion of its phylogenetic position. Journal of Vertebrate Paleontology.

[ref-49] Horner JR, Makela R (1979). Nest of juveniles provides evidence of family structure among dinosaurs. Nature.

[ref-50] Horner JR, de Ricqlès A, Padan K (2000). Long bone histology of the hadrosaurid dinosaur *Maiasaura peeblesorum*: growth dynamics and physiology based on an ontogenetic series of skeletal elements. Journal of Vertebrate Paleontology.

[ref-51] Horner JR, Weishampel DB, Forster CA, Weishampel DB, Dodson P, Osmólska H (2004). Hadrosauridae. The Dinosauria.

[ref-52] Hunt AP, Lucas SG (1993). Cretaceous vertebrates of New Mexico. New Mexico Museum of Natural History and Science Bulletin.

[ref-53] Huxley TH (1869). On *Hypsilophodon*, a new genus of Dinosauria. Geological Society of London, Abstracts of Proceedings.

[ref-54] Ikejiri T, Ebersole JA, Blewitt HL, Ebersole SM (2013). An overview of Late Cretaceous vertebrates from Alabama. Bulletin of the Alabama Museum of Natural History.

[ref-55] Kiernan CR (2002). Stratigraphic distribution and habitat segregation of mosasaurs in the Upper Cretaceous of western and central Alabama, with an historical review of Alabama mosasaur discoveries. Journal of Vertebrate Paleontology.

[ref-56] Kiernan CR, Schwimmer DR (2004). First record of a velociraptorine theropod (Tetanurae, Dromaeosauridae) from the eastern Gulf Coastal United States. The Mosasaur.

[ref-57] Kirkland JI (1998). A new hadrosaurid from the upper Cedar Mountain Formation (Albian-Cenomanian: Cretaceous) of eastern Utah–the oldest known hadrosaurid (Lambeosaurinae?). New Mexico Museum of Natural History and Science Bulletin.

[ref-58] Klevezal GA (1996). Recording Structures of Mammals: Determination of Age and Reconstruction of Life History.

[ref-60] Lambe LM (1914). On *Gryposaurus notabilis*, a new genus and species of trachodont dinosaur from the Belly River Formation of Alberta. Ottawa Naturalist.

[ref-61] Lambe LM (1917). A new genus and species of crestless hadrosaur from the Edmonton Formation of Alberta. Ottawa Naturalist.

[ref-59] Langston WD (1960). The vertebrate fauna of the Selma Formation of Alabama. Part 6: the dinosaurs. Fieldiana, Geology Memoirs.

[ref-62] Leidy J (1858). *Hadrosaurus foulkii*, a new saurian from the Cretaceous of New Jersey, related to *Iguanodon*. Proceedings of the Academy of Natural Sciences of Philadelphia.

[ref-63] Liu K (2007). Sequence Stratigraphy and orbital cyclostratigraphy of the Mooreville Chalk (Santonian—Campanian), northeastern Gulf of Mexico area, USA. Cretaceous Research.

[ref-65] Mancini EA, Puckett TM, Tew BH (1996). Integrated biostratigraphic and sequence stratigraphic framework for Upper Cretaceous strata of the eastern Gulf Coastal Plain, USA. Cretaceous Research.

[ref-64] Marsh OC (1870). Remarks on *Hadrosaurus minor*, *Mosasaurus crassidens*, *Leiodon laticaudus*, *Baptosaurus*, and *Rhinoceros matutinus*. Proceedings of Academy of Natural Sciences of Philadelphia.

[ref-66] Marsh OC (1872). Notice on a new species of *Hadrosaurus*. American Journal of Science.

[ref-67] Marsh OC (1892). Notice of new reptiles from the Laramie Formation. American Journal of Science.

[ref-68] McDonald AT (2012). Phylogeny of basal iguanodonts (Dinosauria: Ornithischia): an update. PLoS ONE.

[ref-69] McDonald AT, Bird J, Kirkland JI, Dodson P (2012). Osteology of the basal hadrosauroid *Eolambia caroljonesa* (Dinosauria: Ornithopoda) from the Cedar Mountain Formation of Utah. PLoS ONE.

[ref-70] McGarrity CT, Campione NE, Evans DC (2013). Cranial anatomy and variation of *Prosaurolophus maximus* (Dinosauria: Hadrosauridae). Zoological Journal of the Linnean Society.

[ref-71] Morris WJ (1970). Hadrosaurian dinosaur bills: morphology and function. Contributions in Science, Los Angeles County Museum of Natural History.

[ref-72] Noël D (1969). *Arkhangelskiella* (coccolithes Crétacés) et formes affines du Basin de Paris. Revue de Micropaléontologie.

[ref-73] Nopcsa F (1900). Dinosaurierreste aus Siebenbürgen. I. Schädel von *Limnosaurus transsylvanicus* nov. gen. et. spec. Denkschriften der königlichen Akademie der Wissenschaften, Wien.

[ref-74] Norman DB, Weishampel BD, Dodson P, Osmólska H (2004). Basal Iguanodontia. The Dinosauria.

[ref-75] Ostrom JH (1961). Cranial morphology of the hadrosaurian dinosaurs of North America. Bulletin of the American Museum of Natural History.

[ref-76] Parks WA (1922). *Parasaurolophus walkeri*, a new genus and species of crested trachodont dinosaur. University of Toronto Studies, Geological Series.

[ref-77] Parks WA (1923). *Corythosaurus intermedius*, a new species of trachodont dinosaur. University of Toronto Studies, Geological Series.

[ref-78] Perch-Nielsen K, Bolli H, Saunders JB, Perch-Neilsen K (1985). Mesozoic calcareous nannofossils. Plankton Stratigraphy.

[ref-79] Prieto-Márquez A (2005). New information on the cranium of *Brachylophosaurus canadensis* (Dinosauria, Hadrosauridae), with a revision of its phylogenetic position. Journal of Vertebrate Paleontology.

[ref-80] Prieto-Márquez A (2008). Phylogeny and historical biogeography of hadrosaurid dinosaurs.

[ref-81] Prieto-Márquez A (2010a). Global phylogeny of Hadrosauridae (Dinosauria: Ornithopoda) using parsimony and Bayesian methods. Zoological Journal of the Linnean Society.

[ref-82] Prieto-Márquez A (2010b). Global historical biogeography of hadrosaurid dinosaurs. Zoological Journal of the Linnean Society.

[ref-83] Prieto-Márquez A (2010c). The braincase and skull roof of *Gryposaurus notabilis* (Dinosauria, Hadrosauridae), with a taxonomic revision of the genus. Journal of Vertebrate Paleontology.

[ref-84] Prieto-Márquez A (2011a). Cranial and appendicular ontogeny of *Bactrosaurus johnsoni*, a hadrosauroid dinosaur from the Late Cretaceous of northern China. Palaeontology.

[ref-85] Prieto-Márquez A (2011b). Revised diagnoses of *Hadrosaurus foulkii* Leidy, 1858 (the type genus and species of Hadrosauridae Cope, 1869) and *Claosaurus agilis* Marsh, 1872 (Dinosauria: Ornithopoda) from the Late Cretaceous of North America. Zootaxa.

[ref-86] Prieto-Márquez A (2014). Skeletal morphology of *Kritosaurus navajovius* (Dinosauria: Hadrosauridae) from the Late Cretaceous of the North American south-west, with an evaluation of the phylogenetics systematics and biogeography of Kritosaurini. Journal of Systematic Palaeontology.

[ref-91] Prieto-Márquez A, Dalla Vecchia FM, Gaete R, Galobart A (2013). Diversity, relationships, and biogeography of the lambeosaurine dinosaurs from the European Archipelago, with description of the new aralosaurin *Canardia garonnensis*. PLoS ONE.

[ref-92] Prieto-Márquez A, Erickson GA, Ebersole JA (2016). A primitive hadrosaurid from southern North America and the origin and early evolution of ‘duck-billed’ dinosaurs. Journal of Vertebrate Paleontology.

[ref-88] Prieto-Márquez A, Norell MA (2010). Anatomy and relationships of *Gilmoreosaurus mongoliensis* (Dinosauria: Hadrosauroidea) from the Late Cretaceous of Central Asia. American Museum Novitates.

[ref-87] Prieto-Márquez A, Wagner JR (2009). *Pararhabdodon isonensis* and *Tsintaosaurus spinorhinus*: a new clade of lambeosaurine hadrosaurids from Eurasia. Cretaceous Research.

[ref-89] Prieto-Márquez A, Wagner JR, Eberth D, Evans DC (2014). Soft-tissue structures of the nasal vestibular region of saurolophine hadrosaurids (Dinosauria, Ornithopoda) revealed in a ‘mummified’ specimen of *Edmontosaurus annectens*. Hadrosaurs.

[ref-90] Prieto-Márquez A, Weishampel DB, Horner JR (2006). The dinosaur *Hadrosaurus foulkii*, from the Campanian of the east coast of North America, with a reevaluation of the genus. Acta Palaeontologica Polonica.

[ref-93] Puckett TM (1994). Planktonic foraminiferal and ostracode biostratigraphy of upper Santonian through lower Maastrichtian strata in central Alabama. Gulf Coast Association of Geological Societies Transactions.

[ref-94] Puckett TM (2005). Santonian-Maastrichtian planktonic foraminiferal and ostracode biostratigraphy of the northern Gulf Coastal Plain, USA. Stratigraphy.

[ref-95] Raymond DE, Osborne WE, Copeland CW, Neathery TL (1988). Alabama stratigraphy. Geological Survey of Alabama Circular.

[ref-96] Reid REH (1990). Zonal “growth rings” in dinosaurs. Modern Geology.

[ref-97] Reid REH, Farlow JO, Brett-Surman MK (1997). How dinosaurs grew. The Complete Dinosaur.

[ref-98] Sander PM (2000). Longbone histology of the Tendaguru sauropods: implication for growth and histology. Paleobiology.

[ref-99] Schwimmer DR (1997). Late Cretaceous dinosaurs in Eastern USA: a taphonomic and biogeographic model of occurrences. Dinofest International Proceedings.

[ref-100] Sereno PC (2005). Stem Archosauria. TaxonSearch.

[ref-101] Sigal J (1952). Aperçu stratigraphique sur la micropaléontologie du Crétacé. XIX Congrés Géologique International, Monographies Régionales, 1° Série, Algérie.

[ref-102] Sternberg CM (1953). A new hadrosaur from the Oldman Formation of Alberta: discussion of nomenclature. National Museum of Canada, Bulletin.

[ref-103] Sues H-D, Averianov A (2009). A new basal hadrosauroid dinosaur from the Late Cretaceous of Uzbekistan and the early radiation of duck-billed dinosaurs. Proceedings of the Royal Society B: Biological Sciences.

[ref-104] Taylor MP, Wedel MJ (2013). The effect of intervertebral cartilage on neutral posture and range of motion in the necks of sauropod dinosaurs. PLoS ONE.

[ref-105] Wiman C (1929). Die kreide. Dinosaurier aus shantung. Palaeontologia Sinica, Series C.

[ref-106] Woodward HN, Lehman TM (2009). Bone histology and microanatomy of *Alamosaurus sanjuanensis* (Sauropoda: Titanosauria) from the Maastrichtian of Big Bend National Park, Texas. Journal of Vertebrate Paleontology.

[ref-107] Woodward HN, Horner JR, Farlow JO (2014). Quantification of intraskeletal histovariability in *Alligator mississippiensis* and implications for vertebrate osteohistology. PeerJ.

[ref-108] Woodward HN, Freedman Fowler EA, Farlow JO, Horner JR (2015). *Maiasaura*, a model organism for extinct vertebrate population biology: a large sample statistical assessment of growth dynamics and survivorship. Paleobiology.

[ref-109] Wylie JA, King DT (1986). Mooreville Chalk (Upper Cretaceous), sedimentary facies and sea-level. Journal of the Alabama Academy of Science.

[ref-110] You HL, Luo Z, Shubin NH, Witmer LM, Tang ZL, Tang F (2003). The earliest-known duck-billed dinosaur from deposits of late Early Cretaceous age in northwestern China and hadrosaur evolution. Cretaceous Research.

[ref-111] Zangerl R (1948). The vertebrate fauna of the Selma Formation of Alabama: Part I, Introduction. Fieldiana Geology Memoirs.

[ref-112] Zug GR, Rand AS (1987). Estimation of age in nesting female *Iguana iguana*: testing skeletochronology in a tropical lizard. Amphibia-Reptilia.

